# Validation of multiple equations for estimating low-density lipoprotein cholesterol levels in Korean adults

**DOI:** 10.1186/s12944-021-01525-6

**Published:** 2021-09-20

**Authors:** Rihwa Choi, Mi-Jung Park, Youngju Oh, Sung Ho Kim, Sang Gon Lee, Eun Hee Lee

**Affiliations:** 1 Department of Laboratory Medicine, Green Cross Laboratories, 107, Ihyeonro 30 beon-gil, Giheng-gu, Yongin-Si, Gyeonggi-do Republic of Korea; 2 Department of Laboratory Medicine and Genetics, Samsung Medical Center, Sungkyunkwan University School of Medicine, Gangnam-gu, Seoul, Republic of Korea

**Keywords:** Low-density lipoprotein cholesterol, Friedwald equation, Calculated low-density lipoprotein, Martin/Hopkins equation, National Cholesterol Education Program, Korea

## Abstract

**Background:**

Limited data are available for validation of low-density lipoprotein cholesterol (LDL) calculation (LDL_cal_) in the adult Korean population. The aim of this study was to develop and validate a new equation for LDL_cal_ and to compare it with previous such equations in a Korean population.

**Methods:**

A new equation for LDL_cal_ was developed (LDL_Choi_). LDL_Choi_ and 11 other previously published equations were applied and compared with directly measured LDL concentration (LDL_direct_) in a development cohort (population 1), an independent validation cohort in the same laboratory (population 2), and the Korea National Health and Nutrition Examination Survey 2017 cohort (population 3).

**Results:**

Among the 12 equations, the newly-developed equation (LDL_Choi_ = total cholesterol – 0.87 x high-density lipoprotein cholesterol – 0.13 x triglycerides) had the highest intraclass correlation coefficient (ICC) and the lowest mean systemic difference and median absolute percentage error in populations 1 and 2 but not in population 3. Subgroup analysis showed good agreement between LDL_Choi_ and LDL_direct_ (ICC > 0.75) in population 2, whose LDL_direct_ < 70 mg/dL. For samples with high triglycerides (> 400 mg/dL), equation accuracy varied. Categorization concordance according to the National Cholesterol Education Program Adult Treatment Panel III criteria with the other 11 equations were less than 80%; that of LDL_Choi_ was 87.6 and 87.4% in populations 1 and 2, respectively.

**Conclusions:**

Accuracy of 12 equations for LDL_cal_ varied by cohort and subgroup based on LDL_direct_ and triglycerides. A laboratory-specific equation for LDL_cal_ and/or LDL_direct_ may be needed for accurate evaluation of LDL status.

**Supplementary Information:**

The online version contains supplementary material available at 10.1186/s12944-021-01525-6.

## Introduction

Low-density lipoprotein cholesterol (LDL) is a well-known risk factor and therapeutic target for atherosclerotic disease [1, 2]. In patients at high cardiovascular risk due to dyslipidemia, low-density lipoprotein cholesterol (LDL) reduction is recommended as the primary treatment goal, with statins as first-line therapy in national and international clinical practice guidelines [3, 4]. LDL can be measured directly (LDL_direct_) with several methods [5, 6]. The accepted gold-standard method for LDL measurement is labor-intensive, time-consuming, and expensive β-quantification after ultracentrifugation [7]. Instead, direct homogeneous assays for LDL measurement have been widely used and showed reasonable accuracy and precision compared with the reference method [5, 6]. Meanwhile, LDL is often calculated (LDL_cal_) from a lipid profile test that includes measurement of total cholesterol (TC), high-density lipoprotein cholesterol (HDL), and triglycerides (TG) [3, 8].

LDL is typically calculated using the Friedewald equation [LDL_Friedewald_ = TC – HDL – (TG/5)], which was developed based on analysis of 448 patients in 1972 [9]. The Friedewald equation is inaccurate increasingly at TG concentrations from 200 to 400 mg/dL and is regarded as invalid when at TG level > 400 mg/dL [5, 10]. Although several LDL calculation equations have been suggested as alternatives for Friedewald, studies performed in populations using different analytical measurement methods for lipid quantification reported varying equation accuracy [3, 4, 8, 11–21]. For example, in the United States, Martin et al. suggested a new equation for LDL estimation (LDL_Martin_) using an adjustable factor for TG:very-low-density lipoprotein cholesterol (VLDL) ratio based on TG and non-HDL concentration (non-HDL = TC – HDL) stratification based on lipid profiles obtained from 1,350,908 subjects [4]. Martin et al. reported that the overall concordance in guideline risk classification with LDL_direct_ was 91.7% for LDL_Martin_ and 85.4% for LDL_Friedewald_ for patients with TG lower than 400 mg/dL, but the concordance was modest (from 57.8 to 69.8%) in patients with TG ≥ 400 mg/dL [4]. An indiVidual patient meta-analysis Of statin therapY in At risk Groups: Effects of Rosuvastatin, atorvastatin and simvastatin (VOYAGER) was conceived to compare the efficacy of three statins most commonly used in clinical practice (atorvastatin, rosuvastatin, and simvastatin) using patient meta-analysis to characterize the effect of individual statin agents on lipid levels using individual patient data from pooled clinical studies [22]. The VOYAGER database contains LDL concentrations using LDL_Friedewald_ for patients with TG ≤ 400 mg/dL and LDL_direct_ using β-quantification for those with TG > 400 mg/dL [22]. However, a recent study from the VOYAGER meta-analysis database aimed to investigate the difference in LDL_cal_ when using the LDL_Martin_ and LDL_Friedewald_ equations reported that LDL_Martin_ might not be suitable for patients with TG ≥ 400 mg/dL and can result in overestimation of LDL_direct_ [13]. Considering that ethnically and ancestrally diverse populations having different allele frequencies of genetic determinants of blood lipids, for which transferable or non-transferable loci might affect lipids in gene-environment interactions [23], validation of accurate clinical applicability for all equations in various ethnic populations is needed [1, 3, 13, 20, 21].

In Korea, limited data are available on the accuracy of equations for LDL_cal_. Previous studies performed in Korea investigated the accuracy of limited numbers of equations for LDL_cal_ [8, 14, 18]. In Korea, LDL_Friedewald_ is most commonly used for LDL_cal_ [8, 24]. Therefore, the aim of this study was to evaluate the performance of a new LDL equation (LDL_Choi_) along with previous LDL estimations using 11 equations for LDL calculation in comparison with directly measured LDL in Korean adults to improve understanding of LDL calculations when direct measurement is unavailable. To the best of our knowledge, this study includes the largest number of equations to be validated in a Korean population.

## Materials and methods

### Study subjects

**Fig. 1:**
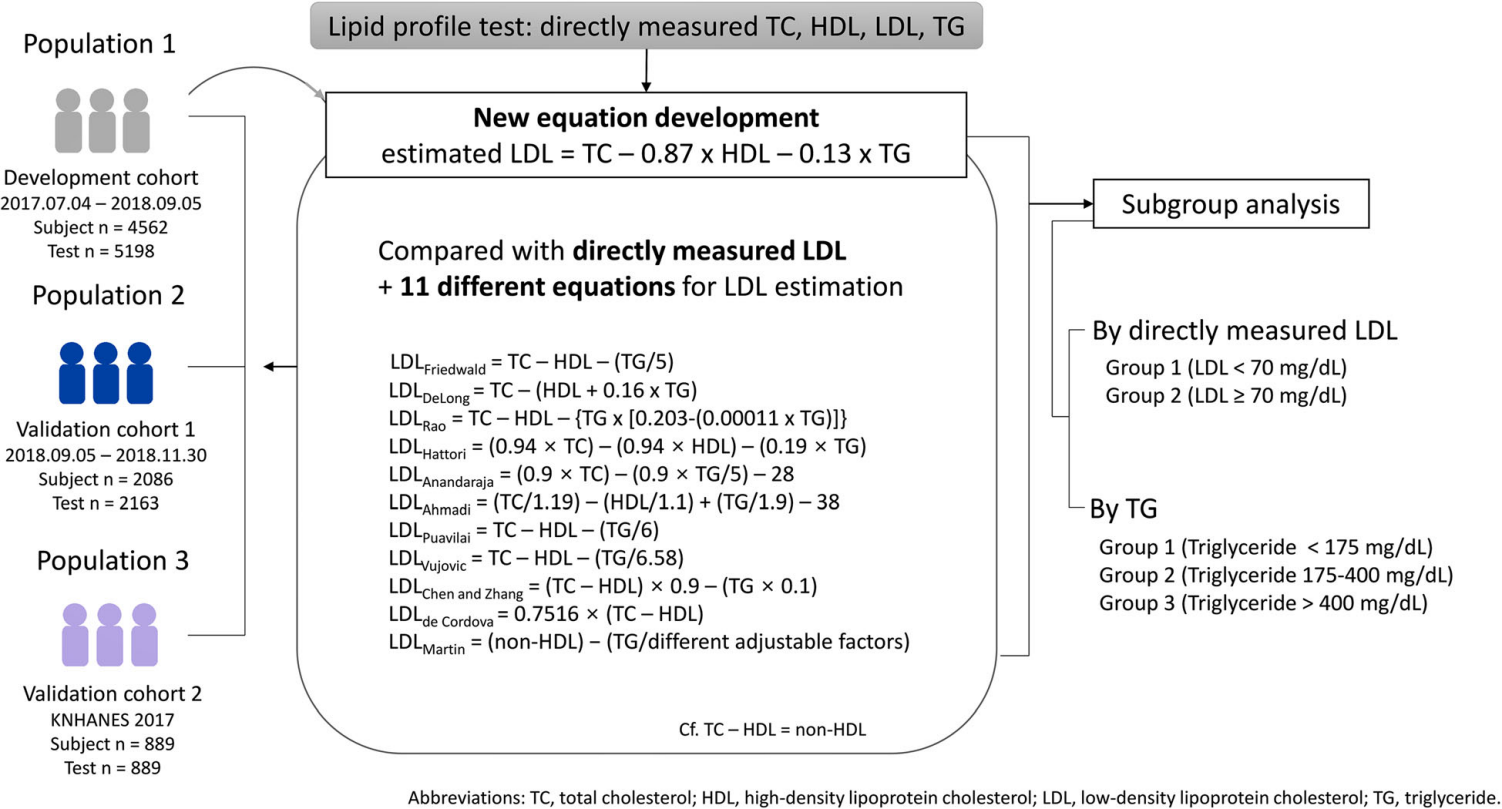
Study scheme

Clinical lipid profile test results (TC, HDL, TG, and LDL_direct_) performed between July 4, 2017 and September 5, 2018 in Korean adults (> 19 years) from the Green Cross Laboratories information system were reviewed retrospectively. Green Cross Laboratories is a referral clinical laboratory in Korea that provides clinical specimen analysis services including lipid profile tests to clinics and hospitals nationwide. Data obtained between July 4, 2017, and September 5, 2018, were used to develop a new equation for LDL calculation (LDL_Choi_) in this study, and data obtained from September 6 to November 30, 2018, were used as a separate data set (population 2, the validation cohort 1) to validate the newly-developed LDL_Choi_ equation. Data from the Korea National Health and Nutrition Examination Survey (KNHANES) 2017, a nationwide cross-sectional study regularly conducted by the Division of Chronic Disease Surveillance, Korea Centers for Disease Control and Prevention of the Ministry of Health and Welfare, was independently analyzed (population 3, the validation cohort 2) to evaluate generalizability of the 11 previously suggested equations and the LDL_Choi_ equation developed in this study. All data were anonymized before analysis. The overall study design is shown in Fig. 1. The data that support the findings of this study are available from the corresponding authors upon reasonable request.

### Analytical procedures

Serum concentrations of TC, TG, HDL, and LDL were measured using enzymatic methods with an automatic analyzer (Cobas 8000 c702, Roche, Mannheim, Germany). LDL was measured using LDL-cholesterol plus 2nd generation reagent on samples between July 4, 2017, and February 4, 2018, and LDL-cholesterol Gen.3 (Roche, Mannheim, Germany) between February 5 and November 30, 2018. TC, TG, and HDL were measured using Cholesterol Gen.2, TRIGL, and HDL-Cholesterol plus 3rd generation, respectively. The accuracy of lipid measurements was assured through the Accuracy Based Lipid Survey proficiency testing program by the College of American Pathologists and by the Lipids Standardization Program by the Centers for Disease Control, USA [25].

For population 3 (validation cohort 2, KNHANES 2017), serum TC, TG, HDL, and LDL were measured using enzymatic methods with an automatic analyzer Hitachi 7600–210 (Hitachi, Tokyo, Japan) using PureautoS CHO-N, Pureauto S TG-N, Cholestest N HDL, and Cholestest LDL reagents (Sekisui Medical, Tokyo, Japan), respectively [24].

### Selection of equations for calculated LDL

LDL was calculated using 11 previously published equations suggested by Friedewald et al. (LDL_Friedwald_) [9], DeLong et al. (LDL_DeLong_) [15], Rao et al. (LDL_Rao_) [26], Hattori et al. (LDL_Hattori_) [16], Anandaraja et al. (LDL_Anandaraja_) [17], Ahmadi et al. (LDL_Ahmadi_) [27], Puavilai et al. (LDL_Puavilai_) [11], Chen and Zhang et al. (LDL_Chen and Zhang_) [19], Vujovic et al. (LDL_Vujovic_) [28], de Cordova et al. (LDL_de Cordova_) [29], and Martin et al. (LDL_Martin_) [4]. And a new equation (LDL_Choi_) using multiple linear regression analysis of TC, TG, HDL and LDL_direct_ values using the development cohort. All 12 of these equations were compared with LDL_direct_ measured using a homogenous enzymatic method for development and independent validation cohorts, which including populations of the KNHANES 2017 cohort.

### Statistical analysis

The distribution of continuous variables is described as mean with standard deviation (for normally distributed variables) or as median with interquartile range (for skewed variables) and compared using an independent t-test and Wilcoxon rank sum test, respectively. Categorical variables are reported as observed number and percentage as calculated using the Chi-square test.

Agreement and accuracy of the 12 equations for estimating LDL_cal_ were investigated using LDL_direct_ as the reference value. The intraclass correlation coefficient (ICC) was calculated to compare the degrees of agreement between the 12 equations and LDL_direct_. The level of agreement was defined according to ICC value; good agreement was when ICC > 0.75, and moderate agreement was when 0.5 < ICC < 0.75 [18]. Bland–Altman plots were assessed to compare LDL_direct_ and multiple equations for LDL_cal_.

Subgroup analysis was performed to investigate whether discordant LDL quantification occurs when LDL cholesterol is measured or calculated with different assays, especially in patients with low LDL cholesterol or hypertriglyceridemia [1]. Data were classified into two groups (LDL < 70 mg/dL in LDL group 1 and LDL ≥ 70 mg/dL in LDL group 2) for subgroup analysis to investigate the performance of each equation based on LDL concentration. Data were classified into three groups according to serum TG concentration as follows: < 175, 175–400, and > 400 mg/dL [5, 10] for subgroup analysis to investigate the performance of each equation based on TG concentration.

Agreement according to category based on the National Cholesterol Education Program (NCEP) Adult Treatment Panel III (ATP III) between LDL_cal_ estimated using the 12 equations and LDL_direct_ concentration was also assessed. Optimal LDL concentration for < 100 mg/dL, above optimal for 100–129 mg/dL, borderline high for 130–159 mg/dL, high for 160–189 mg/dL, and very high for ≥190 mg/dL were applied [30]. Equations for LDL_cal_ and NCEP ATP III criteria for each LDL_cal_ are provided as online Supplementary Material S1.

Statistical analysis was executed using MedCalc software for Windows, version 19.1.3 (MedCalc Software bv, Ostend, Belgium; https://www.medcalc.org; 2019). Statistical significance was defined as a *P*-value less than 0.05.

## Results

A total of 5198 lipid profile test results from 4562 Korean adults was obtained between July 4, 2017, and September 5, 2018, and was used to develop a new equation for LDL calculation (LDL_Choi_). Using multiple linear regression analysis of the development cohort data with 5198 lipid profile test results, a new equation for estimated LDL_cal_ was developed as follows: LDL_Choi_ = TC – 0.87 x HDL – 0.13 x TG. Between September 6 and November 30, 2018, 2163 lipid profile test results from 2086 Korean adults were obtained in the same laboratory and used as a separate data set (population 2, validation cohort 1), with which LDL_direct_ was measured with a different reagent generation to validate the newly developed LDL_Choi_ equation. For another independent validation cohort (population 3, validation cohort 2), a total of 889 lipid profile test results from 889 Korean adults was used. General characteristics and lipid profile test results in the three independent populations in this study are summarized in Table 1. In the KNHANES 2017 cohort (validation cohort 2, population 3), the proportion of male subjects was greater than that of female subjects, there were no subjects with TG < 175 mg/dL, and there were more patients with TG > 400 mg/dL compared to the other cohorts.

**Table 1:** Characteristics of study cohorts

Variable	Population 1 (Development cohort)			Population 2 (Validation cohort 1)			Population 3 (Validation cohort 2, KNHANES 2017)		
Gender distribution	Total	Men	Women	Total	Men	Women	Total	Men	Women
Number of subjects	4562	2202	2360	2086	1016	1070	889	588	301
Age, years (median, IQR)	55.7 (46.3–63.4)	55.2 (45.9–62.7)	56.3 (46.8–64.0)	55.4 (46.3–63.8)	53.9 (45.5–62.1)	56.7 (47.6–65.7)	53.3 (41.0–64.0)	49.0 (39.0–60.0)	58.0 (49.0–70.0)
Lipid profile test result of all specimens (Number of tests)	5198	2570	2628	2163	1062	1101	889	588	301
Total cholesterol, mg/dL, median (IQR)	180 (153 to 209)	176 (150 to 205)	184 (157 to 212)	177 (152 to 209)	173.5 (148 to 205)	180 (155 to 211)	210 (183 to 239)	209 (183 to 239)	211 (184 to 240)
HDL, mg/dL, median (IQR)	52 (43 to 63)	48 (40 to 57)	57 (47 to 69)	53 (44 to 65)	49 (41 to 59)	58 (48 to 70)	41 (36 to 47)	40 (36 to 46)	42 (37 to 49)
TG, mg/dL, median (IQR)	131 (90 to 196)	152 (102 to 232)	114 (81 to 165)	127 (88 to 187)	145 (101 to 217)	113 (80 to 160)	259 (223 to 337)	266 (226 to 354)	249 (220 to 307)
LDL_direct_, mg/dL, median (IQR)	111 (86 to 139)	109 (84 to 136)	112 (88 to 142)	108 (83 to 138)	106 (80 to 136)	111 (86 to 139)	119 (92 to 146)	118.5 (93 to 145)	120 (91 to 147)
Subgroup based on LDL_direct_ (n, %)									
LDL subgroup 1 (LDL < 70 mg/dL)	581 (11.2%)	357 (13.9%)	224 (8.5%)	287 (13.3%)	164 (15.4%)	123 (11.2%)	83 (9.3%)	64 (10.9%)	19 (6.3%)
LDL subgroup 2 (LDL ≥ 70 mg/dL)	4617 (88.8%)	2213 (86.1%)	2404 (91.5%)	1876 (86.7%)	898 (84.6%)	978 (88.8%)	806 (90.7%)	524 (89.1%)	282 (93.7%)
Subgroup based on TG (n, %)									
TG subgroup 1 (TG < 175 mg/dL)	3566 (68.6%)	1517 (59.0%)	2049 (78.0%)	1546 (71.5%)	669 (63.0%)	877 (79.7%)	0 (0.0%)	0 (0.0%)	0 (0.0%)
TG subgroup 2 (TG 175–400 mg/dL)	1330 (25.6%)	819 (31.9%)	511 (19.4%)	542 (25.1%)	336 (31.6%)	206 (18.7%)	758 (85.3)	489 (83.2%)	269 (89.4%)
TG subgroup 3 (TG > 400 mg/dL)	302 (5.8%)	234 (9.1%)	68 (2.6%)	75 (3.5%)	57 (5.4%)	18 (1.6%)	131 (14.7%)	99 (16.8%)	32 (10.6%)

*HDL* high-density lipoprotein, *IQR* interquartile range, *KNHANES* Korea National Health and Nutrition Examination Survey, *LDL* low-density lipoprotein, *TG* triglycerides In population 3 (KNHANES 2017 cohort), all subjects had TG ≥ 200 mg/dL

**Fig. 2:**
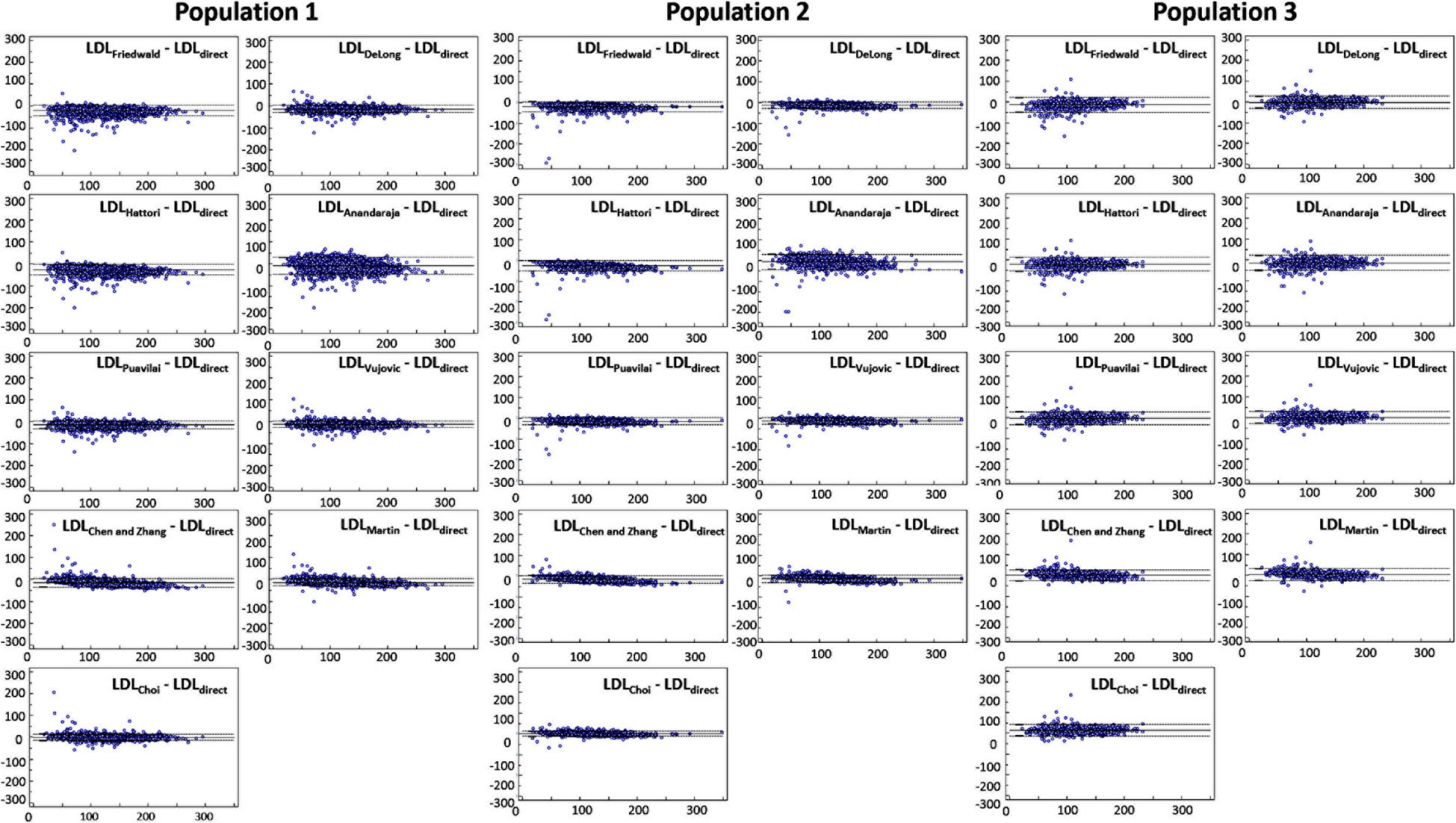
Bland–Altman plots for nine equations with directly measured LDL concentration (excluding LDL_Rao_, LDL_Ahmadi_, and LDL_de Cordova_ equations). X-axis represents directly measured LDL concentration (LDL_direct_) and Y axis represents difference between calculated LDL and directly measured LDL (LDL_direct_)

Including the newly developed equation (LDL_Choi_), 12 equations for LDL_cal_ were analyzed. In populations 1 (development cohort) and 2 (validation cohort 1), the mean value of estimated LDL_cal_ in total subjects (men and women) showed negative bias in comparison with LDL_direct_ except for LDL_Ahmadi_ (Supplementary Fig. S1 and Table S1). In population 3 (validation cohort 2, KNHANES 2017), estimated LDL_Ahmadi_ showed a mean value greater than two times that of LDL_direct_. Because LDL_Rao_, LDL_Ahmadi_, and LDL_de Cordova_ equations had several outliers with large differences between estimated LDL and LDL_direct_, Bland–Altman plots for only nine of the equations are shown in Fig. 2.

Among the 12 equations for LDL_cal_, the newly developed LDL_Choi_ equation showed the highest ICC in the development cohort (population 1) and in validation cohort 1 (population 2). However, LDL_DeLong_, LDL_Rao_, LDL_Puavilai_, LDL_Chen and Zhang_, LDL_Vujovic_, and LDL_Martin_ showed a higher ICC than did LDL_Choi_ in validation cohort 2 (population 3, KNHANES 2017). All equations except LDL_Ahmadi_ showed good agreement with ICC > 0.75. LDL_Choi_ showed the lowest mean systemic difference in populations 1 and 2, although other equations showed lower systemic differences than LDL_Choi_ in validation cohort 2 (population 3, KNHANES 2017). The limits of agreement with LDL_direct_ and LDL_cal_ from each equation are summarized in Supplementary Table S2. LDL_Choi_ showed the lowest absolute percentage error for LDL_direct_ estimation in populations 1 and 2 but not in population 3.

In subgroup analysis according to LDL_direct_ concentration, the equations showed variable accuracy in comparison with LDL_direct_ (Supplementary Tables S3 to S4 and Supplementary Figs. S2). The top three equations that showed high ICC, low mean systemic differences, or low absolute percentage errors are presented in color. In LDL group 1 (LDL_direct_ < 70 mg/dL), no equation showed good agreement (ICC > 0.75) with LDL_direct_ except LDL_Choi_ in women in population 2 (ICC = 0.81). Different equations ranked as top three among subgroups. In LDL group 1 (LDL_direct_ < 70 mg/dL), the newly developed LDL_Choi_ equation was included frequently in the top three equations for ICC, mean systemic differences, and absolute percentage errors in populations 1 and 2 but not in population 3.

In subgroup analysis according to TG concentration (Supplementary Tables S5 and S6 and Supplementary Fig. S3), all equations except LDL_de Cordova_ showed good agreement (ICC > 0.75) with LDL_direct_ in TG group 1 (TG < 175 mg/dL). In TG group 2 (TG 175–400 mg/dL), all equations showed good agreement with LDL_direct_ except LDL_Ahmadi_ in all three populations. In TG group 3 (TG > 400 mg/dL), the LDL_DeLong_, LDL_Vujovic_, LDL_Chen and Zhang_, LDL_Martin_, and LDL_Choi_ equations showed good agreement with LDL_direct_ in all three populations.

Considering median and 95th percentile values of absolute percentage errors, no equations showed values ≤12.0% in all three populations. In subgroup analysis by LDL_direct_ concentration, only LDL_Choi_ showed median absolute percentage error values ≤12.0% in populations 1 and 2 in LDL group 1 (LDL < 70 mg/dL). In subgroup analysis by TG concentration, no equation showed ≤12.0% error for 95th percentile values of absolute percentage error in TG group 2 (TG 175–400 mg/dL) or TG group 3 (TG > 400 mg/dL), while only LDL_Choi_ showed ≤12.0% in TG group 1 (TG < 175 mg/dL) in populations 1 and 2.

**Fig. 3:**
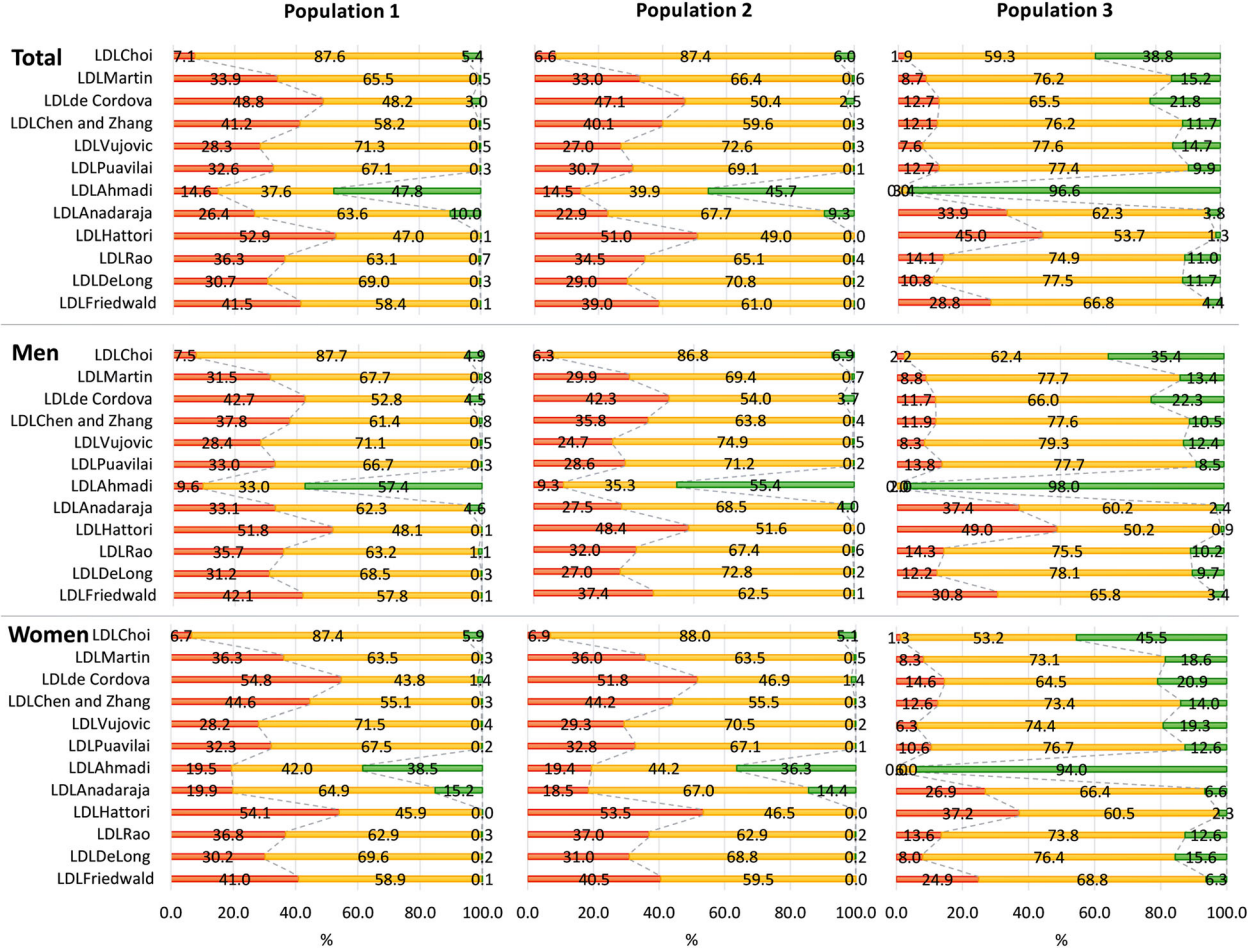
Overall agreement of categorization according to the National Cholesterol Education Program Adult Treatment Panel III (NCEP ATP III) between calculated LDL and directly measured LDL. X-axis represents percentage of agreement. Red color represents underestimated, yellow color represents concordant, and green color represents overestimated patient categories according to NCEP ATP III in comparison with those categories assigned by directly measured LDL concentration

Overall concordant categorization agreement according to the NCEP ATP III between LDL_cal_ estimated using the 12 equations and LDL_direct_ concentration is summarized in Fig. 3. The concordance rate varied among equations for LDL_cal_. Categorization concordance according to the NCEP criteria in the other 11 equations was less than 80% in all three populations. However, LDL_Choi_ showed the highest concordance with LDL_direct_ (86.8–88.0%) in populations 1 and 2, followed by LDL_Vujovic_. In population 3, LDL_Choi_ overestimated 38.8% of results, while the other equations except for LDL_Hattori_ showed higher concordance than LDL_Choi_.

## Discussion

In this study, a new equation (LDL_Choi_) for LDL estimation is developed and validated in agreement and accuracy with LDL_direct_ as the reference value using two independent cohorts in comparison with 11 equations established in previous studies in a Korean population. Although several studies have been reported to validate several of the 11 equations in the Korean population, to the best of our knowledge, this study includes the largest number of concurrent equations for validation of estimating LDL_cal_.

The LDL_Choi_ showed good agreement with LDL_direct_ in populations 1 and 2, which contained lipid profile data from different periods using different generations of reagents in the same laboratory. However, in population 3 data from KNHANES 2017, all other equations showed higher values for ICCs and lower values for systemic differences and absolute percentage errors. Different equations showed different performance among the three populations in this study. These findings might be due to various factors in measurement of LDL_direct_, including analytical methods with use of different reagents and instruments as well as different ethnicities and populations with various health conditions [8]. These findings suggest that laboratory-specific equations would provide more accurate values of estimated LDL_cal_ rather than use of LDL_Friedwald_.

In the era of personalized medicine, patient-specific risk estimation is important for health care [3]. In this study, categorization concordance according to the NCEP criteria in the previously developed 11 equations was less than 80% in all three populations. These findings suggest that LDL_cal_ concentration was under- or over-estimated for more than 20% of patients. Previous studies regarding evaluation and validation of various equations for LDL_cal_ estimation usually included specimen results with TG concentration < 400 mg/dL based on the limitation of accuracy for the LDL_Friedewald_ equation [1]. In the present study, the accuracy of 12 equations of LDL_cal_ was evaluated using 511 test results with > 400 TG mg/dL. Although the highest concordance rate according to NCEP ATP III criteria was observed as 81.3% in population 1 and 75.5% in population 2 for LDL_Choi_ in TG subgroup 3 (TG > 400 mg/dL), the concordance according to NCEP ATP III criteria was 71.0% for LDL_Friedwald_, 55.7% for the newly developed LDL_Choi_ equation, 77.1% for LDL_Vujovic_ and 76.3% for LDL_Puavilai_ in population 3 for TG subgroup 3 (TG > 400 mg/dL). These findings confirmed the validation of equations for estimated LDL_cal_ [1, 14, 18]. Considering that the NCEP ATP III criteria could affect the management plan, accurate assignment of patient categorization with accurate LDL estimation is needed [4].

According to NCEP criteria, the total error of LDL measurements should be within 12% of the true value [7]. In this study, although median values of absolute percentage errors were ≤ 12% in some equations, when considering both the median and 95th percentile values of absolute percentage errors ≤12%, no equations met these requirements. Only the LDL_Choi_ equation met the median and 95th percentile values of absolute percentage errors ≤12% in specimens with LDL ≥ 70 mg/dL in the subgroup analysis for LDL concentration groups and in specimens with TG < 175 mg/dL in the subgroup analysis for TG concentration in populations 1 and 2. This finding confirmed that previous estimations of LDL might not be applicable in samples with low LDL or high TG concentration, and LDL_direct_ is needed for accurate assessment of LDL concentration [31, 32].

### Comparisons with other equations

A recent randomized clinical trial (the Further Cardiovascular Outcomes Research with Proprotein convertase subtilisin/kexin type 9 (PCSK9) Inhibition in Subjects with Elevated Risk Trial) using data collected from 2013 to 2016, from 1242 centers in 49 countries including Korea, reported that the LDL_Martin_ equation more closely approximated the gold-standard preparative ultracentrifugation values than did LDL_Friedewald_ [3]. In the present study, LDL_Martin_ showed good agreement with LDL_direct_ (ICC = 0.92), with mean systemic differences from − 11.1 to 5.7% (− 12.5 to 4.4 mg/dL) in the three populations. In the subgroup analysis, LDL_Martin_ was in the top three equations for estimated LDL in both subgroups (LDL_direct_ < 70 mg/dL or ≥ 70 mg/dL, Supplementary Tables S3 and S4). In subgroups of TG, LDL_Martin_ was in the top three equations for estimated LDL (TG group 2 with 175–400 mg/dL and TG group 3 with > 400 mg/dL), except for TG group 1 patients whose TG < 175 mg/dL. In patients with TG < 175 mg/dL, LDL_Choi_, LDL_Vujovic_, and LDL_deLong_ frequently were listed in the top three equations (Supplementary Tables S5 and S6). Previous studies about LDL_cal_ have been performed in various ethnic cohorts with different characteristics, such as range of lipid concentrations and measurement methods for LDL_direct_ (Table 2). All equations compared in this study varied in design factors, such as target population or specific characteristics, by developer. Because of this, equation comparisons should be interpreted carefully. In the present study, LDL_cal_ using LDL_Hattori_ showed a very high result in population 3 (KNHANES 2017, Validation cohort 2). Meanwhile, as in Supplementary Tables S1 to S6 and Figs. 2 and 3, LDL_Ahmadi_ was different from the other equations. Each of the 12 equations for LDL_cal_ reflected a different effect of TG in the population data used. Equations of LDL_Hattori_ and LDL_Ahmadi_ were designed using subjects with TG ≤ 400 mg/dL and TG < 100 mg/dL, respectively. While LDL_Vujovic_ was also from subjects with TG ≤ 400 mg/dL, it was based on LDL_direct_ values measured using an automated enzymatic method. Various measurement methods of lipids can vary in accuracy, convenience, and cost [15]. Considering that current clinical laboratories usually determine LDL_direct_ by automatic enzymatic methods and physicians manage patients with strategies based on these values from clinical laboratories, LDL_cal_ obtained from LDL_direct_ calculated by automatic enzymatic methods might better classify patients at risk [28]. Future studies are needed to define the accuracy and clinical impacts of various equations in large numbers of populations.

**Table 2:** Characteristics of the 12 equations for calculated LDL

LDL_cal_	Pub. year	N of subjects or specimens	Studied region	Specimen type	TG concentration characteristics	Measurement method for LDL quantification	Equations
LDL_Friedwald_	1972	448 subjects	USA	Plasma	TG ranged 20–2502 mg/dL.	Ultracentrifugation	TC – HDL – (TG / 5)
LDL_DeLong_	1986	10,483 subjects	USA	Plasma or serum	964 subjects whose TG > 400 mg/dL were included.	Ultracentrifugation	TC – (HDL + 0.16 x TG)
LDL_Rao_	1988	196 sera	Kuwait	Serum	33 subjects defined as high TG (> 204 mg/dL) were included.	Ultracentrifugation	TC – HDL – {TG x [0.203 – (0.00011 x TG)]}
LDL_Hattori_	1998	2179 subjects	Japan	Plasma	Subjects with TG > 400 mg/dL were excluded. ^a^	Ultracentrifugation	(0.94 x TC) – (0.94 x HDL) – 0.19 x TG
LDL_Anadaraja_	2005	2008 subjects	India	Plasma	153 subjects whose TG > 350 mg/dL were included.	Ultracentrifugation	(0.9 x TC) – (0.9 X TG / 5) – 28
LDL_Ahmadi_	2008	230 sera from 115 subjects	Iran	Serum	All subjects had TG < 350 mg/dL. ^a^ Equations were produced using data from patients with TG < 100 mg/dL.	Automated enzymatic method	(TC / 1.19) – (HDL / 1.1) + (TG / 1.9) – 38
LDL_Puavilai_	2009	999 sera	Thailand	Serum	80 subjects whose TG > 300 mg/dL were included.	Automated enzymatic method	TC – HDL – (TG / 6)
LDL_Vujovic_	2010	2053 subjects	Serbia	Serum	Subjects with TG > 400 mg/dL were excluded. ^a^	Automated enzymatic method	TC – HDL – (TG/ 6.58)
LDL_Chen and Zhang_	2010	2180 subjects	China	Serum	480 subjects whose TG > 400 mg/dL were included.	Automated enzymatic method	(TC – HDL) × 0.9 – (TG × 0.1)
LDL_de Cordova_	2013	10,664 subjects	Brazil	Serum	470 subjects whose TG > 400 mg/dL were included.	Automated enzymatic method	0.7516 x (TC – HDL)
LDL_Martin_	2013	1,350,908 subjects	USA	Serum	10,124 subjects whose TG > 400 mg/dL were included.	Ultracentrifugation	(TC – HDL) – (TG / different adjustable factors)
LDL_Choi_	This study	Development cohort: 5198 sera from 4562 subjects Validation cohort 1: 2163 sera from 2086 subjects Validation cohort 2: 889 sera from 889 subjects	South Korea	Serum	302 sera with TG > 400 mg/dL were included. 75 sera with TG > 400 mg/dL were included. All subjects had TG ≥ 200 mg/dL (Among them, 131 had TG > 400 mg/dL)	Automated enzymatic method ^b^	TC – 0.87 x HDL – 0.13 x TG

*HDL* high-density lipoprotein, *LDL* low-density lipoprotein, *Pub.* publication, *TC* total cholesterol, *TG* triglycerides

Equation of LDL_Puavilai_ was similar with that of LDL_DeLong_. LDL_de Cordova_ does not include TG concentration

^a^These equations did not include data from patients whose TG > 400 mg/dL

^b^Automated c702 analyzers using LDL-cholesterol plus 2nd generation reagent for the development cohort and LDL-cholesterol 3rd generation reagent for the validation cohort 1 were used (Cobas 8000 c702, Roche, Germany). For validation cohort 2, Hitachi 7600–210 (Hitachi, Tokyo, Japan) analyzer using Cholestest LDL reagent (Sekisui Medical, Tokyo, Japan) was used

### Strengths and limitations

The strengths of this study are development of a new calculation equation (LDL_Choi_) using a relatively large number of Korean subjects and for which accuracy were compared with that of multiple other equations. Furthermore, calculators for the 12 equations are provided as an online supplementary material and can help physicians and researchers validate LDL_cal_ in different ethnic populations. The limitations of this study are the retrospective design and the lack of clinical information including detailed history and physical examination and other laboratory and image studies associated with cardiovascular and other diseases, which is out of the scope of this study. The results of this study might not be generalizable to other populations as all data were from Korean adults. To enhance generalizability, future studies using different ethnic populations and ages are needed. Future studies on the clinical impact of the practice using large numbers of patients with various LDL_cal_ and LDL_direct_ in various ethnicities are needed.

## Conclusions

In conclusion, a new LDL_Choi_ equation for estimated LDL_cal_ was developed and validated in a Korean population and compared with 11 previous equations using LDL_direct_ as a reference method. The accuracy of LDL_Choi_ was highest in laboratory-specific populations. Considering that the accuracy varied according to cohort (population), LDL_direct_, and TG concentration, direct measurement of LDL is needed for accurate evaluation of LDL. This study can help to expand our knowledge about LDL_cal_ in Korean populations and to improve test utilization.

## Supplementary Information

**Additional file 1: Supplementary Material S1.** LDL calculators for the 12 equations.

**Additional file 2: Supplementary Table S1.** Intraclass correlation coefficient and systemic differences among the 12 equations in comparison with directly measured LDL. **Supplementary Table S2.** Limits of agreement and absolute error among the 12 equations in comparison with directly measured LDL. **Supplementary Table S3.** Intraclass correlation coefficient and systemic differences among the 12 equations in comparison with directly measured LDL (LDL_direct_) by subgroup of LDL concentration. **Supplementary Table S4.** Limits of agreement and absolute error among the 12 equations in comparison with directly measured LDL by subgroup of LDL concentration. **Supplementary Table S5.** Intraclass correlation coefficient and systemic differences among the 12 equations in comparison with directly measured LDL according to subgroup by triglyceride (TG) concentration. **Supplementary Table S6.** Limit of agreement and absolute percentage errors among the 12 equations in comparison with directly measured LDL according to subgroups by triglyceride (TG) concentration. **Supplementary Fig. S1.** Bland–Altman plots for the 12 equations with directly measured LDL concentration. **Supplementary Fig. S2.** Overall agreement of categorization according to the NCEP ATP III between calculated LDL and directly measured LDL by LDL subgroup. **Supplementary Fig. S3.** Overall agreement of categorization according to the NCEP ATP III between calculated LDL and directly measured LDL by TG subgroup.

## Data Availability

The datasets used and analyzed during the current study are available from the corresponding authors on reasonable request.

## Abbreviations

ATP III: Adult treatment panel III
HDL: High-density lipoprotein cholesterol
ICC: Intraclass correlation coefficient
IRB: Institutional review board
KNHANES: Korea national health and nutrition examination survey
LDL: Low-density lipoprotein cholesterol
LDL_Ahmadi_: Low-density lipoprotein cholesterol calculation using equation by Ahmadi et al.
LDL_Anandaraja_: Low-density lipoprotein cholesterol calculation using equation by Anandaraja et al.
LDL_cal_: Low-density lipoprotein cholesterol calculation
LDL_Chen and Zhang_: Low-density lipoprotein cholesterol calculation using equation by Chen and Zhang et al.
LDL_Choi_: Low-density lipoprotein cholesterol calculation using a new equation by Choi et al.
_LDLde Cordova_: Low-density lipoprotein cholesterol calculation using equation by de Cordova et al.
LDL_DeLong_: Low-density lipoprotein cholesterol calculation using equation by DeLong et al.
LDL_direct_: Directly measured low-density lipoprotein cholesterol
LDL_Friedewald_: Low-density lipoprotein cholesterol calculation using equation by Friedewald et al.
LDL_Hattori_: Low-density lipoprotein cholesterol calculation using equation by Hattori et al.
LDL_Martin_: Low-density lipoprotein cholesterol calculation using equation by Martin et al.
LDL_Puavilai_: Low-density lipoprotein cholesterol calculation using equation by Puavilai et al.
LDL_Rao_: Low-density lipoprotein cholesterol calculation using equation by Rao et al.
LDL_Vujovic_: Low-density lipoprotein cholesterol calculation using equation by Vujovic et al.
NCEP: National cholesterol education program
PCSK9: Proprotein convertase subtilisin/kexin type 9
TC: Total cholesterol
TG: Triglycerides
VLDL: Very-low-density lipoprotein cholesterol
VOYAGER: indiVidual patient meta-analysis Of statin therapY in At risk Groups: Effects of Rosuvastatin, atorvastatin and simvastatin
