# Supplementary material for: Validation of multiple equations for estimating low-density lipoprotein cholesterol levels in Korean adults
Source: Lipids Health Dis. 2021 Sep 20;20:111. doi: 10.1186/s12944-021-01525-6 (PMC8453999; doi:10.1186/s12944-021-01525-6)
Supplement: Supplementary file 2 — Additional file 2: Supplementary Table S1. Intraclass correlation coefficient and systemic differences among the 12 equations in comparison with directly measured LDL. Supplementary Table S2. Limits of agreement and absolute error among the 12 equations in comparison with directly measured LDL. Supplementary Table S3. Intraclass correlation coefficient and systemic differences among the 12 equations in comparison with directly measured LDL (LDLdirect) by subgroup of LDL concentration. Supplementary Table S4. Limits of agreement and absolute error among the 12 equations in comparison with directly measured LDL by subgroup of LDL concentration. Supplementary Table S5. Intraclass correlation coefficient and systemic differences among the 12 equations in comparison with directly measured LDL according to subgroup by triglyceride (TG) concentration. Supplementary Table S6. Limit of agreement and absolute percentage errors among the 12 equations in comparison with directly measured LDL according to subgroups by triglyceride (TG) concentration. Supplementary Fig. S1. Bland–Altman plots for the 12 equations with directly measured LDL concentration. Supplementary Fig. S2. Overall agreement of categorization according to the NCEP ATP III between calculated LDL and directly measured LDL by LDL subgroup. Supplementary Fig. S3. Overall agreement of categorization according to the NCEP ATP III between calculated LDL and directly measured LDL by TG subgroup. [file 12944_2021_1525_MOESM2_ESM.zip › Supplementary Tables Figure Legends.docx]

**Supplementary Table S1**. Intraclass correlation coefficient and systemic differences among the 12 equations in comparison with directly measured LDL

|  | **Population 1** | | | | | | **Population 2** | | | | | | **Population 3** | | | | | |
| --- | --- | --- | --- | --- | --- | --- | --- | --- | --- | --- | --- | --- | --- | --- | --- | --- | --- | --- |
|  | **Total**  **(Test n=5198)** | | **Men**  **(Test n=2570)** | | **Women**  **(Test n=2628)** | | **Total**  **(Test n=2163)** | | **Men**  **(Test n=1062)** | | **Women**  **(Test n=1101)** | | **Total**  **(Test n=889)** | | **Men**  **(Test n=588)** | | **Women**  **(Test n=301)** | |
| **Intraclass correlation** | **Coefficient** | **95%CI** | **Coefficient** | **95%CI** | **Coefficient** | **95%CI** | **Coefficient** | **95%CI** | **Coefficient** | **95%CI** | **Coefficient** | **95%CI** | **Coefficient** | **95%CI** | **Coefficient** | **95%CI** | **Coefficient** | **95%CI** |
| LDL_Friedwald_ | 0.84 | 0.01 to 0.95 | 0.81 | 0.01 to 0.94 | 0.88 | -0.01 to 0.97 | 0.86 | 0.08 to 0.96 | 0.86 | 0.08 to 0.96 | 0.87 | 0.06 to 0.96 | 0.86 | 0.66 to 0.93 | 0.84 | 0.66 to 0.93 | 0.91 | 0.83 to 0.95 |
| LDL_DeLong_ | 0.93 | 0.22 to 0.98 | 0.91 | 0.25 to 0.97 | 0.94 | 0.15 to 0.98 | 0.94 | 0.28 to 0.98 | 0.94 | 0.37 to 0.98 | 0.94 | 0.18 to 0.98 | 0.93 | 0.92 to 0.94 | 0.93 | 0.92 to 0.94 | 0.93 | 0.91 to 0.94 |
| LDL_Rao_ | 0.68 | 0.56 to 0.76 | 0.53 | 0.47 to 0.59 | 0.91 | 0.01 to 0.98 | 0.61 | 0.53 to 0.67 | 0.83 | 0.64 to 0.9 | 0.46 | 0.40 to 0.52 | 0.90 | 0.89 to 0.91 | 0.90 | 0.89 to 0.91 | 0.91 | 0.88 to 0.92 |
| LDL_Hattori_ | 0.77 | -0.06 to 0.93 | 0.74 | -0.06 to 0.92 | 0.80 | -0.05 to 0.95 | 0.80 | -0.05 to 0.94 | 0.80 | -0.05 to 0.94 | 0.80 | -0.05 to 0.94 | 0.81 | 0.17 to 0.93 | 0.79 | 0.17 to 0.93 | 0.87 | 0.35 to 0.95 |
| LDL_Anadaraja_ | 0.85 | 0.79 to 0.89 | 0.81 | 0.58 to 0.90 | 0.88 | 0.87 to 0.89 | 0.88 | 0.85 to 0.90 | 0.87 | 0.74 to 0.92 | 0.89 | 0.88 to 0.90 | 0.85 | 0.60 to 0.93 | 0.82 | 0.60 to 0.93 | 0.91 | 0.83 to 0.95 |
| LDL_Ahmadi_ | 0.16 | 0.10 to 0.22 | 0.10 | 0.04 to 0.15 | 0.39 | 0.30 to 0.46 | 0.17 | 0.11 to 0.23 | 0.16 | 0.06 to 0.25 | 0.19 | 0.13 to 0.25 | 0.01 | -0.02 to 0.05 | 0.00 | -0.02 to 0.05 | 0.06 | -0.05 to 0.19 |
| LDL_Puavilai_ | 0.91 | 0.16 to 0.97 | 0.90 | 0.17 to 0.97 | 0.93 | 0.10 to 0.98 | 0.93 | 0.23 to 0.98 | 0.93 | 0.3 to 0.98 | 0.93 | 0.15 to 0.98 | 0.92 | 0.91 to 0.93 | 0.92 | 0.91 to 0.93 | 0.93 | 0.91 to 0.94 |
| LDL_Vujovic_ | 0.94 | 0.33 to 0.98 | 0.93 | 0.39 to 0.98 | 0.95 | 0.24 to 0.98 | 0.95 | 0.38 to 0.98 | 0.95 | 0.5 to 0.98 | 0.95 | 0.25 to 0.98 | 0.93 | 0.92 to 0.94 | 0.93 | 0.92 to 0.94 | 0.92 | 0.88 to 0.94 |
| LDL_Chen and Zhang_ | 0.89 | 0.07 to 0.97 | 0.89 | 0.23 to 0.96 | 0.89 | -0.01 to 0.97 | 0.90 | 0.07 to 0.97 | 0.92 | 0.17 to 0.98 | 0.88 | 0.01 to 0.97 | 0.93 | 0.92 to 0.94 | 0.94 | 0.92 to 0.94 | 0.91 | 0.89 to 0.93 |
| LDL_de Cordova_ | 0.74 | 0.32 to 0.88 | 0.73 | 0.55 to 0.82 | 0.76 | -0.02 to 0.92 | 0.76 | 0.32 to 0.89 | 0.80 | 0.52 to 0.9 | 0.72 | 0.13 to 0.88 | 0.78 | 0.71 to 0.82 | 0.76 | 0.71 to 0.82 | 0.81 | 0.74 to 0.85 |
| LDL_Martin_ | 0.92 | 0.15 to 0.98 | 0.92 | 0.31 to 0.97 | 0.92 | 0.04 to 0.98 | 0.93 | 0.22 to 0.98 | 0.93 | 0.44 to 0.98 | 0.92 | 0.06 to 0.98 | 0.92 | 0.90 to 0.94 | 0.93 | 0.9 to 0.94 | 0.91 | 0.85 to 0.94 |
| LDL_Choi_ | 0.98 | 0.98 to 0.98 | 0.97 | 0.97 to 0.97 | 0.99 | 0.99 to 0.99 | 0.99 | 0.99 to 0.99 | 0.98 | 0.98 to 0.99 | 0.99 | 0.99 to 0.99 | 0.87 | 0.40 to 0.95 | 0.89 | 0.4 to 0.95 | 0.85 | 0.23 to 0.94 |
| **Systemic differences** | **Mean (SD)** | **95%CI** | **Mean (SD)** | **95%CI** | **Mean (SD)** | **95%CI** | **Mean (SD)** | **95%CI** | **Mean (SD)** | **95%CI** | **Mean (SD)** | **95%CI** | **Mean (SD)** | **95%CI** | **Mean (SD)** | **95%CI** | **Mean (SD)** | **95%CI** |
| LDL_Friedwald_ | -19 (12.1) | -19.3 to -18.7 | -21 (14.4) | -21.5 to 20.4 | -17.1 (9) | -14.4 to -16.7 | -18.1 (12.5) | -18.6 to -17.6 | -19 (13.4) | -19.8 to -18.2 | -17.2 (11.5) | -17.9 to -16.6 | -12.6 (18.3) | -13.8 to -11.4 | -14.9 (19.1) | -16.5 to -13.4 | -8 (15.5) | -9.7 to 6.2 |
| LDL_DeLong_ | -12.2 (8.4) | -12.4 to -12.0 | -12.9 (9.6) | -13.3 to -12.5 | -11.5 (6.9) | -11.8 to -11.2 | -11.7 (8.2) | -12.1 to -11.4 | -11.7 (9) | -12.3 to -11.2 | -11.8 (7.4) | -12.2 to -11.3 | -0.2 (15.2) | -1.2 to 0.8 | -2 (15) | -3.2 to -0.8 | 3.4 (15.1) | 1.7 to 5.2 |
| LDL_Rao_ | -13.7 (31.9) | - 14.6 to -12.8 | -13 (44.9) | -14.8 to -11.3 | -14.4 (6.7) | -14.6 to -14.1 | -13.1 (40.9) | -14.9 to -11.4 | -13.3 (21.5) | -14.6 to -12.0 | -13 (53.3) | -16.2 to -9.8 | -0.3 (17.6) | -1.4 to 0.9 | -1.2 (17.5) | -2.6 to 2.5 | 1.5 (17.6) | -0.5 to 3.5 |
| LDL_Hattori_ | -25 (12.1) | -25.4 to -24.7 | -26.8 (14.3) | -27.3 to -26.2 | -23.3 (9.3) | -23.7 to -23.0 | -24.1 (12.5) | -24.6 to -23.5 | -24.8 (13.2) | -25.6 to -24.0 | -23.4 (11.7) | -24.1 to -22.7 | -19.6 (17.1) | -20.8 to -18.5 | -21.8 (17.8) | -23.3 to 20.4 | -15.4 (14.7) | -17.1 to -13.7 |
| LDL_Anadaraja_ | -7.6 (19.7) | -8.2 to -7.1 | -13.3 (19.8) | -14.1 to -12.6 | -2.1 (18) | -2.8 to -1.4 | -5.6 (18.8) | -6.4 to -4.8 | -10.5 (18.4) | -11.6 to -9.4 | -0.9 (18) | -2.0 to -0.1 | -13.5 (18.3) | -14.7 to -12.2 | -16.2 (19.3) | -17.7 to -14.6 | -8.1 (14.9) | -9.8 to -6.4 |
| LDL_Ahmadi_ | 42.2 (100.2) | 39.4 to 44.9 | 63 (125.2) | 58.1 to 67.8 | 21.8 (60.8) | 19.5 to 24.1 | 35.5 (100.1) | 31.3 to 39.7 | 51.3 (98.4) | 45.4 to 57.2 | 20.2 (99.3) | 14.3 to 26.1 | 145.2 (100.2) | 138.6 to 151.8 | 152.3 (109.9) | 143.4 to 161.2 | 131.1 (76.3) | 122.5 to 139.8 |
| LDL_Puavilai_ | -13.3 (8.8) | -13.5 to -13.0 | -14.2 (10.2) | -14.6 to -13.8 | -12.3 (7.1) | -12.6 to -12.1 | -12.7 (8.8) | -13.1 to -12.4 | -12.8 (9.6) | -13.4 to -12.3 | -12.6 (7.9) | -13.1 to -12.1 | -2.2 (15.6) | -3.2 to -1.1 | -4.1 (15.5) | -5.4 to -2.8 | 1.6 (15.1) | -0.1 to 3.3 |
| LDL_Vujovic_ | -10.8 (8) | -11.1 to -10.6 | -11.3 (9.2) | -11.7 to -10.9 | -10.4 (6.7) | -10.7 to -10.1 | -10.5 (7.7) | -10.8 to -10.1 | -10.2 (8.4) | -10.8 to -9.7 | -10.7 (6.9) | -11.1 to -10.3 | 2.3 (14.9) | 1.3 to 3.3 | 0.6 (14.4) | -0.6 to 1.7 | 5.7 (15.2) | 4.0 to 7.4 |
| LDL_Chen and Zhang_ | -14.9 (9.5) | -15.1 to -14.6 | -13.8 (10.9) | -14.3 to -13.4 | -15.8 (7.7) | -16.1 to -15.5 | -14.8 (9.1) | -15.1 to -14.4 | -13.5 (9) | -14.0 to -12.9 | -16 (8.9) | -16.5 to -15.5 | 1.6 (13.7) | 0.7 to 2.5 | 0.6 (12.6) | -0.4 to 1.7 | 3.5 (15.4) | 1.7 to 5.3 |
| LDL_de Cordova_ | -17.1 (20.2) | -17.6 to -16.5 | -13.2 (24.2) | -14.1 to -12.2 | -20.9 (14.3) | -21.4 to -20.3 | -17.6 (20.2) | -18.5 to -16.8 | -14.3 (19.8) | -15.5 to -13.1 | -20.9 (20.1) | -22.0 to -19.7 | 7.5 (22.1) | 6.1 to 9.0 | 8 (22.4) | 6.2 to 9.8 | 6.6 (21.5) | 4.1 to 9.0 |
| LDL_Martin_ | -12.5 (8.2) | -12.1 to -11.4 | -11.7 (9.1) | -12.1 to -11.4 | -13.3 (7) | -13.6 to -13.1 | -12.5 (8.7) | -12.9 to -12.2 | -11.2 (9.3) | -11.8 to -10.7 | -13.8 (7.8) | -14.2 to -13.3 | 4.4 (14.1) | 3.4 to 5.3 | 3.3 (13.4) | 2.2 to 4.4 | 6.5 (15.3) | 4.8 to 8.2 |
| LDL_Choi_ | 0.0 (7.6) | -0.3 to -0.2 | -0.4 (8.9) | -0.77 to 0.08 | 0.3 (6) | 0.1 to 0.6 | 0.2 (6.5) | 0.0 to 0.5 | 0.3 (7.1) | -0.1 to 0.8 | 0.1 (5.8) | -0.2 to 0.5 | 14.6 (14.3) | 13.7 to 15.6 | 13 (13.4) | 12.0 to 14.1 | 17.7 (15.5) | 15.9 to 19.5 |

The top three equations are presented in colors: red represents the highest, orange represents the second, and yellow represents the third highest intraclass correlation coefficient, or the equations with the lowest, second lowest, and third lowest mean systemic differences.

**Supplementary Table S2.** Limits of agreement and absolute error among the 12 equations in comparison with directly measured LDL

|  | **Population 1** | | | | | | | | | | | |
| --- | --- | --- | --- | --- | --- | --- | --- | --- | --- | --- | --- | --- |
|  | **Total (Test n=5198)** | | | | **Men (Test n=2570)** | | | | **Women (Test n=2628)** | | | |
| Limits of agreement | Lower | 95%CI | Upper | 95%CI | Lower | 95%CI | Upper | 95%CI | Lower | 95%CI | Upper | 95%CI |
| LDL_Friedwald_ | -42.8 | -43.3 to -42.2 | 4.8 | 4.2 to 5.3 | -49.2 | -50.2 to -48.3 | 7.3 | 6.3 to 8.2 | -34.7 | -35.3 to -34.1 | 0.5 | -0.1 to 1.1 |
| LDL_DeLong_ | -28.7 | -29.1 to -28.3 | 4.3 | 3.9 to 4.6 | -31.8 | -32.5 to -31.2 | 6.0 | 5.4 to 6.6 | -25 | -25.5 to -24.6 | 2.0 | 1.6 to 2.5 |
| LDL_Rao_ | -76.3 | -77.8 to -74.8 | 48.9 | 47.4 to 50.3 | -101 | -103.9 to -174.2 | 74.9 | 72 to 77.9 | -27.5 | -28.0 to -27.1 | -1.2 | -1.7 to -0.8 |
| LDL_Hattori_ | -48.8 | -49.4 to -48.3 | -1.3 | -1.8 to -0.7 | -54.8 | -55.7 to -53.8 | 1.2 | 0.2 to 2.1 | -41.6 | -42.2 to -40.9 | -5.1 | -5.7 to -4.5 |
| LDL_Anadaraja_ | -46.3 | -47.2 to -45.3 | 31.0 | 30.1 to 31.9 | -52.1 | -53.4 to -50.8 | 25.5 | 24.1 to 26.8 | -37.3 | -38.5 to -36.1 | 33.1 | 32.0 to 34.3 |
| LDL_Ahmadi_ | -154.3 | -159.0 to -149.6 | 238.6 | 233.9 to 243.3 | -182.5 | -190.8 to -174.2 | 308.4 | 300.1 to 316.7 | -97.4 | -101.3 to -93.4 | 141.0 | 137.0 to 144.9 |
| LDL_Puavilai_ | -30.6 | -31.0 to -30.1 | 4.0 | 3.6 to 4.5 | -34.2 | -34.8 to -33.5 | 5.8 | 5.1 to 6.5 | -26.3 | -26.8 to -25.9 | 1.7 | 1.2 to 2.1 |
| LDL_Vujovic_ | -26.6 | -26.9 to -262 | 4.9 | 4.5 to 5.2 | -29.3 | -29.9 to-28.7 | 6.7 | 6.1 to 7.3 | -23.5 | -23.9 to -23.0 | 2.6 | 2.2 to 3.1 |
| LDL_Chen and Zhang_ | -33.4 | -33.9 to -33.0 | 3.7 | 3.3 to 4.2 | -35.3 | -36.0 to -34.6 | 7.6 | 6.9 to 8.3 | -30.9 | -31.4 to -30.4 | -0.8 | -1.3 to -0.3 |
| LDL_de Cordova_ | -56.6 | -57.6 to -55.7 | 22.5 | 21.6 to 23.4 | -60.6 | -62.2 to -59.0 | 34.3 | 32.7 to 35.9 | -48.8 | -49.7 to -47.9 | 7.1 | 6.1 to 8.0 |
| LDL_Martin_ | -28.5 | -28.9 to -28.1 | 3.4 | 3.1 to 3.8 | -29.6 | -30.2 to -29.0 | 6.1 | 5.5 to 6.7 | -27 | -27.5 to -26.6 | 0.4 | -0.1 to 0.8 |
| LDL_Choi_ | -14.9 | -15.3 to -14.6 | 14.8 | 14.5 to 15.2 | -17.9 | -18.5 to -17.3 | 17.1 | 16.5 to 17.7 | -11.4 | -11.8 to -11.0 | 12.1 | 11.7 to 12.5 |
| Absolute percentage error | Med | 95% CI | 95th P | 95% CI | Med | 95% CI | 95th P | 95% CI | Med | 95% CI | 95th P | 95% CI |
| LDL_Friedwald_ | 15.5 | 15.3 to 15.7 | 39.7 | 38.1 to 41.2 | 16.8 | 16.3 to 17.3 | 47.5 | 43.8 to 52.6 | 14.3 | 14 to 14.6 | 30.9 | 29.2 to 32.6 |
| LDL_DeLong_ | 10.6 | 10.3 to 10.8 | 24.8 | 24.0 to 26 | 11.3 | 10.9 to 11.5 | 29.7 | 28.1 to 32.0 | 10 | 9.8 to 10.3 | 21.3 | 20.5 to 22.2 |
| LDL_Rao_ | 13.3 | 13.1 to 13.6 | 27.1 | 26.5 to 27.9 | 14.0 | 13.7 to 14.3 | 30.3 | 29.1 to 32.6 | 12.8 | 12.5 to 13.1 | 24.3 | 23.8 to 25.5 |
| LDL_Hattori_ | 20.8 | 20.6 to 21.1 | 43.7 | 42.2 to 45.4 | 22.0 | 21.6 to 22.5 | 51.2 | 47.9 to 56.2 | 19.7 | 19.4 to 20.0 | 35.5 | 33.9 to 37.3 |
| LDL_Anadaraja_ | 11.4 | 11.0 to 11.8 | 41.1 | 39.5 to 43 | 12.9 | 12.3 to 13.5 | 46.2 | 43.3 to 49.1 | 10.2 | 9.8 to 10.5 | 35.0 | 33.3 to 38.1 |
| LDL_Ahmadi_ | 25.4 | 24.3 to 26.4 | 192.9 | 182.1 to 206.5 | 32.1 | 30.3 to 34.6 | 254.6 | 221.8 to 286.7 | 21.5 | 20.4 to 22.4 | 128.1 | 115.6 to 143.7 |
| LDL_Puavilai_ | 11.4 | 11.1 to 11.5 | 26.9 | 25.8 to 28.2 | 12.1 | 11.8 to 12.4 | 32.3 | 30.6 to 34.8 | 10.7 | 10.5 to 11.0 | 22.5 | 21.9 to 23.6 |
| LDL_Vujovic_ | 9.5 | 9.3 to 9.7 | 22.7 | 21.8 to 23.4 | 10.0 | 9.8 to 10.3 | 26.6 | 25 to 28.3 | 9.1 | 8.9 to 9.3 | 20.0 | 19.1 to 20.5 |
| LDL_Chen and Zhang_ | 13.6 | 13.4 to 13.7 | 22.2 | 21.9 to 22.6 | 13.3 | 13.0 to 13.6 | 22.6 | 21.9 to 23.2 | 13.8 | 13.6 to 14.0 | 22 | 21.5 to 22.5 |
| LDL_de Cordova_ | 18.2 | 17.9 to 18.4 | 29.6 | 29.2 to 30.2 | 16.5 | 16.1 to 17.0 | 30.4 | 29.1 to 32.3 | 19.3 | 19 to 19.6 | 29.4 | 29.0 to 30.0 |
| LDL_Martin_ | 11.5 | 11.4 to 11.7 | 22.0 | 21.5 to 22.8 | 11.4 | 11.1 to 11.6 | 22.2 | 21.4 to 23.4 | 11.7 | 11.5 to 11.9 | 21.9 | 21.2 to 22.9 |
| LDL_Choi_ | 3.3 | 3.2 to 3.5 | 13.1 | 12.3 to 13.9 | 3.5 | 3.4 to 3.7 | 14.8 | 14.0 to 16.7 | 3.1 | 2.9 to 3.3 | 11.0 | 10.2 to 11.7 |

*(Continue)*

**Supplementary Table S2.** (*Continue*)

|  | **Population 2** | | | | | | | | | | | |
| --- | --- | --- | --- | --- | --- | --- | --- | --- | --- | --- | --- | --- |
|  | **Total (Test n=2163)** | | | | **Men (Test n=1062)** | | | | **Women (Test n=1101)** | | | |
| Limits of agreement | Lower | 95%CI | Upper | 95%CI | Lower | 95%CI | Upper | 95%CI | Lower | 95%CI | Upper | 95%CI |
| LDL_Friedwald_ | -42.5 | -43.4 to -41.6 | 6.3 | 5.4 to 7.2 | -45.2 | -46.6 to -43.8 | 7.2 | 5.8 to 8.6 | -39.7 | -40.9 to -38.6 | 5.3 | 4.1 to 6.4 |
| LDL_DeLong_ | -27.9 | -285 to -27.3 | 4.4 | 3.8 to 5 | -29.4 | -30.3 to -28.5 | 6.0 | 5.0 to 6.9 | -26.2 | -27.0 to -25.5 | 2.7 | 1.9 to 3.4 |
| LDL_Rao_ | -93.3 | -96.2 to -90.3 | 67.0 | 64.1 to 69.9 | -55.4 | -57.7 to -53.2 | 28.8 | 26.6 to 31.1 | -117.5 | -122.8 to -112.1 | 91.5 | 86.1 to 96.8 |
| LDL_Hattori_ | -48.5 | -49.4 to -47.6 | 0.4 | -0.5 to 1.3 | -50.7 | -52.1 to -49.3 | 1.1 | -0.3 to 2.4 | -46.3 | -47.5 to -45.1 | -0.4 | -1.6 to 0.7 |
| LDL_Anadaraja_ | -42.4 | -43.7 to -41.0 | 31.1 | 29.8 to 32.5 | -46.4 | -48.3 to -44.6 | 25.5 | 23.6 to 27.4 | -36.1 | -37.9 to -34.3 | 34.2 | 32.4 to 36.1 |
| LDL_Ahmadi_ | -160.6 | -167.9 to -153.4 | 231.6 | 224.4 to 238.8 | -141.5 | -151.7 to -131.4 | 244.2 | 195.5 to 285.4 | -174.5 | -184.5 to -164.4 | 214.9 | 204.8 to 224.9 |
| LDL_Puavilai_ | -29.9 | -30.6 to -29.3 | 4.5 | 3.9 to 5.1 | -31.7 | -32.7 to -30.7 | 6.0 | 5 to 7 | -28.1 | -28.9 to -27.3 | 2.9 | 2.1 to 3.7 |
| LDL_Vujovic_ | -25.5 | -26.1 to -25.0 | 4.6 | 4.1 to 5.2 | -26.8 | -27.6 to -25.9 | 6.3 | 5.4 to 7.2 | -24.2 | -24.9 to -23.5 | 2.9 | 2.2 to 3.6 |
| LDL_Chen and Zhang_ | -32.5 | -33.2 to -31.9 | 3.0 | 2.3 to 3.7 | -31.2 | -32.1 to -30.3 | 4.2 | 3.3 to 5.2 | -33.5 | -34.4 to -32.6 | 1.5 | 0.6 to 2.4 |
| LDL_de Cordova_ | -57.2 | -58.7 to -55.8 | 22.0 | 20.5 to 23.4 | -53.1 | -55.1 to -51.0 | 24.5 | 22.5 to 26.6 | -60.2 | -62.2 to -58.2 | 18.5 | 16.5 to 20.5 |
| LDL_Martin_ | -29.6 | -30.2 to -28.9 | 4.5 | 3.9 to 5.1 | -29.5 | -30.5 to -28.6 | 7.0 | 6.1 to 8 | -29.1 | -29.9 to -28.3 | 1.6 | 0.8 to 2.3 |
| LDL_Choi_ | -12.5 | -12.9 to -12.0 | 12.9 | 12.5 to 13.4 | -13.5 | -14.3 to -12.8 | 14.2 | 13.5 to 14.9 | -11.3 | -11.9 to -10.7 | 11.6 | 11 to 12.2 |
| Absolute percentage error | Med | 95% CI | 95th P | 95% CI | Med | 95% CI | 95th P | 95% CI | Med | 95% CI | 95th P | 95% CI |
| LDL_Friedwald_ | 15.1 | 14.7 to 15.5 | 35.4 | 33.7 to 37.5 | 15.7 | 15.1 to 16.2 | 42.4 | 37.3 to 48.5 | 14.6 | 14.2 to 15 | 30.2 | 27.8 to 31.9 |
| LDL_DeLong_ | 10.3 | 10 to 10.6 | 23.5 | 22.4 to 24.5 | 10.2 | 9.9 to 0.7 | 25.6 | 24.2 to 28.1 | 10.4 | 9.9 to 10.7 | 21.2 | 20.5 to 22.4 |
| LDL_Rao_ | 13.3 | 12.9 to 13.5 | 27.1 | 25.9 to 28.1 | 13.3 | 12.9 to 13.7 | 28.7 | 27.4 to 31.4 | 13.3 | 12.7 to 13.6 | 25.0 | 23.9 to 26.4 |
| LDL_Hattori_ | 20.4 | 20.1 to 20.8 | 39.8 | 38.2 to 42 | 21.0 | 20.5 to 21.6 | 47.2 | 42.1 to 52.9 | 20.0 | 19.6 to 20.2 | 34.8 | 32.9 to 37 |
| LDL_Anadaraja_ | 10.4 | 9.9 to 11 | 36.9 | 35 to 39.8 | 11.9 | 11.1 to 12.7 | 39.5 | 36.3 to 44 | 9.2 | 8.5 to 9.9 | 34.8 | 31.1 to 38.3 |
| LDL_Ahmadi_ | 26.8 | 25.3 to 28.1 | 177.7 | 159.6 to 191.6 | 31.8 | 29.5 to 34.7 | 229.0 | 195.5 to 285.4 | 23.2 | 21.5 to 24.8 | 117.0 | 106 to 139.9 |
| LDL_Puavilai_ | 11.1 | 10.8 to 11.3 | 25.0 | 24 to 26.2 | 11.1 | 10.7 to 11.5 | 28.1 | 25.7 to 31 | 11.0 | 10.6 to 11.5 | 22.6 | 21.4 to 23.7 |
| LDL_Vujovic_ | 9.3 | 9.1 to 9.6 | 21.5 | 21 to 22.2 | 9.3 | 8.9 to 9.6 | 23.0 | 22 to 24.4 | 9.4 | 9.1 to 9.7 | 20.3 | 18.8 to 21.1 |
| LDL_Chen and Zhang_ | 13.8 | 13.5 to 14.1 | 22.7 | 22.1 to 23.5 | 13.0 | 12.5 to 13.5 | 22.6 | 21.5 to 23.7 | 14.5 | 14 to 14.8 | 22.8 | 22.1 to 24 |
| LDL_de Cordova_ | 18.5 | 18.1 to 19 | 30.6 | 30 to 31.5 | 17.0 | 16.4 to 17.7 | 31.2 | 29.3 to 34.2 | 20.0 | 19.4 to 20.7 | 30.5 | 29.9 to 31.3 |
| LDL_Martin_ | 12.0 | 11.7 to 12.2 | 23.8 | 23 to 24.5 | 11.4 | 10.8 to 11.8 | 24.0 | 23 to 25.3 | 12.4 | 12.1 to 12.9 | 23.7 | 21.9 to 24.5 |
| LDL_Choi_ | 3.4 | 3.2 to 3.6 | 12.3 | 11.4 to 14.2 | 3.5 | 3.2 to 3.7 | 14.6 | 12.1 to 17 | 3.3 | 3.1 to 3.5 | 11.1 | 10.4 to 12.4 |

*(Continue)*

|  | **Population 3** | | | | | | | | | | | |
| --- | --- | --- | --- | --- | --- | --- | --- | --- | --- | --- | --- | --- |
|  | **Total (Test n=889)** | | | | **Men (Test n=588)** | | | | **Women (Test n=301)** | | | |
| **Limits of agreement** | **Lower** | **95%CI** | **Upper** | **95%CI** | **Lower** | **95%CI** | **Upper** | **95%CI** | **Lower** | **95%CI** | **Upper** | **95%CI** |
| LDL_Friedwald_ | -48.4 | -50.5 to -43.3 | 23.2 | 21.2 to 25.3 | -52.4 | -55.1 to -49.8 | 22.5 | 19.9 to 25.2 | -38.4 | -41.4 to -35.4 | 22.5 | 19.4 to 25.5 |
| LDL_DeLong_ | -30.0 | -31.7 to -28.3 | 29.7 | 28 to 31.4 | -31.3 | -33.4 to -29.2 | 27.3 | 25.2 to 29.4 | -26.2 | -29.2 to -23.3 | 33.1 | 30.2 to 36 |
| LDL_Rao_ | -34.7 | -36.7 to -32.8 | 34.2 | 32.2 to 36.2 | -35.5 | -38.0 to -33.1 | 33.2 | 30.8 to 35.6 | -33.0 | -36.4 to -29.6 | 36.0 | 32.6 to 39.4 |
| LDL_Hattori_ | -53.2 | -55.1 to -51.2 | 13.9 | 11.9 to 15.8 | -56.8 | -59.2 to -54.3 | 13.2 | 10.7 to 15.6 | -44.2 | -47.0 to -41.3 | 13.4 | 10.5 to 16.2 |
| LDL_Anadaraja_ | -49.4 | -51.4 to -47.3 | 22.5 | 20.4 to 24.5 | -54.0 | -56.7 to -51.3 | 21.7 | 19 to 24.3 | -37.3 | -40.2 to -34.4 | 21.1 | 18.2 to 24 |
| LDL_Ahmadi_ | -51.3 | -62.6 to -40.0 | 341.6 | 330.3 to 352.9 | -63.0 | -78.2 to -47.8 | 367.7 | 352.5 to 382.9 | -18.4 | -33.2 to -3.6 | 280.6 | 265.8 to 295.4 |
| LDL_Puavilai_ | -32.8 | -34.6 to -31.0 | 28.5 | 26.7 to 30.2 | -34.5 | -36.7 to -32.4 | 26.4 | 24.2 to 28.5 | -28.0 | -31.0 to -25.1 | 31.3 | 28.4 to 34.2 |
| LDL_Vujovic_ | -26.9 | -28.5 to -25.2 | 31.5 | 29.8 to 33.1 | -27.7 | -29.7 to -25.7 | 28.8 | 26.8 to 30.8 | -24.1 | -27.1 to -21.2 | 35.5 | 32.6 to 38.5 |
| LDL_Chen and Zhang_ | -25.3 | -26.8 to -23.7 | 28.5 | 26.9 to 30 | -24.1 | -25.9 to -22.4 | 25.4 | 23.7 to 27.2 | -26.8 | -29.8 to -23.8 | 33.8 | 30.8 to 36.8 |
| LDL_de Cordova_ | -35.8 | -38.3 to -33.3 | 50.8 | 48.3 to 53.3 | -35.8 | -38.9 to -32.7 | 51.9 | 48.8 to 55 | -35.7 | -39.8 to -31.5 | 48.8 | 44.6 to 53 |
| LDL_Martin_ | -23.3 | -24.9 to -21.7 | 32.0 | 30.4 to 33.6 | -22.9 | -24.8 to -21.1 | 29.5 | 27.6 to 31.3 | -23.4 | -26.3 to -20.4 | 36.4 | 33.4 to 39.4 |
| LDL_Choi_ | -13.4 | -15.0 to -11.8 | 42.6 | 41 to 44.2 | -13.2 | -15.0 to -11.3 | 39.3 | 37.4 to 41.1 | -12.6 | -15.6 to -9.6 | 48.0 | 45 to 51 |
| **Absolute percentage error** | **Med** | **95% CI** | **95th P** | **95% CI** | **Med** | **95% CI** | **95th P** | **95% CI** | **Med** | **95% CI** | **95th P** | **95% CI** |
| LDL_Friedwald_ | 10.1 | 9.3 to 10.6 | 51.7 | 46.9 to 73 | 10.9 | 10.3 to 11.7 | 70.2 | 49.5 to 86.7 | 7.7 | 6.9 to 9.0 | 34.6 | 27.7 to 50.2 |
| LDL_DeLong_ | 6.1 | 5.5 to 6.6 | 34.4 | 30.8 to 46.2 | 6.2 | 5.5 to 6.8 | 43.9 | 32.5 to 51.5 | 5.9 | 5.2 to 6.6 | 29.9 | 22.4 to 33.5 |
| LDL_Rao_ | 6.6 | 6.2 to 7.0 | 30.3 | 28.4 to 37.7 | 6.7 | 6.1 to 7.3 | 32.2 | 29.1 to 49.1 | 6.3 | 5.5 to 7.0 | 28.6 | 22.2 to 32.4 |
| LDL_Hattori_ | 15.4 | 14.7 to 5.9 | 56.0 | 51 to 76.4 | 16.3 | 15.6 to 7.3 | 68.8 | 54.2 to 86.7 | 13.0 | 11.8 to 3.9 | 39.8 | 32.3 to 54.3 |
| LDL_Anadaraja_ | 10.6 | 9.9 to 11.8 | 54.1 | 47 to 64.7 | 12.6 | 11.3 to 3.9 | 61.0 | 51 to 77.8 | 8.6 | 7.6 to 9.5 | 35.7 | 28.3 to 45.7 |
| LDL_Ahmadi_ | 99.1 | 94.1 to 3.2 | 443.0 | 394.2 to 501.5 | 102.3 | 97.1 to 107.4 | 474.9 | 427.6 to 578.2 | 92.9 | 87.0 to 100.8 | 315.0 | 274.6 to 429.7 |
| LDL_Puavilai_ | 6.1 | 5.7 to 6.8 | 36.7 | 30.9 to 44.5 | 6.4 | 5.8 to 7.2 | 43.3 | 34.5 to 58.8 | 5.7 | 5.2 to 6.7 | 29.3 | 24.2 to 35.5 |
| LDL_Vujovic_ | 6.0 | 5.6 to 6.7 | 34.9 | 28.2 to 41.7 | 5.8 | 5.1 to 6.4 | 40.1 | 29.3 to 51.7 | 6.6 | 5.7 to 7.4 | 28.7 | 23.1 to 35.1 |
| LDL_Chen and Zhang_ | 5.8 | 5.3 to 6.1 | 28.6 | 24.3 to 35.8 | 5.8 | 5.1 to 6.4 | 28.6 | 22.7 to 36 | 5.7 | 5.0 to 6.7 | 28.9 | 23.1 to 42.1 |
| LDL_de Cordova_ | 7.9 | 7.1 to 8.6 | 68.3 | 60 to 75.3 | 7.8 | 6.8 to 8.6 | 70.6 | 64.8 to 98.7 | 8.2 | 7.0 to 9.9 | 55.1 | 38.2 to 70.3 |
| LDL_Martin_ | 5.6 | 5.2 to 6.1 | 36.6 | 30.8 to 47.5 | 5.5 | 4.9 to 6.0 | 39.8 | 30.3 to 48.3 | 6.1 | 5.1 to 6.9 | 34.5 | 27.1 to 49.7 |
| LDL_Choi_ | 12.1 | 11.5 to 12.8 | 40.7 | 35 to 48.3 | 11.1 | 9.8 to 12.0 | 40.8 | 34.4 to 48.5 | 13.9 | 12.7 to 5.1 | 39.9 | 32.2 to 49.8 |

The top three equations are presented in color: red represents the lowest, orange represents the second, and yellow represents the third lowest absolute percentage error.

**Supplementary Table S3**. Intraclass correlation coefficient and systemic differences among the 12 equations in comparison with directly measured LDL (LDL_direct_) by subgroup of LDL concentration

|  | **Population 1** | | | | | | | | | | | |
| --- | --- | --- | --- | --- | --- | --- | --- | --- | --- | --- | --- | --- |
|  | **Total** | | | | **Men** | | | | **Women** | | | |
|  | **LDL group 1**  **(LDL <70 mg/dL,**  **Test n=581)** | | **LDL group 2**  **(LDL 70 mg/dL,**  **Test n=4617)** | | **LDL group 1**  **(LDL <70 mg/dL,**  **Test n=357)** | | **LDL group 2**  **(LDL 70 mg/dL,**  **Test n=2213)** | | **LDL group 1**  **(LDL <70 mg/dL,**  **Test n=224)** | | **LDL group 2**  **(LDL 70 mg/dL,**  **Test n=2404)** | |
| **Intraclass correlation** | **Coefficient** | **95%CI** | **Coefficient** | **95%CI** | **Coefficient** | **95%CI** | **Coefficient** | **95%CI** | **Coefficient** | **95%CI** | **Coefficient** | **95%CI** |
| LDL_Friedwald_ | 0.28 | -0.05 to 0.52 | 0.81 | -0.03 to 0.94 | 0.25 | -0.04 to 0.48 | 0.76 | -0.04 to 0.92 | 0.37 | -0.1 to 0.69 | 0.85 | -0.03 to 0.96 |
| LDL_DeLong_ | 0.49 | 0.08 to 0.70 | 0.91 | 0.11 to 0.97 | 0.45 | 0.1 to 0.66 | 0.89 | 0.1 to 0.97 | 0.59 | -0.03 to 0.82 | 0.92 | 0.09 to 9790 |
| LDL_Rao_ | 0.00 | -0.08 to 0.08 | 0.87 | 0.06 to 0.96 | -0.01 | -0.11 to 0.09 | 0.84 | 0.17 to 0.94 | 0.47 | -0.08 to 0.75 | 0.90 | -0.02 to 0.97 |
| LDL_Hattori_ | 0.24 | -0.08 to 0.5 | 0.72 | -0.06 to 0.92 | 0.22 | -0.07 to 0.47 | 0.67 | -0.07 to 0.89 | 0.30 | -0.08 to 0.64 | 0.76 | -0.05 to 0.93 |
| LDL_Anadaraja_ | 0.30 | 0.22 to 0.37 | 0.81 | 0.73 to 0.87 | 0.28 | 0.17 to 0.37 | 0.77 | 0.43 to 0.88 | 0.33 | 0.21 to 0.44 | 0.86 | 0.84 to 685 |
| LDL_Ahmadi_ | -0.01 | -0.08 to 0.06 | 0.21 | 0.12 to 0.29 | -0.01 | -0.1 to 0.08 | 0.14 | 0.04 to 0.22 | 0.02 | -0.08 to 0.14 | 0.37 | 0.28 to 365 |
| LDL_Puavilai_ | 0.46 | 0.02 to 0.69 | 0.89 | 0.07 to 0.97 | 0.42 | 0.03 to 0.65 | 0.87 | 0.06 to 0.96 | 0.55 | -0.07 to 0.81 | 0.91 | 0.05 to 9766 |
| LDL_Vujovic_ | 0.51 | 0.19 to 0.69 | 0.92 | 0.18 to 0.98 | 0.47 | 0.21 to 0.64 | 0.91 | 0.19 to 0.97 | 0.62 | 0.05 to 0.83 | 0.93 | 0.16 to 817 |
| LDL_Chen and Zhang_ | 0.34 | 0.24 to 0.43 | 0.86 | -0.03 to 0.96 | 0.26 | 0.17 to 0.36 | 0.86 | -0.02 to 0.96 | 0.58 | 0.09 to 0.79 | 0.86 | -0.03 to 0.96 |
| LDL_de Cordova_ | 0.04 | -0.04 to 0.12 | 0.73 | 0.03 to 0.9 | 0.01 | -0.09 to 0.11 | 0.74 | 0.24 to 0.88 | 0.38 | 0.25 to 0.49 | 0.72 | -0.06 to 0.91 |
| LDL_Martin_ | 0.50 | 0.31 to 0.64 | 0.90 | 0.02 to 0.97 | 0.48 | 0.35 to 0.59 | 0.90 | 0.07 to 0.97 | 0.55 | 0.07 to 0.77 | 0.90 | 0 to 0.97 |
| LDL_Choi_ | 0.49 | 0.41 to 0.56 | 0.98 | 0.98 to 0.98 | 0.41 | 0.31 to 0.49 | 0.97 | 0.97 to 0.98 | 0.74 | 0.59 to 0.83 | 0.99 | 0.98 to 862 |
| **Systemic differences** | **Mean (SD)** | **95%CI** | **Mean (SD)** | **95%CI** | **Mean (SD)** | **95%CI** | **Mean (SD)** | **95%CI** | **Mean (SD)** | **95%CI** | **Mean (SD)** | **95%CI** |
| LDL_Friedwald_ | -16.9 (15.9) | -18.2 to -15.6 | -19.3 (11.5) | -19.6 to -18.9 | -19 (18.9) | -21.0 to -17.0 | -21.3 (13.5) | -21.8 to -20.7 | -13.6 (8.3) | -14.7 to -12.4 | -17.4 (9.0) | -17.8 to -17 |
| LDL_DeLong_ | -9.1 (10.4) | -9.9 to -8.2 | -12.6 (8.0) | -12.8 to -12.3 | -9.7 (12.1) | -11.0 to -8.5 | -13.4 (9.1) | -13.8 to -13 | -8 (6.7) | -8.9 to -7.1 | -11.8 (6.8) | -12.1 to -11.5 |
| LDL_Rao_ | -4.4 (90.9) | -11.8 to 3.0 | -14.9 (9.8) | -15.2 to -14.5 | -0.5 (115.7) | -12.5 to 11.6 | -15 (12.4) | -15.6 to -14.5 | -10.6 (8.1) | -11.7 to -9.5 | -14.7 (6.5) | -15 to -14.4 |
| LDL_Hattori_ | -19.7 (15.4) | -21 to -18.4 | -25.7 (11.5) | -26 to -25.3 | -21.7 (18.3) | -23.6 to -19.8 | -27.6 (13.3) | -28.2 to -27 | -16.5 (8.0) | -17.5 to -15.4 | -24 (9.1) | -24.3 to -23.6 |
| LDL_Anadaraja_ | -3.0 (21.9) | -4.8 to -1.2 | -8.2 (19.3) | -8.8 to -7.7 | -6.7 (23.6) | -9.1 to -4.2 | -14.4 (18.9) | -15.2 to -13.6 | 2.8 (17.5) | 0.5 to 5.1 | -2.5 (18.0) | -3.3 to -1.8 |
| LDL_Ahmadi_ | 70.8 (188.3) | 55.5 to 86.2 | 38.5 (82.1) | 36.2 to 40.9 | 94.1 (230.6) | 70.1 to 118.1 | 57.9 (97.4) | 53.9 to 62 | 33.7 (71.3) | 24.3 to 43.1 | 20.7 (59.6) | 18.3 to 23.1 |
| LDL_Puavilai_ | -10.3 (10.7) | -11.2 to -9.4 | -13.6 (8.5) | -13.9 to -13.3 | -11.2 (12.5) | -12.5 to -9.9 | -14.7 (9.7) | -15.1 to -14.2 | -8.8 (6.8) | -9.7 to -8.0 | -12.7 (7.1) | -13 to -12.3 |
| LDL_Vujovic_ | -7.5 (10.5) | -8.4 to -6.7 | -11.3 (7.5) | -11.5 to -11.0 | -7.9 (12.3) | -9.2 to -6.6 | -11.8 (8.4) | -12.2 to -11.4 | -7.0 (6.7) | -7.8 to -6.1 | -10.7 (6.6) | -11 to -10.4 |
| LDL_Chen and Zhang_ | -5.2 (15.3) | -6.5 to -4.0 | -16.1 (7.7) | -16.3 to -15.8 | -4.2 (18.5) | -6.2 to -2.3 | -15.4 (8.1) | -15.7 to -15 | -6.9 (7.4) | -7.8 to -5.9 | -16.7 (7.2) | -17 to -16.3 |
| LDL_de Cordova_ | 2.5 (35.0) | -0.3 to 5.4 | -19.5 (15.8) | -20.0 to -19.0 | 6.4 (42.9) | 1.9 to 10.9 | -16.3 (17.7) | -17 to -15.5 | -3.7 (13.6) | -5.5 to -1.9 | -22.5 (13.2) | -23 to -21.9 |
| LDL_Martin_ | -5.9 (11.0) | -6.8 to -5.0 | -13.4 (7.3) | -13.6 to -13.1 | -4.9 (12.4) | -6.2 to -3.6 | -12.8 (7.9) | -13.2 to -12.5 | -7.6 (8.0) | -8.6 to -6.5 | -13.9 (6.6) | -14.1 to -13.5 |
| LDL_Choi_ | 3.4 (12.6) | 2.4 to 4.4 | -0.5 (6.6) | -0.7 to -0.3 | 3.6 (15.2) | 2.0 to 5.1 | -1.1 (7.2) | -1.4 to -0.8 | 3.2 (6.7) | 2.3 to 0 | 0.1 (5.9) | -0.2 to 0.3 |

(*continue*)

**Supplementary Table S3**. (*continue*)

|  | **Population 2** | | | | | | | | | | | |
| --- | --- | --- | --- | --- | --- | --- | --- | --- | --- | --- | --- | --- |
|  | **Total** | | | | **Men** | | | | **Women** | | | |
|  | **LDL group 1 (LDL <70 mg/dL, Test n=287)** | | **LDL group 2**  **(LDL 70 mg/dL, Test n=1876)** | | **LDL group 1 (LDL <70 mg/dL, Test n=164)** | | **LDL group 2 (LDL 70 mg/dL, Test n=898)** | | **LDL group 1**  **(LDL <70 mg/dL,**  **Test n=123)** | | **LDL group 2**  **(LDL 70 mg/dL,**  **Test n=978)** | |
| **Intraclass correlation** | **Coefficient** | **95%CI** | **Coefficient** | **95%CI** | **Coefficient** | **95%CI** | **Coefficient** | **95%CI** | **Coefficient** | **95%CI** | **Coefficient** | **95%CI** |
| LDL_Friedwald_ | 0.23 | 0.04 to 0.39 | 0.85 | -0.03 to 0.96 | 0.25 | 0.02 to 0.44 | 0.85 | -0.03 to 0.96 | 0.18 | 0.01 to 0.35 | 0.86 | -0.03 to 0.96 |
| LDL_DeLong_ | 0.44 | 0.14 to 0.63 | 0.93 | 0.09 to 0.98 | 0.44 | 0.14 to 0.63 | 0.93 | 0.12 to 0.98 | 0.44 | 0.08 to 0.66 | 0.92 | 0.06 to 0.98 |
| LDL_Rao_ | 0.00 | -0.12 to 0.11 | 0.90 | -0.01 to 0.97 | 0.04 | -0.11 to 0.19 | 0.90 | 0 to 0.97 | -0.01 | -0.19 to 0.17 | 0.89 | -0.02 to 0.97 |
| LDL_Hattori_ | 0.21 | 0.00 to 0.39 | 0.76 | -0.04 to 0.93 | 0.23 | -0.02 to 0.44 | 0.76 | -0.05 to 0.93 | 0.17 | -0.02 to 0.34 | 0.77 | -0.04 to 0.94 |
| LDL_Anadaraja_ | 0.25 | 0.13 to 0.35 | 0.87 | 0.82 to 0.91 | 0.27 | 0.12 to 0.4 | 0.86 | 0.61 to 0.93 | 0.19 | 0.01 to 0.35 | 0.89 | 0.87 to 0.9 |
| LDL_Ahmadi_ | -0.01 | -0.12 to 0.09 | 0.29 | 0.18 to 0.39 | -0.02 | -0.14 to 0.11 | 0.24 | 0.07 to 0.37 | -0.01 | -0.18 to 0.17 | 0.38 | 0.3 to 0.45 |
| LDL_Puavilai_ | 0.39 | 0.11 to 0.58 | 0.92 | 0.05 to 0.98 | 0.39 | 0.11 to 0.59 | 0.92 | 0.07 to 0.98 | 0.37 | 0.07 to 0.59 | 0.91 | 0.03 to 0.98 |
| LDL_Vujovic_ | 0.51 | 0.19 to 0.69 | 0.94 | 0.18 to 0.98 | 0.49 | 0.21 to 0.67 | 0.94 | 0.24 to 0.98 | 0.54 | 0.08 to 0.76 | 0.93 | 0.13 to 0.98 |
| LDL_Chen and Zhang_ | 0.55 | 0.25 to 0.72 | 0.87 | -0.02 to 0.96 | 0.61 | 0.33 to 0.76 | 0.89 | 0.01 to 0.97 | 0.44 | 0.13 to 0.64 | 0.85 | -0.03 to 0.96 |
| LDL_de Cordova_ | 0.02 | -0.09 to 0.14 | 0.73 | 0.00 to 0.90 | 0.05 | -0.1 to 0.2 | 0.77 | 0.15 to 0.91 | 0.01 | -0.17 to 0.19 | 0.70 | -0.06 to 0.9 |
| LDL_Martin_ | 0.51 | 0.22 to 0.68 | 0.90 | 0.06 to 0.97 | 0.50 | 0.3 to 0.64 | 0.92 | 0.16 to 0.98 | 0.52 | 0.00 to 0.76 | 0.89 | 0 to 0.97 |
| LDL_Choi_ | 0.72 | 0.65 to 0.78 | 0.98 | 0.98 to 0.99 | 0.69 | 0.6 to 0.76 | 0.98 | 0.98 to 0.99 | 0.81 | 0.57 to 0.9 | 0.99 | 0.98 to 0.99 |
| **Systemic differences** | **Mean (SD)** | **95%CI** | **Mean (SD)** | **95%CI** | **Mean (SD)** | **95%CI** | **Mean (SD)** | **95%CI** | **Mean (SD)** | **95%CI** | **Mean (SD)** | **95%CI** |
| LDL_Friedwald_ | -17.7 (26.0) | -20.7 to -14.6 | -18.2 (8.7) | -18.6 to -17.7 | -19.3 (26.0) | -23.3 to -15.2 | -18.9 (9.4) | -19.6 to -18.3 | -15.5 (26) | -20.1 to -10.8 | -17.5 (8) | -18 to -16.9 |
| LDL_DeLong_ | -10.1 (14.1) | -11.7 to -8.5 | -12 (6.9) | -12.3 to -11.6 | -10.9 (15.9) | -13.3 to -8.4 | -11.9 (7.1) | -12.3 to -11.4 | -9.1 (11.4) | -11.1 to -7.1 | -12.1 (6.6) | -12.5 to -11.6 |
| LDL_Rao_ | -2.0 (110.4) | -14.8 to 10.9 | -14.9 (6.8) | -15.2 to -14.5 | -5.8 (51.6) | -13.8 to 2.1 | -14.7 (7.2) | -15.1 to -14.1 | 3.2 (158.1) | -25 to 31.4 | -15 (6.4) | -15.4 to -14.6 |
| LDL_Hattori_ | -20.3 (25.0) | -23.2 to -17.4 | -24.7 (9) | -25.1 to -24.2 | -21.8 (24.9) | -25.6 to -17.9 | -25.4 (9.6) | -26.0 to -24.7 | -18.3 (25.1) | -22.8 to -13.8 | -24 (8.5) | -24.5 to -23.4 |
| LDL_Anadaraja_ | -1.4 (29.3) | -4.8 to 2.0 | -6.3 (16.5) | -7.0 to -5.5 | -6.1 (29.2) | -10.6 to -1.6 | -11.3 (15.5) | -12.3 to -10.2 | 4.8 (28.3) | -0.3 to 9.8 | -1.7 (16.1) | -2.7 to -0.7 |
| LDL_Ahmadi_ | 65.5 (212.8) | 40.7 to 90.2 | 30.9 (66.9) | 27.9 to 33.9 | 79.7 (181.9) | 51.6 to 107.7 | 46.1 (72.6) | 41.4 to 50.9 | 46.5 (247.7) | 2.3 to 90.7 | 16.9 (57.9) | 13.3 to 20.5 |
| LDL_Puavilai_ | -11.3 (16) | -13.2 to -9.4 | -12.9 (7) | -13.3 to -12.6 | -12.2 (17.5) | -14.9 to -9.5 | -13 (7.4) | -13.4 to -12.4 | -10.1 (13.7) | -12.6 to -7.7 | -12.9 (6.8) | -13.3 to -12.5 |
| LDL_Vujovic_ | -8.6 (12.1) | -10 to -7.2 | -10.7 (6.7) | -11.1 to -10.4 | -9.2 (14.1) | -11.3 to -7 | -10.4 (6.9) | -10.9 to -10.0 | -7.8 (8.7) | -9.4 to -6.3 | -11 (6.6) | -11.4 to -10.6 |
| LDL_Chen and Zhang_ | -6.3 (9.5) | -7.4 to -5.2 | -16.1 (8.3) | -16.4 to -15.6 | -5.9 (9.4) | -7.4 to -4.5 | -14.9 (8.3) | -15.4 to -14.3 | -6.9 (9.7) | -8.6 to -5.1 | -17.1 (8.1) | -17.6 to -16.6 |
| LDL_de Cordova_ | 1.3 (33.3) | -2.6 to 5.1 | -20.5 (15.5) | -21.2 to -19.8 | 3.6 (27.4) | -0.7 to 7.8 | -17.5 (16.1) | -18.6 to -16.4 | -1.8 (39.7) | -8.9 to 5.3 | -23.3 (14.4) | -24.2 to -22.3 |
| LDL_Martin_ | -7.7 (11.8) | -9.1 to -6.3 | -13.3 (7.8) | -13.6 to -12.9 | -6.7 (13.6) | -8.8 to -4.6 | -12.1 (8) | -12.6 to -11.5 | -9 (8.7) | -10.6 to -7.5 | -14.4 (7.5) | -14.9 to -13.9 |
| LDL_Choi_ | 2.4 (8.5) | 1.5 to 0.4 | -0.1 (6.1) | -0.4 to 0.2 | 1.9 (10.4) | 0.3 to 0.5 | 0.1 (6.3) | -0.4 to 0.5 | 3.2 (4.8) | 2.3 to 0 | -0.2 (5.9) | -0.6 to 0.1 |

(*continue*)

**Supplementary Table S3**. (*continue*)

|  | **Population 3** | | | | | | | | | | | |
| --- | --- | --- | --- | --- | --- | --- | --- | --- | --- | --- | --- | --- |
|  | **Total** | | | | **Men** | | | | **Women** | | | |
|  | **LDL group 1 (LDL <70 mg/dL, Test n=83)** | | **LDL group 2 (LDL 70 mg/dL, Test n=806)** | | **LDL group 1 (LDL <70 mg/dL, Test n=64)** | | **LDL group 2 (LDL 70 mg/dL, Test n=524)** | | **LDL group 1 (LDL <70 mg/dL, Test n=19)** | | **LDL group 2 (LDL 70 mg/dL, Test n=282)** | |
| **Intraclass correlation** | **Coefficient** | **95%CI** | **Coefficient** | **95%CI** | **Coefficient** | **95%CI** | **Coefficient** | **95%CI** | **Coefficient** | **95%CI** | **Coefficient** | **95%CI** |
| LDL_Friedwald_ | 0.17 | -0.05 to 0.37 | 0.85 | 0.63 to 0.92 | 0.13 | -0.07 to 0.34 | 0.82 | 0.48 to 0.91 | 0.31 | -0.08 to 0.65 | 0.90 | 0.82 to 0.94 |
| LDL_DeLong_ | 0.35 | 0.15 to 0.52 | 0.92 | 0.90 to 0.93 | 0.33 | 0.09 to 0.53 | 0.91 | 0.90 to 0.93 | 0.41 | -0.06 to 0.73 | 0.92 | 0.89 to 0.94 |
| LDL_Rao_ | 0.36 | 0.15 to 0.53 | 0.88 | 0.86 to 0.89 | 0.37 | 0.14 to 0.56 | 0.87 | 0.84 to 0.89 | 0.28 | -0.22 to 0.65 | 0.89 | 0.87 to 0.91 |
| LDL_Hattori_ | 0.15 | -0.06 to 0.36 | 0.78 | 0.09 to 0.92 | 0.12 | -0.07 to 0.33 | 0.74 | 0.01 to 0.91 | 0.29 | -0.1 to 0.63 | 0.85 | 0.30 to 0.94 |
| LDL_Anadaraja_ | 0.18 | -0.03 to 0.39 | 0.83 | 0.55 to 0.92 | 0.15 | -0.06 to 0.37 | 0.78 | 0.36 to 0.90 | 0.33 | -0.07 to 0.66 | 0.91 | 0.81 to 0.94 |
| LDL_Ahmadi_ | 0.00 | -0.06 to 0.08 | 0.03 | -0.03 to 0.09 | 0.00 | -0.06 to 0.09 | 0.02 | -0.02 to 0.06 | -0.02 | -0.1 to 0.16 | 0.06 | -0.05 to 0.19 |
| LDL_Puavilai_ | 0.31 | 0.10 to 0.50 | 0.91 | 0.90 to 0.92 | 0.28 | 0.04 to 0.49 | 0.91 | 0.89 to 0.92 | 0.41 | -0.05 to 0.72 | 0.92 | 0.90 to 0.94 |
| LDL_Vujovic_ | 0.39 | 0.20 to 0.56 | 0.91 | 0.90 to 0.93 | 0.38 | 0.16 to 0.57 | 0.92 | 0.90 to 0.93 | 0.39 | -0.07 to 0.71 | 0.91 | 0.86 to 0.94 |
| LDL_Chen and Zhang_ | 0.44 | 0.22 to 0.61 | 0.91 | 0.90 to 0.92 | 0.48 | 0.25 to 0.66 | 0.92 | 0.91 to 0.93 | 0.29 | -0.11 to 0.63 | 0.90 | 0.88 to 0.93 |
| LDL_de Cordova_ | 0.09 | -0.07 to 0.26 | 0.78 | 0.73 to 0.81 | 0.11 | -0.07 to 0.31 | 0.76 | 0.71 to 0.80 | 0.02 | -0.14 to 0.27 | 0.80 | 0.74 to 0.84 |
| LDL_Martin_ | 0.31 | 0.06 to 0.51 | 0.91 | 0.89 to 0.93 | 0.32 | 0.06 to 0.53 | 0.92 | 0.90 to 0.93 | 0.24 | -0.12 to 0.58 | 0.90 | 0.84 to 0.93 |
| LDL_Cho_i | 0.38 | 0.11 to 0.59 | 0.84 | 0.27 to 0.94 | 0.41 | 0.14 to 0.62 | 0.85 | 0.31 to 0.94 | 0.26 | -0.1 to 0.61 | 0.82 | 0.17 to 0.94 |
| **Systemic differences** | **Mean (SD)** | **95%CI** | **Mean (SD)** | **95%CI** | **Mean (SD)** | **95%CI** | **Mean (SD)** | **95%CI** | **Mean (SD)** | **95%CI** | **Mean (SD)** | **95%CI** |
| LDL_Friedwald_ | -24.4 (28.0) | -30.6 to -18.3 | -11.4 (16.5) | -12.5 to -10.2 | -27.7 (29.2) | -35.0 to -20.3 | -13.4 (16.9) | -14.8 to -11.9 | -13.5 (20.6) | -23.5 to -3.6 | -7.6 (15.1) | -9.4 to -5.8 |
| LDL_DeLong_ | -7.0 (21.0) | -11.6 to -2.5 | 0.6 (14.3) | -0.4 to 1.5 | -9.2 (21.3) | -14.5 to -3.8 | -1.1 (13.8) | -2.3 to 0.1 | 0.1 (18.5) | -8.9 to 9.0 | 3.7 (14.9) | 1.9 to 5.4 |
| LDL_Rao_ | 1.5 (21.3) | -3.1 to 6.2 | -0.5 (17.2) | -1.6 to 0.7 | 1.7 (22) | -3.8 to 7.2 | -1.5 (16.9) | -3 to -0.1 | 0.7 (19.2) | -8.5 to 10.0 | 1.6 (17.5) | -0.5 to 3.6 |
| LDL_Hattori_ | -27.1 (26.8) | -32.9 to -21.2 | -18.9 (15.6) | -20 to -17.7 | -30.1 (27.9) | -37.1 to -23.1 | -20.8 (15.9) | -22.2 to -19.4 | -16.8 (19.7) | -26.3 to -7.3 | -15.3 (14.3) | -17.0 to -13.6 |
| LDL_Anadaraja_ | -21.4 (27.6) | -27.4 to -15.3 | -12.6 (16.9) | -13.8 to -11.4 | -23.8 (29.2) | -31.1 to -16.5 | -15.2 (17.5) | -16.7 to -13.7 | -13.2 (20.1) | -22.9 to -3.5 | -7.8 (14.5) | -9.5 to -6.1 |
| LDL_Ahmadi_ | 230.1 (153.9) | 196.5 to 263.7 | 136.4 (88.6) | 130.3 to 142.5 | 246.6 (163) | 205.9 to 287.3 | 140.8 (95.5) | 132.6 to 149 | 174.6 (103.4) | 124.8 to 224.4 | 128.2 (73.4) | 119.6 to 136.7 |
| LDL_Puavilai_ | -9.9 (22.0) | -14.7 to -5.1 | -1.4 (14.6) | -2.4 to -0.4 | -12.2 (22.5) | -17.8 to -6.6 | -3.1 (14.2) | -4.3 to -1.9 | -2.2 (18.7) | -11.2 to 6.8 | 1.9 (14.9) | 0.1 to 3.6 |
| LDL_Vujovic_ | -3.6 (19.9) | -8.0 to 0.7 | 2.9 (14.1) | 1.9 to 3.9 | -5.5 (20.1) | -10.5 to -0.5 | 1.3 (13.4) | 0.2 to 0.5 | 2.8 (18.4) | -6.1 to 11.7 | 5.9 (15) | 4.1 to 7.7 |
| LDL_Chen and Zhang_ | 7.2 (15.3) | 3.8 to 10.5 | 1.0 (13.4) | 0.1 to 2.0 | 6.5 (14.8) | 2.8 to 0.3 | -0.1 (12.2) | -1.1 to 1.0 | 9.3 (17.1) | 1.1 to 17.6 | 3.1 (15.3) | 1.3 to 4.9 |
| LDL_de Cordova_ | 33 (24.4) | 27.6 to 38.3 | 4.9 (20.1) | 3.5 to 6.3 | 34.8 (24.8) | 28.6 to 41.0 | 4.7 (19.7) | 3.0 to 6.4 | 26.6 (22.5) | 15.8 to 37.5 | 5.2 (20.8) | 2.8 to 7.7 |
| LDL_Martin_ | 11.1 (17.8) | 7.2 to 15.0 | 3.7 (13.5) | 2.7 to 4.6 | 10.5 (17.9) | 6.0 to 5.0 | 2.4 (12.4) | 1.3 to 3.5 | 13.2 (17.5) | 4.8 to 21.7 | 6.1 (15) | 4.3 to 7.8 |
| LDL_Choi_ | 10.8 (17.3) | 7.0 to 14.6 | 15.0 (13.9) | 14.1 to 16.0 | 9.5 (17) | 5.3 to 3.8 | 13.5 (12.8) | 12.4 to 14.6 | 15.1 (18.0) | 6.4 to 23.7 | 17.9 (15.3) | 16.1 to 19.7 |

The top three equations are presented in color: red represents the highest, orange represents the second, and yellow represents the third highest intraclass correlation coefficient, or the equations with the lowest, second lowest, and third lowest mean systemic differences.

**Supplementary Table S4**. Limits of agreement and absolute error among the 12 equations in comparison with directly measured LDL by subgroup of LDL concentration

|  | **Population 1** | | | | | | | | | | | | | | | | | | | | | | | |
| --- | --- | --- | --- | --- | --- | --- | --- | --- | --- | --- | --- | --- | --- | --- | --- | --- | --- | --- | --- | --- | --- | --- | --- | --- |
|  | **Total (Test n=5198)** | | | | | | | | **Men (Test n=2570)** | | | | | | | | **Women (Test n=2628)** | | | | | | | |
|  | **LDL group 1 (LDL <70 mg/dL, Test n=581)** | | | | **LDL group 2 (LDL 70 mg/dL, Test n=4617)** | | | | **LDL group 1 (LDL <70 mg/dL, Test n=357)** | | | | **LDL group 2 (LDL 70 mg/dL, Test n=2213)** | | | | **LDL group 1 (LDL <70 mg/dL, Test n=224)** | | | | **LDL group 2 (LDL 70 mg/dL, Test n=2404)** | | | |
| **Limits of agreement** | **Lower** | **95%CI** | **Upper** | **95%CI** | **Lower** | **95%CI** | **Upper** | **95%CI** | **Lower** | **95%CI** | **Upper** | **95%CI** | **Lower** | **95%CI** | **Upper** | **95%CI** | **Lower** | **95%CI** | **Upper** | **95%CI** | **Lower** | **95%CI** | **Upper** | **95%CI** |
| LDL_Friedwald_ | -48.1 | -50.3 to -45.9 | 14.3 | 12.1 to 16.5 | -41.9 | -42.5 to -41.3 | 3.4 | 2.8 to 3.9 | -56.1 | -59.5 to 52.7 | 18.1 | 14.7 to 21.4 | -47.8 | -48.7 to -46.8 | 5.2 | 4.3 to 6.2 | -29.8 | -31.7 to -27.9 | 2.7 | 0.8 to 4.5 | -35 | -35.6 to -34.4 | 0.2 | -0.4 to 0.8 |
| LDL_DeLong_ | -29.5 | -30.9 to -28 | 11.3 | 9.9 to 2.8 | -28.3 | -28.7 to -27.9 | 3.1 | 2.7 to 3.5 | -33.5 | -35.7 to 31.4 | 14.1 | 11.9 to 16.2 | -31.2 | -31.9 to -30.6 | 4.4 | 3.7 to 5 | -21.1 | -22.6 to -19.6 | 5.1 | 3.6 to 6.6 | -25.2 | -25.7 to -24.8 | 1.6 | 1.1 to 2 |
| LDL_Rao_ | -182.6 | -195.2 to -169.9 | 173.9 | 161.2 to 186.5 | -34.1 | -34.6 to -33.6 | 4.3 | 3.8 to 4.8 | -227.3 | -247.9 to -206.7 | 226.3 | 205.7 to 246.9 | -39.4 | -40.3 to -38.5 | 9.3 | 8.4 to 10.2 | -26.4 | -28.2 to -24.6 | 5.2 | 3.4 to 7 | -27.4 | -27.8 to -27 | -2.1 | -2.5 to -1.6 |
| LDL_Hattori_ | -49.8 | -52 to 47.7 | 10.4 | 8.3 to 2.6 | -48.2 | -48.8 to -47.7 | -3.2 | -3.8 to -2.6 | -57.5 | -60.8 to 54.3 | 14.1 | 10.8 to 17.3 | -53.8 | -54.7 to -52.8 | -1.4 | -2.4 to -0.5 | -32.2 | -34 to -30.4 | -0.8 | -2.6 to 1 | -41.9 | -42.5 to -41.3 | -6.1 | -6.7 to -5.4 |
| LDL_Anadaraja_ | -46 | -49.1 to 43 | 40 | 36.9 to 43.1 | -46.1 | -47.1 to -45.2 | 29.7 | 28.7 to 30.6 | -53 | -57.2 to 48.8 | 39.7 | 35.5 to 43.9 | -51.4 | -52.7 to -50 | 22.6 | 21.3 to 23.9 | -31.4 | -35.3 to -27.5 | 37 | 33.1 to 40.9 | -37.7 | -39 to -36.5 | 32.6 | 31.4 to 33.9 |
| LDL_Ahmadi_ | -298.2 | -324.4 to -272 | 439.9 | 413.6 to 466.1 | -122.4 | -126.4 to -118.3 | 199.4 | 195.4 to 203.5 | -357.8 | -398.8 to -316.8 | 546 | 505 to 587.1 | -132.9 | -139.8 to -126 | 248.8 | 241.8 to 255.7 | -106.1 | -122.1 to -90 | 173.5 | 157.4 to 189.6 | -96.2 | -100.2 to -92.1 | 137.5 | 133.5 to 141.6 |
| LDL_Puavilai_ | -31.3 | -32.7 to -29.8 | 10.7 | 9.2 to 2.2 | -30.3 | -30.7 to -29.9 | 3 | 2.6 to 3.4 | -35.6 | -37.9 to 33.4 | 13.2 | 11 to 15.5 | -33.7 | -34.3 to -33 | 4.3 | 3.6 to 5 | -22.1 | -23.7 to -20.6 | 4.4 | 2.9 to 6 | -26.6 | -27 to -26.1 | 1.2 | 0.7 to 1.7 |
| LDL_Vujovic_ | -28.1 | -29.6 to -26.7 | 13.1 | 11.6 to 14.5 | -26.1 | -26.4 to -25.7 | 3.5 | 3.2 to 3.9 | -32 | -34.2 to 29.8 | 16.2 | 14 to 18.4 | -28.4 | -29 to -27.8 | 4.7 | 4.1 to 5.3 | -20 | -21.5 to -18.5 | 6.1 | 4.6 to 7.6 | -23.6 | -24.1 to -23.2 | 2.1 | 1.7 to 2.6 |
| LDL_Chen and Zhang_ | -35.2 | -37.3 to -33.1 | 24.7 | 22.6 to 26.9 | -31.1 | -31.5 to -30.7 | -1 | -1.4 to -0.7 | -40.6 | -43.9 to -37.3 | 32.1 | 28.8 to 35.4 | -31.4 | -31.9 to -30.8 | 0.6 | 0 to 1.1 | -21.4 | -23 to -19.7 | 7.7 | 6 to 9.3 | -30.7 | -31.2 to -30.2 | -2.7 | -3.2 to -2.2 |
| LDL_de Cordova_ | -66.2 | -71.1 to -61.3 | 71.2 | 66.3 to 76.1 | -50.5 | -51.3 to -49.8 | 11.5 | 10.7 to 12.3 | -77.8 | -85.4 to -70.1 | 90.6 | 82.9 to 98.2 | -51 | -52.2 to -49.7 | 18.4 | 17.1 to 19.6 | -30.5 | -33.5 to -27.4 | 23 | 20 to 26.1 | -48.4 | -49.3 to -47.5 | 3.4 | 2.5 to 4.3 |
| LDL_Martin_ | -27.5 | -29 to -26 | 15.7 | 14.2 to 17.2 | -27.7 | -28 to -27.3 | 0.9 | 0.6 to 1.3 | -29.2 | -31.4 to -27 | 19.5 | 17.3 to 21.7 | -28.4 | -28.9 to -27.8 | 2.7 | 2.1 to 0.3 | -23.3 | -25.1 to -21.5 | 8.2 | 6.4 to 10 | -26.9 | -27.3 to -26.4 | -0.8 | -1.3 to -0.4 |
| LDL_Choi_ | -21.3 | -23.1 to -19.6 | 28.1 | 26.4 to 29.9 | -13.4 | -13.7 to -13 | 12.4 | 12.1 to 12.7 | -26.3 | -29 to -23.6 | 33.4 | 30.7 to 36.1 | -15.3 | -15.8 to -14.7 | 13.1 | 12.6 to 13.6 | -9.9 | -11.4 to -8.4 | 16.2 | 14.7 to 17.7 | -11.4 | -11.8 to -11 | 11.5 | 11.1 to 11.9 |
| **Absolute percentage error** | **Med** | **95% CI** | **95th P** | **95% CI** | **Med** | **95% CI** | **95th P** | **95% CI** | **Med** | **95% CI** | **95th P** | **95% CI** | **Med** | **95% CI** | **95th P** | **95% CI** | **Med** | **95% CI** | **95th P** | **95% CI** | **Med** | **95% CI** | **95th P** | **95% CI** |
| LDL_Friedwald_ | 23.2 | 22.1 to 24.2 | 88.7 | 78.6 to 115.2 | 14.8 | 14.6 to 15.1 | 34.8 | 33.7 to 36.5 | 25.0 | 23 to 26.8 | 115.5 | 89.8 to 144.4 | 16.0 | 15.6 to 16.3 | 39.8 | 37.8 to 41.3 | 21.8 | 20.3 to 23.1 | 45.8 | 40.8 to 67.3 | 13.9 | 13.5 to 14.1 | 28.2 | 27 to 29.5 |
| LDL_DeLong_ | 15.0 | 14.3 to 16 | 52.5 | 46 to 64.7 | 10.2 | 10 to 10.4 | 22.2 | 21.4 to 22.9 | 16.2 | 14.8 to 17.3 | 66.3 | 53.1 to 76.3 | 10.7 | 10.4 to 11 | 24.0 | 23.4 to 25.3 | 13.9 | 13 to 15 | 29.6 | 26.9 to 44.8 | **9.8** | 9.5 to 10 | 20.1 | 19.5 to 21 |
| LDL_Rao_ | 20.4 | 19.6 to 21.5 | 48.9 | 43.8 to 59 | 12.8 | 12.6 to 13 | 24.3 | 23.8 to 25 | 21.5 | 20.2 to 23.1 | 59.5 | 50.2 to 82.4 | 13.3 | 13 to 13.6 | 25.9 | 25.2 to 27 | 19.3 | 18.1 to 20.5 | 35.0 | 32.3 to 38.9 | 12.4 | 12.1 to 12.7 | 23.0 | 22.4 to 23.8 |
| LDL_Hattori_ | 28.1 | 27 to 29.2 | 90.8 | 81.3 to 115.3 | 20.2 | 19.9 to 20.5 | 39.5 | 38.3 to 41.1 | 30.2 | 27.8 to 32.1 | 115.6 | 92.6 to 144.4 | 21.3 | 20.9 to 21.6 | 44.0 | 42.2 to 45.5 | 26.7 | 25.3 to 28 | 50.3 | 44.7 to 70.7 | 19.2 | 19 to 19.6 | 32.9 | 31.8 to 34.3 |
| LDL_Anadaraja_ | 17.2 | 15.9 to 19.7 | 89.5 | 73.8 to 112 | **10.9** | 10.5 to 11.3 | 36.6 | 35.1 to 38.1 | 18.0 | 15.9 to 22 | 110.7 | 89.3 to 134.4 | 12.4 | 11.9 to 13 | 40.2 | 38.2 to 41.8 | 17.1 | 14.7 to 20 | 68.4 | 57.4 to 85.8 | **9.7** | 9.3 to 10.2 | 32.1 | 30.2 to 33.9 |
| LDL_Ahmadi_ | 50.0 | 44.4 to 56.5 | 521.0 | 434.2 to 762.4 | 23.7 | 22.9 to 24.6 | 165.7 | 156.9 to 174.9 | 61.9 | 52.1 to 77.5 | 751.7 | 523.7 to 1094 | 29.5 | 27.7 to 31.6 | 200.7 | 185.4 to 214.1 | 41.3 | 33.8 to 46.4 | 343.5 | 219.8 to 439 | 20.5 | 19.4 to 21.5 | 113.5 | 100 to 127 |
| LDL_Puavilai_ | 16.7 | 15.2 to 17.4 | 55.2 | 51.2 to 71.5 | **11.0** | 10.7 to 11.1 | 24.0 | 23.3 to 24.7 | 17.5 | 16.2 to 18.9 | 72.1 | 57.8 to 86.2 | **11.5** | 11.2 to 11.8 | 26.1 | 25.1 to 27.5 | 15.1 | 13.9 to 16.4 | 32.1 | 28.7 to 50.3 | **10.4** | 10.1 to 10.7 | 21.4 | 20.7 to 22.3 |
| LDL_Vujovic_ | 13.4 | 12.7 to 14.3 | 46.9 | 41.3 to 53.6 | 9.2 | 9 to 9.4 | 20.2 | 19.7 to 20.8 | 14.3 | 13.1 to 15.4 | 52.9 | 47.1 to 64.1 | 9.6 | 9.3 to 9.9 | 22.0 | 20.8 to 22.9 | 12.2 | 11.1 to 13.9 | 27.5 | 25.3 to 37.5 | 8.9 | 8.7 to 9.1 | 18.8 | 18.1 to 19.7 |
| LDL_Chen and Zhang_ | 13.9 | 13.1 to 14.3 | 30.8 | 28.1 to 35.1 | 13.6 | 13.4 to 13.7 | 21.4 | 21.1 to 21.8 | 13.8 | 12.4 to 14.6 | 35.2 | 30.1 to 43.9 | 13.3 | 13 to 13.6 | 21.3 | 20.7 to 21.8 | 13.9 | 13.1 to 14.5 | 26.2 | 23.6 to 30.3 | 13.8 | 13.6 to 14 | 21.5 | 21.1 to 22 |
| LDL_de Cordova_ | 13.9 | 12.9 to 14.9 | 78.4 | 54.1 to 99.4 | 18.6 | 18.3 to 18.8 | 28.8 | 28.5 to 29.2 | 13.4 | 11.9 to 15 | 100.5 | 75.4 to 157.3 | 16.9 | 16.4 to 17.3 | 28.0 | 27.4 to 28.8 | 14.3 | 12.8 to 15.9 | 43.4 | 32.1 to 57.3 | 19.6 | 19.3 to 19.9 | 29.2 | 28.8 to 29.6 |
| LDL_Martin_ | 15.6 | 14.7 to 16.4 | 35.1 | 31.9 to 37.9 | **11.3** | 11.2 to 11.5 | 20.3 | 19.8 to 20.6 | 15.1 | 13.8 to 16.4 | 36.4 | 31 to 48.5 | **11.1** | 10.7 to 11.3 | 19.8 | 19.3 to 20.4 | 16.1 | 14.8 to 17.6 | 34.2 | 29.6 to 37.1 | **11.5** | 11.3 to 11.7 | 20.6 | 20 to 21.1 |
| LDL_Choi_ | **6.5** | 5.9 to 7 | 26.2 | 24.5 to 30 | **3.1** | 3 to 3.2 | **10.7** | 10.2 to 11.2 | **7.0** | 6.2 to 7.7 | 28.3 | 25 to 44 | **3.3** | 3.1 to 3.5 | **11.9** | 11 to 12.7 | **5.8** | 4.8 to 6.6 | 25.0 | 19.2 to 28.6 | **2.9** | 2.8 to 3.1 | **9.8** | 9.1 to 10.3 |

*(continue)*

|  | **Population 2** | | | | | | | | | | | | | | | | | | | | | | | |
| --- | --- | --- | --- | --- | --- | --- | --- | --- | --- | --- | --- | --- | --- | --- | --- | --- | --- | --- | --- | --- | --- | --- | --- | --- |
|  | **Total (Test n=2,163)** | | | | | | | | **Men (Test n=1,062)** | | | | | | | | **Women (Test n=1,101)** | | | | | | | |
|  | **LDL group 1 (LDL <70 mg/dL, Test n=287)** | | | | **LDL group 2 (LDL 70 mg/dL, Test n=1876)** | | | | **LDL group 1 (LDL <70 mg/dL, Test n=164)** | | | | **LDL group 2 (LDL 70 mg/dL, Test n=898)** | | | | **LDL group 1 (LDL <70 mg/dL, Test n=123)** | | | | **LDL group 2 (LDL 70 mg/dL, Test n=978)** | | | |
| **Limits of agreement** | **Lower** | **95%CI** | **Upper** | **95%CI** | **Lower** | **95%CI** | **Upper** | **95%CI** | **Lower** | **95%CI** | **Upper** | **95%CI** | **Lower** | **95%CI** | **Upper** | **95%CI** | **Lower** | **95%CI** | **Upper** | **95%CI** | **Lower** | **95%CI** | **Upper** | **95%CI** |
| LDL_Friedwald_ | -68.6 | -73.8 to -63.5 | 33.3 | 28.2 to 38.5 | -35.3 | -35.9 to -34.6 | -1.1 | -1.8 to -0.4 | -70.3 | -77.1 to -63.4 | 31.7 | 24.8 to 38.5 | -37.4 | -38.4 to -36.3 | -0.5 | -1.6 to 0.5 | -66.4 | -74.3 to -58.4 | 35.4 | 27.5 to 43.4 | -33.1 | -34 to -32.3 | -1.8 | -2.7 to -0.9 |
| LDL_DeLong_ | -37.8 | -40.6 to -35 | 17.6 | 14.8 to 20.4 | -25.4 | -26 to -24.9 | 1.4 | 0.9 to 2 | -42 | -46.2 to -37.8 | 20.3 | 16.1 to 24.5 | -25.8 | -26.6 to -25 | 2 | 1.2 to 2.8 | -31.4 | -34.9 to -27.9 | 13.2 | 9.7 to 16.7 | -25.1 | -25.8 to -24.4 | 0.9 | 0.2 to 1.6 |
| LDL_Rao_ | -218.4 | -240.3 to -196.5 | 214.5 | 192.5 to 236.4 | -28.1 | -28.6 to -27.6 | -1.6 | -2.1 to -1.1 | -106.9 | -120.5 to -93.3 | 95.3 | 81.7 to 108.9 | -28.7 | -29.5 to -27.9 | -0.6 | -1.4 to 0.2 | -306.7 | -355 to -258.3 | 313 | 264.7 to 361.4 | -27.5 | -28.2 to -26.8 | -2.6 | -3.3 to -1.9 |
| LDL_Hattori_ | -69.3 | -74.3 to -64.3 | 28.7 | 23.7 to 33.6 | -42.4 | -43.1 to -41.7 | -6.9 | -7.6 to -6.2 | -70.5 | -77.1 to -64 | 26.9 | 20.3 to 33.5 | -44.2 | -45.2 to -43.1 | -6.6 | -7.6 to -5.5 | -67.6 | -75.2 to -59.9 | 31 | 23.3 to 38.6 | -40.6 | -41.5 to -39.7 | -7.4 | -8.3 to -6.5 |
| LDL_Anadaraja_ | -58.8 | -64.7 to -53 | 56 | 50.2 to 61.8 | -38.6 | -39.9 to -37.3 | 26 | 24.8 to 27.3 | -63.3 | -71.1 to -55.6 | 51.2 | 43.5 to 58.9 | -41.6 | -43.3 to -39.8 | 19 | 17.3 to 20.7 | -50.7 | -59.4 to -42.1 | 60.3 | 51.6 to 69 | -33.1 | -34.9 to -31.4 | 29.8 | 28.1 to 31.5 |
| LDL_Ahmadi_ | -351.7 | -394 to -309.4 | 482.6 | 440.3 to 524.9 | -100.2 | -105.4 to -95 | 162 | 156.9 to 167.2 | -276.9 | -324.9 to -228.9 | 436.2 | 388.2 to 484.3 | -96.1 | -104.2 to -88 | 188.3 | 180.2 to 196.5 | -439 | -514.7 to -363.2 | 532 | 456.2 to 607.8 | -96.5 | -102.7 to -90.3 | 130.3 | 124.1 to 136.5 |
| LDL_Puavilai_ | -42.6 | -45.8 to -39.4 | 20 | 16.8 to 23.2 | -26.8 | -27.3 to -26.2 | 0.9 | 0.3 to 1.4 | -46.4 | -51 to -41.8 | 22.1 | 17.5 to 26.7 | -27.4 | -28.2 to -26.6 | 1.5 | 0.6 to 2.3 | -37 | -41.2 to -32.8 | 16.8 | 12.6 to 21 | -26.2 | -26.9 to -25.5 | 0.3 | -0.4 to 1.1 |
| LDL_Vujovic_ | -32.3 | -34.7 to -29.9 | 15.2 | 12.7 to 17.6 | -23.9 | -24.5 to -23.4 | 2.4 | 1.9 to 3 | -36.8 | -40.5 to -33.1 | 18.5 | 14.8 to 22.2 | -24 | -24.8 to -23.2 | 3.1 | 2.3 to 3.9 | -25 | -27.6 to -22.3 | 9.3 | 6.7 to 12 | -23.9 | -24.6 to -23.2 | 1.8 | 1.1 to 2.5 |
| LDL_Chen and Zhang_ | -24.9 | -26.8 to -23 | 12.3 | 10.4 to 14.1 | -32.3 | -32.9 to -31.6 | 0.2 | -0.5 to 0.8 | -24.3 | -26.7 to -21.8 | 12.4 | 9.9 to 14.9 | -31.1 | -32 to -30.2 | 1.3 | 0.4 to 2.3 | -25.8 | -28.8 to -22.8 | 12.1 | 9.1 to 15 | -33.1 | -33.9 to -32.2 | -1.2 | -2.1 to -0.3 |
| LDL_de Cordova_ | -63.9 | -70.5 to -57.3 | 66.5 | 59.9 to 73.1 | -50.8 | -52 to -49.6 | 9.8 | 8.6 to 11 | -50.1 | -57.4 to -42.9 | 57.3 | 50 to 64.5 | -49 | -50.8 to -47.2 | 14 | 12.2 to 15.8 | -79.5 | -91.7 to -67.4 | 76 | 63.8 to 88.1 | -51.4 | -52.9 to -49.8 | 4.9 | 3.3 to 6.4 |
| LDL_Martin_ | -30.9 | -33.2 to -28.5 | 15.5 | 13.1 to 17.8 | -28.7 | -29.3 to -28.1 | 2.1 | 1.5 to 2.7 | -33.4 | -37 to -29.8 | 20 | 16.4 to 23.6 | -27.8 | -28.7 to -26.9 | 3.7 | 2.8 to 4.6 | -26.1 | -28.8 to -23.5 | 8 | 5.4 to 10.7 | -29.1 | -29.9 to -28.3 | 0.3 | -0.5 to 1.1 |
| LDL_Choi_ | -14.2 | -15.8 to -12.5 | 19 | 17.4 to 20.7 | -12 | -12.4 to -11.5 | 11.8 | 11.3 to 12.2 | -18.5 | -21.2 to -15.7 | 22.3 | 19.5 to 25 | -12.2 | -12.9 to -11.5 | 12.3 | 11.6 to 13 | -6.2 | -7.6 to -4.7 | 12.5 | 11.1 to 14 | -11.7 | -12.4 to -11.1 | 11.2 | 10.6 to 11.9 |
| **Absolute percentage error** | **Med** | **95% CI** | **95th P** | **95% CI** | **Med** | **95% CI** | **95th P** | **95% CI** | **Med** | **95% CI** | **95th P** | **95% CI** | **Med** | **95% CI** | **95th P** | **95% CI** | **Med** | **95% CI** | **95th P** | **95% CI** | **Med** | **95% CI** | **95th P** | **95% CI** |
| LDL_Friedwald_ | 23.2 | 21.8 to 24.4 | 69.0 | 54.9 to 113.5 | 14.4 | 14.1 to 14.7 | 30.0 | 28.4 to 31.1 | 23.7 | 21.4 to 26.6 | 83.7 | 65 to 363.5 | 14.7 | 14.3 to 15.2 | 33.2 | 30.9 to 36.2 | 22.5 | 20.4 to 24 | 43.5 | 40.4 to 72.6 | 14.1 | 13.5 to 14.6 | 26.0 | 24.3 to 27.5 |
| LDL_DeLong_ | 15.2 | 13.7 to 17.4 | 38.7 | 33.1 to 70.9 | 9.9 | 9.7 to 10.2 | 20.9 | 19.6 to 21.7 | 15.1 | 13.2 to 17.7 | 48.1 | 37.1 to 175.1 | 9.8 | 9.5 to 10.2 | 22.3 | 21.1 to 23.9 | 15.4 | 13.1 to 18 | 29.9 | 26.9 to 38.4 | 10.1 | 9.7 to 10.5 | 18.9 | 18.1 to 20.5 |
| LDL_Rao_ | 20.8 | 18.8 to 22 | 41.7 | 38.8 to 64.8 | 12.6 | 12.2 to 12.9 | 23.1 | 22.6 to 24.3 | 20.6 | 18.3 to 22.4 | 48.9 | 40.7 to 159.9 | 12.6 | 12 to 13 | 24.9 | 23.5 to 25.8 | 21.2 | 18.2 to 22.5 | 35.0 | 32.9 to 41.7 | 12.7 | 12.2 to 13.2 | 22.1 | 21.2 to 22.9 |
| LDL_Hattori_ | 28.3 | 27 to 29.4 | 72.9 | 58.4 to 113.8 | 19.8 | 19.5 to 20 | 34.8 | 33.3 to 35.9 | 28.8 | 26.9 to 30.5 | 88.4 | 69.9 to 355 | 20.1 | 19.7 to 20.6 | 37.9 | 35.9 to 40.8 | 27.9 | 25.8 to 29.1 | 47.6 | 44.5 to 75.9 | 19.5 | 19 to 20 | 30.6 | 29.4 to 32.3 |
| LDL_Anadaraja_ | 19.1 | 16.8 to 21.4 | 88.2 | 65.6 to 126.5 | 9.8 | 9.3 to 10.3 | 30.7 | 29.2 to 33 | 19.3 | 17 to 22.2 | 98.7 | 65.4 to 261.2 | 11.1 | 10.3 to 11.8 | 32.8 | 30 to 36 | 18.0 | 13.2 to 22.9 | 78.2 | 53.1 to 127.5 | 8.6 | 8.2 to 9.3 | 29.2 | 25.9 to 32 |
| LDL_Ahmadi_ | 44.4 | 39.1 to 53.8 | 491.1 | 344.7 to 930 | 24.3 | 22.9 to 25.7 | 146.0 | 127.4 to 159.7 | 57.6 | 43.3 to 77.5 | 850.2 | 471.6 to 1761.5 | 28.8 | 26 to 31.2 | 177.1 | 155.7 to 198.3 | 37.8 | 31.5 to 43.5 | 195.0 | 154.4 to 581.3 | 21.4 | 19.1 to 23.1 | 106.6 | 90.9 to 123.4 |
| LDL_Puavilai_ | 16.4 | 14.8 to 18.5 | 41.4 | 36.2 to 77.5 | 10.6 | 10.3 to 10.9 | 21.9 | 20.9 to 22.8 | 16.4 | 14.6 to 18.8 | 52.3 | 36.4 to 187.8 | 10.6 | 10.1 to 11 | 23.9 | 22.6 to 25.3 | 16.4 | 14.1 to 19.3 | 31.8 | 28.3 to 43 | 10.7 | 10.2 to 11.1 | 19.7 | 18.9 to 21.1 |
| LDL_Vujovic_ | 14.1 | 12.3 to 15.8 | 33.4 | 28.5 to 56.4 | 9.0 | 8.8 to 9.3 | 19.2 | 18.4 to 20.3 | 14.2 | 12.1 to 16.3 | 40.3 | 30.7 to 160.1 | 8.8 | 8.4 to 9.2 | 20.6 | 19.3 to 21.9 | 13.6 | 11.4 to 17.1 | 27.2 | 24 to 35.4 | 9.2 | 8.9 to 9.5 | 17.8 | 17 to 18.8 |
| LDL_Chen and Zhang_ | 14.3 | 12.3 to 15.5 | 29.7 | 25.8 to 65.3 | 13.8 | 13.5 to 14 | 21.9 | 21.3 to 22.5 | 13.2 | 11.7 to 15.2 | 37.1 | 25.3 to 100.1 | 13.0 | 12.5 to 13.5 | 21.2 | 20.3 to 22.4 | 15.3 | 12.4 to 16.4 | 26.6 | 24.8 to 37.6 | 14.4 | 14 to 14.7 | 22.2 | 21.6 to 22.9 |
| LDL_de Cordova_ | 14.3 | 11.8 to 16.6 | 59.0 | 43.7 to 98.2 | 18.9 | 18.4 to 19.3 | 29.9 | 29.4 to 30.5 | 16.1 | 11.9 to 18.8 | 78.4 | 55.9 to 277.7 | 17.2 | 16.5 to 17.9 | 28.6 | 27.6 to 30.2 | 12.3 | 10.3 to 15.7 | 37.0 | 29.7 to 82.5 | 20.5 | 19.8 to 21 | 30.4 | 29.8 to 31.1 |
| LDL_Martin_ | 16.4 | 14.7 to 17.7 | 33.0 | 30.5 to 66.4 | 11.7 | 11.5 to 12 | 20.6 | 20.2 to 21.4 | 15.0 | 13.6 to 17.7 | 47.5 | 29.6 to 105.4 | 10.9 | 10.4 to 11.5 | 20.1 | 19.4 to 21.4 | 17.5 | 15.2 to 18.8 | 31.7 | 28.8 to 49.1 | 12.2 | 11.9 to 12.6 | 21.0 | 20.3 to 21.8 |
| LDL_Choi_ | 6.1 | 4.9 to 6.8 | 29.2 | 23.6 to 80.7 | 3.1 | 3 to 3.3 | 10.3 | 9.4 to 10.9 | 6.4 | 5.2 to 7.5 | 54.9 | 26.1 to 132.8 | 3.2 | 2.8 to 3.4 | 10.9 | 10 to 12.3 | 4.9 | 3.9 to 6.9 | 23.2 | 18.4 to 29.4 | 3.1 | 2.9 to 3.4 | 9.3 | 8.6 to 10.5 |

*(continue)*

|  | **Population 3** | | | | | | | | | | | | | | | | | | | | | | | |
| --- | --- | --- | --- | --- | --- | --- | --- | --- | --- | --- | --- | --- | --- | --- | --- | --- | --- | --- | --- | --- | --- | --- | --- | --- |
|  | **Total (Test n=889)** | | | | | | | | **Men (Test n=588)** | | | | | | | | **Women (Test n=301)** | | | | | | | |
|  | **LDL group 1 (LDL <70 mg/dL, Test n=83)** | | | | **LDL group 2 (LDL 70 mg/dL, Test n=806)** | | | | **LDL group 1 (LDL <70 mg/dL, Test n=64)** | | | | **LDL group 2 (LDL 70 mg/dL, Test n=524)** | | | | **LDL group 1 (LDL <70 mg/dL, Test n=19)** | | | | **LDL group 2 (LDL 70 mg/dL, Test n=282)** | | | |
| **Limits of agreement** | **Lower** | **95%CI** | **Upper** | **95%CI** | **Lower** | **95%CI** | **Upper** | **95%CI** | **Lower** | **95%CI** | **Upper** | **95%CI** | **Lower** | **95%CI** | **Upper** | **95%CI** | **Lower** | **95%CI** | **Upper** | **95%CI** | **Lower** | **95%CI** | **Upper** | **95%CI** |
| LDL_Friedwald_ | -79.4 | -89.9 to -68.9 | 30.5 | 20 to 41 | -43.7 | -45.7 to -41.8 | 21 | 19.1 to 23 | -84.9 | -97.5 to -72.4 | 29.6 | 17 to 42.1 | -46.5 | -49 to -44 | 19.7 | 17.3 to 22.2 | -53.9 | -71.2 to -36.6 | 26.9 | 9.6 to 44.2 | -37.2 | -40.2 to -34.1 | 22 | 19 to 25 |
| LDL_DeLong_ | -48.2 | -56 to -40.3 | 34.1 | 26.2 to 41.9 | -27.6 | -29.2 to -25.9 | 28.7 | 27 to 30.3 | -51 | -60.1 to -41.8 | 32.7 | 23.5 to 41.8 | -28.1 | -30.1 to -26.1 | 25.8 | 23.8 to 27.9 | -36.2 | -51.8 to -20.7 | 36.3 | 20.8 to 51.9 | -25.5 | -28.5 to -22.5 | 32.9 | 29.9 to 35.8 |
| LDL_Rao_ | -40.2 | -48.2 to -32.2 | 43.2 | 35.2 to 51.2 | -34.1 | -36.1 to -32.1 | 33.2 | 31.2 to 35.2 | -41.4 | -50.8 to -32 | 44.9 | 35.4 to 54.3 | -34.6 | -37.1 to -32.2 | 31.6 | 29.1 to 34.1 | -36.8 | -52.9 to -20.8 | 38.3 | 22.2 to 54.4 | -32.8 | -36.3 to -29.3 | 35.9 | 32.4 to 39.4 |
| LDL_Hattori_ | -79.5 | -89.6 to -69.5 | 25.4 | 15.4 to 35.4 | -49.4 | -51.3 to -47.6 | 11.7 | 9.8 to 3.5 | -84.9 | -96.9 to -72.9 | 24.7 | 12.7 to 36.7 | -52 | -54.4 to -49.7 | 10.4 | 8.1 to 2.8 | -55.3 | -71.8 to -38.8 | 21.7 | 5.3 to 38.2 | -43.4 | -46.3 to -40.5 | 12.8 | 9.9 to 5.6 |
| LDL_Anadaraja_ | -75.6 | -85.9 to -65.2 | 32.7 | 22.4 to 43.1 | -45.7 | -47.7 to -43.7 | 20.5 | 18.5 to 22.5 | -81 | -93.5 to -68.5 | 33.3 | 20.8 to 45.9 | -49.6 | -52.2 to -47 | 19.1 | 16.6 to 21.7 | -52.7 | -69.6 to -35.8 | 26.3 | 9.4 to 43.2 | -36.1 | -39 to -33.2 | 20.6 | 17.7 to 23.5 |
| LDL_Ahmadi_ | -71.5 | -129.1 to -13.8 | 531.8 | 474.1 to 589.4 | -37.2 | -47.7 to -26.8 | 310 | 299.6 to 320.5 | -72.9 | -142.8 to -2.9 | 566.1 | 496.1 to 636 | -46.4 | -60.4 to -32.4 | 328.1 | 314.1 to 342.1 | -28 | -114.6 to 58.7 | 377.2 | 290.6 to 463.9 | -15.7 | -30.4 to -1 | 272.1 | 257.4 to 286.8 |
| LDL_Puavilai_ | -53.1 | -61.3 to -44.8 | 33.2 | 25 to 41.5 | -30 | -31.7 to -28.3 | 27.3 | 25.5 to 29 | -56.3 | -66 to -46.7 | 32 | 22.3 to 41.6 | -30.9 | -33 to -28.8 | 24.7 | 22.6 to 26.7 | -38.9 | -54.7 to -23.2 | 34.5 | 18.8 to 50.2 | -27.3 | -30.2 to -24.3 | 31 | 28.1 to 34 |
| LDL_Vujovic_ | -42.6 | -50.1 to -35.2 | 35.4 | 27.9 to 42.8 | -24.8 | -26.5 to -23.1 | 30.6 | 29 to 32.3 | -44.8 | -53.5 to -36.2 | 33.8 | 25.2 to 42.4 | -24.9 | -26.9 to -23 | 27.5 | 25.6 to 29.5 | -33.3 | -48.8 to -17.9 | 38.9 | 23.4 to 54.3 | -23.5 | -26.5 to -20.5 | 35.3 | 32.3 to 38.3 |
| LDL_Chen and Zhang_ | -22.9 | -28.6 to -17.1 | 37.2 | 31.5 to 43 | -25.3 | -26.8 to -23.7 | 27.3 | 25.7 to 28.9 | -22.5 | -28.9 to -16.2 | 35.6 | 29.3 to 42 | -23.9 | -25.7 to -22.1 | 23.8 | 22 to 25.5 | -24.2 | -38.6 to -9.9 | 42.9 | 28.5 to 57.2 | -26.8 | -29.9 to -23.8 | 33.1 | 30 to 36.1 |
| LDL_de Cordova_ | -14.9 | -24 to -5.7 | 80.8 | 71.7 to 89.9 | -34.5 | -36.9 to -32.1 | 44.3 | 41.9 to 46.7 | -13.8 | -24.5 to -3.2 | 83.5 | 72.8 to 94.1 | -33.9 | -36.8 to -31 | 43.4 | 40.5 to 46.3 | -17.4 | -36.3 to 1.4 | 70.7 | 51.8 to 89.5 | -35.6 | -39.8 to -31.4 | 46.1 | 41.9 to 50.3 |
| LDL_Martin_ | -23.7 | -30.4 to -17.1 | 46 | 39.3 to 52.6 | -22.8 | -24.4 to -21.2 | 30.1 | 28.5 to 31.7 | -24.7 | -32.3 to -17 | 45.7 | 38 to 53.4 | -22 | -23.8 to -20.2 | 26.8 | 24.9 to 28.6 | -21.2 | -35.9 to -6.5 | 47.6 | 32.9 to 62.3 | -23.4 | -26.4 to -20.3 | 35.5 | 32.5 to 38.5 |
| LDL_Choi_ | -23.1 | -29.6 to -16.7 | 44.7 | 38.2 to 51.2 | -12.2 | -13.8 to -10.5 | 42.2 | 40.6 to 43.9 | -23.9 | -31.2 to -16.6 | 42.9 | 35.6 to 50.2 | -11.6 | -13.5 to -9.7 | 38.6 | 36.7 to 40.5 | -20.2 | -35.2 to -5.1 | 50.3 | 35.2 to 65.3 | -12.1 | -15.2 to -9 | 47.9 | 44.8 to 50.9 |
| **Absolute percentage error** | **Med** | **95% CI** | **95th P** | **95% CI** | **Med** | **95% CI** | **95th P** | **95% CI** | **Med** | **95% CI** | **95th P** | **95% CI** | **Med** | **95% CI** | **95th P** | **95% CI** | **Med** | **95% CI** | **95th P** | **95% CI** | **Med** | **95% CI** | **95th P** | **95% CI** |
| LDL_Friedwald_ | 34.8 | 26.4 to 46.6 | 146.4 | 123.1 to 207.4 | **9.2** | 8.6 to 10 | 35.4 | 31.4 to 42.6 | 36.4 | 26.8 to 52.5 | 169.7 | N/A | 10.2 | 9.5 to 10.8 | 39.3 | 34.1 to 48.8 | 27.7 | 14.7 to 46.4 | 95.6 | N/A | **7.3** | 6.7 to 8.7 | 27.4 | 23.8 to 35.6 |
| LDL_DeLong_ | 20.6 | 13 to 30.1 | 79.6 | 68.6 to 123.2 | **5.6** | 5.1 to 6.1 | 24.2 | 20.6 to 29.5 | 22.9 | 14.3 to 35.2 | 79.1 | N/A | 5.5 | 4.8 to 6.4 | 23.7 | 19.9 to 29.4 | **10.6** | 7.9 to 23.5 | 75.5 | N/A | **5.7** | 5.1 to 6.5 | 26.2 | 20.1 to 33.4 |
| LDL_Rao_ | 19.7 | 12.9 to 24.2 | 90.5 | 57.6 to 144.4 | **6.2** | 5.6 to 6.6 | 22.4 | 20.2 to 28.4 | 21.5 | 12.5 to 29.6 | 90.2 | N/A | 6.2 | 5.6 to 6.8 | 21.0 | 19.8 to 28.2 | 19.2 | 7.5 to 24.2 | 78.8 | N/A | **6.1** | 5.3 to 6.8 | 24.7 | 19.2 to 30.2 |
| LDL_Hattori_ | 39.7 | 31.3 to 51.8 | 148.5 | 123.1 to 205.6 | 14.7 | 13.9 to 15.4 | 40.1 | 36.2 to 45.8 | 41.9 | 32.2 to 56.2 | 170.8 | N/A | 15.7 | 14.9 to 16.3 | 42.4 | 38.2 to 53.1 | 32.3 | 20.5 to 51.2 | 99.5 | N/A | 12.5 | 11.2 to 13.5 | 31.9 | 28.1 to 40.9 |
| LDL_Anadaraja_ | 35.0 | 23.7 to 44.7 | 130.9 | 102.8 to 219.6 | **10.0** | 9.4 to 10.6 | 35.4 | 30.6 to 42.6 | 37.2 | 23.2 to 53.5 | 155.3 | N/A | 11.4 | 10.4 to 12.7 | 38.7 | 33.4 to 49.6 | 28.3 | 17.5 to 43.6 | 106.3 | N/A | **8.0** | 6.9 to 9 | 27.0 | 23.7 to 35.1 |
| LDL_Ahmadi_ | 339.1 | 297.5 to 424.2 | 1199.0 | 873.8 to 1610.3 | 92.9 | 89.4 to 96.6 | 313.9 | 279.1 to 343.8 | 405.1 | 324.8 to 459.9 | 1282.6 | N/A | 95.3 | 89.8 to 100 | 322.8 | 286.5 to 387.5 | 253.0 | 199.8 to 312.1 | 1044.1 | N/A | 89.9 | 82.3 to 94.7 | 280.3 | 233 to 350.7 |
| LDL_Puavilai_ | 21.0 | 14.6 to 28.4 | 92.3 | 77 to 136.7 | **5.7** | 5.3 to 6.1 | 24.8 | 21.9 to 30.2 | 23.0 | 15.5 to 36.6 | 91.3 | N/A | 5.8 | 5.2 to 6.3 | 24.0 | 21.9 to 31.5 | 14.8 | 7.4 to 26.3 | 75.7 | N/A | **5.6** | 5 to 6.5 | 25.5 | 19.5 to 32.6 |
| LDL_Vujovic_ | 17.7 | 10.3 to 26.4 | 67.8 | 55.9 to 114.1 | **5.7** | 5.1 to 6.2 | 24.3 | 21 to 28.2 | 23.0 | 14.8 to 31.2 | 67.4 | N/A | 5.2 | 4.6 to 5.9 | 21.7 | 19.1 to 28.1 | **7.5** | 5.4 to 17.1 | 80.6 | N/A | **6.6** | 5.5 to 7.4 | 26.5 | 21.6 to 34 |
| LDL_Chen and Zhang_ | 18.0 | 11.8 to 21.3 | 69.6 | 54.8 to 113.2 | **5.3** | 4.9 to 5.8 | 21.1 | 19.2 to 26.2 | 18.6 | 15 to 21.7 | 69.5 | N/A | 5.2 | 4.6 to 5.8 | 20.0 | 17.3 to 24.4 | **7.6** | 5.2 to 25.8 | 88.8 | N/A | **5.4** | 4.8 to 6.3 | 24.8 | 19.7 to 37.4 |
| LDL_de Cordova_ | 55.6 | 41.4 to 68.1 | 186.0 | 123.5 to 247.5 | **6.9** | 6.4 to 7.6 | 42.1 | 32.9 to 48.4 | 60.2 | 45.6 to 68.5 | 195.6 | N/A | 6.6 | 5.9 to 7.3 | 41.4 | 31.8 to 48.3 | 31.8 | 20.5 to 70.1 | 172.3 | N/A | **7.6** | 6.6 to 8.9 | 44.1 | 30.5 to 60.8 |
| LDL_Martin_ | 22.7 | 18.4 to 32.6 | 81.6 | 59.8 to 165 | **5.2** | 4.8 to 5.6 | 24.6 | 20.9 to 29.1 | 26.5 | 19.9 to 34.6 | 81.5 | N/A | 4.9 | 4.4 to 5.4 | 23.0 | 18.3 to 26.9 | 13.2 | 4.4 to 38.1 | 97.6 | N/A | **5.9** | 5.1 to 6.7 | 30.5 | 21 to 42.4 |
| LDL_Choi_ | 21.0 | 17.4 to 28.1 | 78.5 | 58.4 to 128.7 | **11.6** | 10.8 to 12.2 | 32.0 | 28.4 to 37.8 | 25.4 | 17.5 to 29.8 | 78.5 | N/A | 10.0 | 9.2 to 11.4 | 28.8 | 25.3 to 36.4 | 20.0 | 12.7 to 29.6 | 103.5 | N/A | 13.8 | 12.5 to 14.8 | 36.0 | 30.8 to 48.6 |

*95^th^ P* 95th percentile, *Med* Median, *N/A* Not available because of limited numbers of test results

Top three equations are presented in color: red represents the lowest, orange represents the second, and yellow represents the third lowest absolute percentage error.

**Supplementary Table S5.** Intraclass correlation coefficient and systemic differences among the 12 equations in comparison with directly measured LDL according to subgroup by triglyceride (TG) concentration

|  | **Population 1** | | | | | | | | | | | | | | | | | |
| --- | --- | --- | --- | --- | --- | --- | --- | --- | --- | --- | --- | --- | --- | --- | --- | --- | --- | --- |
|  | **Total** | | | | | | **Men** | | | | | | **Women** | | | | | |
|  | **TG group 1 (TG < 175 mg/dL, Test n=3566)** | | **TG group 2 (TG 175-400 mg/dL, Test n=1330)** | | **TG group 3 (TG > 400 mg/dL, Test n=302)** | | **TG group 1 (TG < 175 mg/dL, Test n=1517)** | | **TG group 2 (TG 175-400 mg/dL, Test n=819)** | | **TG group 3 (TG > 400 mg/dL, Test n=234)** | | **TG group 1 (TG < 175 mg/dL, Test n=2049)** | | **TG group 2 (TG 175-400 mg/dL, Test n=511)** | | **TG group 3 (TG > 400 mg/dL, Test n=68)** | |
| **Intraclass correlation** | **Coefficient** | **95%CI** | **Coefficient** | **95%CI** | **Coefficient** | **95%CI** | **Coefficient** | **95%CI** | **Coefficient** | **95%CI** | **Coefficient** | **95%CI** | **Coefficient** | **95%CI** | **Coefficient** | **95%CI** | **Coefficient** | **95%CI** |
| LDL_Friedwald_ | 0.91 | -0.01 to 0.98 | 0.83 | -0.03 to 0.96 | 0.55 | -0.07 to 0.84 | 0.91 | -0.01 to 0.98 | 0.82 | -0.04 to 0.95 | 0.54 | -0.07 to 0.83 | 0.91 | -0.01 to 0.98 | 0.84 | -0.03 to 0.96 | 0.59 | -0.06 to 0.87 |
| LDL_DeLong_ | 0.95 | 0.16 to 0.99 | 0.92 | 0.05 to 0.98 | 0.77 | -0.01 to 0.92 | 0.95 | 0.21 to 0.99 | 0.92 | 0.03 to 0.98 | 0.76 | -0.01 to 0.92 | 0.95 | 0.12 to 0.99 | 0.93 | 0.06 to 0.98 | 0.80 | -0.03 to 0.94 |
| LDL_Rao_ | 0.92 | 0.01 to 0.98 | 0.90 | -0.02 to 0.97 | 0.07 | -0.05 to 0.18 | 0.92 | 0.01 to 0.98 | 0.89 | -0.02 to 0.97 | 0.03 | -0.1 to 0.16 | 0.92 | 0 to 0.98 | 0.9 | -0.02 to 0.98 | 0.85 | 0.51 to 9393 |
| LDL_Hattori_ | 0.83 | -0.03 to 0.96 | 0.75 | -0.03 to 0.93 | 0.50 | -0.06 to 0.82 | 0.83 | -0.03 to 0.96 | 0.74 | -0.03 to 0.93 | 0.49 | -0.06 to 0.81 | 0.83 | -0.03 to 0.96 | 0.76 | -0.03 to 0.94 | 0.53 | -0.05 to 0.84 |
| LDL_Anadaraja_ | 0.92 | 0.91 to 0.92 | 0.84 | 0.01 to 0.95 | 0.54 | -0.07 to 0.83 | 0.93 | 0.91 to 0.94 | 0.83 | -0.03 to 0.95 | 0.53 | -0.07 to 0.83 | 0.91 | 0.9 to 0.93 | 0.86 | 0.1 to 0.96 | 0.56 | -0.06 to 0.85 |
| LDL_Ahmadi_ | 0.80 | 0.78 to 0.81 | 0.22 | -0.07 to 0.54 | -0.02 | -0.06 to 0.04 | 0.79 | 0.76 to 0.82 | 0.21 | -0.07 to 0.53 | -0.02 | -0.08 to 0.04 | 0.80 | 0.79 to 0.82 | 0.24 | -0.07 to 0.56 | 0.01 | -0.02 to 0.06 |
| LDL_Puavilai_ | 0.94 | 0.11 to 0.98 | 0.91 | 0.01 to 0.98 | 0.74 | -0.06 to 0.92 | 0.94 | 0.15 to 0.98 | 0.91 | 0 to 0.98 | 0.73 | -0.06 to 0.91 | 0.94 | 0.08 to 0.98 | 0.92 | 0.01 to 0.98 | 0.76 | -0.06 to 0.93 |
| LDL_Vujovic_ | 0.95 | 0.25 to 0.99 | 0.94 | 0.15 to 0.98 | 0.81 | 0.16 to 0.93 | 0.95 | 0.33 to 0.99 | 0.94 | 0.13 to 0.98 | 0.80 | 0.17 to 0.92 | 0.95 | 0.2 to 0.99 | 0.94 | 0.17 to 0.99 | 0.84 | 0.07 to 0.95 |
| LDL_Chen and Zhang_ | 0.88 | -0.02 to 0.97 | 0.92 | 0.07 to 0.98 | 0.80 | 0.73 to 0.85 | 0.89 | -0.01 to 0.97 | 0.92 | 0.08 to 0.98 | 0.78 | 0.71 to 8287 | 0.88 | -0.03 to 0.97 | 0.92 | 0.06 to 0.98 | 0.89 | 0.68 to 9520 |
| LDL_de Cordova_ | 0.75 | -0.06 to 0.92 | 0.89 | 0.61 to 0.95 | 0.34 | 0.16 to 0.48 | 0.76 | -0.06 to 0.93 | 0.90 | 0.68 to 0.95 | 0.27 | 0.11 to 0.41 | 0.73 | -0.06 to 0.92 | 0.88 | 0.47 to 0.95 | 0.74 | 0.43 to 0.87 |
| LDL_Martin_ | 0.92 | 0.01 to 0.98 | 0.94 | 0.3 to 0.98 | 0.87 | 0.82 to 0.91 | 0.92 | 0.02 to 0.98 | 0.94 | 0.32 to 0.98 | 0.86 | 0.81 to 0.9 | 0.91 | 0 to 0.98 | 0.94 | 0.26 to 0.98 | 0.91 | 0.82 to 0.95 |
| LDL_Choi_ | 0.99 | 0.99 to 0.99 | 0.99 | 0.98 to 0.99 | 0.88 | 0.85 to 0.9 | 0.99 | 0.99 to 0.99 | 0.98 | 0.98 to 0.99 | 0.86 | 0.82 to 0.89 | 0.99 | 0.99 to 0.99 | 0.99 | 0.98 to 0.99 | 0.93 | 0.89 to 0.96 |
| **Systemic differences** | **Mean (SD)** | **95%CI** | **Mean (SD)** | **95%CI** | **Mean (SD)** | **95%CI** | **Mean (SD)** | **95%CI** | **Mean (SD)** | **95%CI** | **Mean (SD)** | **95%CI** | **Mean (SD)** | **95%CI** | **Mean (SD)** | **95%CI** | **Mean (SD)** | **95%CI** |
| LDL_Friedwald_ | -14.6 (6.2) | -14.8 to -14.3 | -23.8 (8.3) | -24.3 to -23.3 | -50 (22.1) | -52.5 to -47.4 | -14.5 (6.4) | -14.8 to -14.1 | -24.4 (8.6) | -25 to -23.8 | -51.0 (23.2) | -54 to -48 | -14.7 (6) | -15 to -14.4 | -22.8 (7.8) | -23.5 to -22.1 | -46.4 (17.8) | -50.7 to -42 |
| LDL_DeLong_ | -10.3 (6.0) | -10.5 to -10.1 | -14.1 (7.3) | -14.5 to -13.6 | -25.9 (17.9) | -27.9 to -23.8 | -10 (6.2) | -10.3 to -9.7 | -14.5 (7.4) | -15 to -14 | -26.1 (18.5) | -28.5 to -23.7 | -10.6 (5.8) | -10.8 to -10.3 | -13.4 (7) | -14 to -12.7 | -25 (15.6) | -28.8 to -21.2 |
| LDL_Rao_ | -13.5 (6.0) | -13.7 to -13.3 | -17.7 (7) | -18 to -17.2 | 1.5 (129) | -13.1 to 16.1 | -13.3 (6.3) | -13.6 to -12.9 | -18 (7.1) | -18.5 to -17.5 | 6.2 (146) | -12.6 to 25 | -13.7 (5.8) | -14 to -13.4 | -17.1 (6.8) | -17.7 to -16.4 | -14.6 (17.8) | -18.9 to -10.3 |
| LDL_Hattori_ | -20.7 (6.8) | -20.9 to -20.4 | -30 (8.5) | -30.5 to -29.5 | -54.9 (21.1) | -57.3 to -52.5 | -20.3 (7.1) | -20.7 to -19.9 | -30.4 (8.8) | -31 to -29.8 | -55.8 (22.1) | -58.7 to -52.9 | -20.9 (6.6) | -21.2 to -20.6 | -29.3 (8) | -30 to -28.5 | -51.8 (17.2) | -56 to -47.6 |
| LDL_Anadaraja_ | 0.3 (14.2) | -0.1 to 0.8 | -19.5 (12.4) | -20.1 to -18.8 | -49.9 (21.6) | -52.3 to -47.4 | -3.5 (12.9) | -4.2 to -2.9 | -20.8 (12.1) | -21.6 to -19.9 | -50.5 (22.4) | -53.4 to -47.6 | 3.2 (14.4) | 2.6 to 3.8 | -17.3 (12.5) | -18.4 to -16.2 | -47.8 (18.9) | -52.3 to -43.1 |
| LDL_Ahmadi_ | 1.5 (24.2) | 0.7 to 2.2 | 88.8 (37.8) | 86.7 to 90.8 | 317.5 (234.3) | 291 to 344 | 5.8 (23.6) | 4.6 to 7 | 92.3 (38.3) | 89.6 to 94.9 | 330.9 (260.4) | 297.3 to 364.4 | -1.8 (24.1) | -2.8 to -0.7 | 83.1 (36.4) | 80 to 86.3 | 271.5 (90.1) | 249.7 to 293.3 |
| LDL_Puavilai_ | -11 (6.0) | -11.2 to -10.7 | -15.6 (7.4) | -16 to -15.2 | -29.8 (17.9) | -31.8 to -27.7 | -10.7 (6.2) | -11 to -10.3 | -16.1 (7.6) | -16.6 to -15.6 | -30.2 (18.5) | -32.6 to -27.8 | -11.2 (5.8) | -11.4 to -10.9 | -14.9 (7) | -15.5 to -14.2 | -28.5 (15.8) | -32.3 to -24.6 |
| LDL_Vujovic_ | -9.5 (5.9) | -9.7 to -9.3 | -12.1 (7.1) | -12.5 to -11.7 | -21 (18.2) | -23.1 to -18.9 | -9.1 (6.2) | -9.4 to -8.8 | -12.5 (7.3) | -13 to -12 | -21.1 (19) | -23.6 to -18.6 | -9.8 (5.8) | -10 to -9.5 | -11.6 (6.9) | -12.2 to -10.9 | -20.8 (15.4) | -24.5 to -17 |
| LDL_Chen and Zhang_ | -15.8 (7.3) | -16.1 to -15.5 | -13.8 (7.9) | -14.2 to -13.3 | -8 (24.2) | -10.7 to -5.2 | -15 (7.4) | -15.4 to -14.6 | -13.7 (7.9) | -14.2 to -13.1 | -7.1 (26.2) | -10.5 to -3.7 | -16.5 (7.1) | -16.8 to -16.1 | -14 (7.8) | -14.6 to -13.2 | -10.9 (15.3) | -14.7 to -7.2 |
| LDL_de Cordova_ | -23 (11.4) | -23.3 to -22.5 | -10.8 (12.9) | -11.5 to -10.1 | 25.1 (48.8) | 19.6 to 30.7 | -21.3 (11.4) | -21.8 to -20.6 | -9.8 (12.8) | -10.7 to -9 | 27.9 (53.8) | 20.9 to 34.8 | -24.2 (11.3) | -24.7 to -23.7 | -12.3 (12.9) | -13.5 to -11.2 | 15.7 (23.1) | 10.1 to 21.3 |
| LDL_Martin_ | -13.6 (6.3) | -13.8 to -13.4 | -11 (7.8) | -11.4 to -10.5 | -6.5 (18.7) | -8.6 to -4.4 | -13 (6.5) | -13.3 to -12.6 | -10.9 (7.9) | -11.4 to -10.3 | -6.4 (19.6) | -8.9 to -3.8 | -14.1 (6.1) | -14.3 to -13.7 | -11.2 (7.6) | -11.9 to -10.5 | -7.1 (15.5) | -10.8 to -3.3 |
| LDL_Choi_ | 0.5 (5.4) | 0.3 to 0.6 | -0.7 (6.7) | -1.1 to -0.4 | -2.8 (20.9) | -5.2 to -0.5 | 0.3 (5.7) | 0 to 0.6 | -1.2 (6.8) | -1.7 to -0.7 | -2.5 (22.4) | -5.4 to 0.4 | 0.6 (5.3) | 0.3 to 0.8 | 0 (6.5) | -0.6 to 0.5 | -4.1 (14.9) | -7.7 to -0.5 |

(*continue*)

|  | **Population 2** | | | | | | | | | | | | | | | | | |
| --- | --- | --- | --- | --- | --- | --- | --- | --- | --- | --- | --- | --- | --- | --- | --- | --- | --- | --- |
|  | **Total** | | | | | | **Men** | | | | | | **Women** | | | | | |
|  | **TG group 1 (TG < 175 mg/dL, Test n=1546)** | | **TG group 2 (TG 175-400 mg/dL, Test n=542)** | | **TG group 3 (TG > 400 mg/dL, Test n=75)** | | **TG group 1 (TG < 175 mg/dL, Test n=669)** | | **TG group 2 (TG 175-400 mg/dL, Test n=336)** | | **TG group 3 (TG > 400 mg/dL, Test n=57)** | | **TG group 1 (TG < 175 mg/dL, Test n=877)** | | **TG group 2 (TG 175-400 mg/dL, Test n=206)** | | **TG group 3 (TG > 400 mg/dL, Test n=18)** | |
| **Intraclass correlation** | **Coefficient** | **95%CI** | **Coefficient** | **95%CI** | **Coefficient** | **95%CI** | **Coefficient** | **95%CI** | **Coefficient** | **95%CI** | **Coefficient** | **95%CI** | **Coefficient** | **95%CI** | **Coefficient** | **95%CI** | **Coefficient** | **95%CI** |
| LDL_Friedwald_ | 0.92 | -0.01 to 0.98 | 0.86 | -0.03 to 0.96 | 0.5 | -0.03 to 0.76 | 0.92 | -0.01 to 0.98 | 0.87 | -0.03 to 0.97 | 0.52 | -0.07 to 0.79 | 0.91 | -0.01 to 0.98 | 0.84 | -0.03 to 0.96 | 0.46 | -0.04 to 0.77 |
| LDL_DeLong_ | 0.95 | 0.12 to 0.99 | 0.94 | 0.17 to 0.98 | 0.76 | 0.29 to 0.89 | 0.95 | 0.16 to 0.99 | 0.94 | 0.16 to 0.99 | 0.75 | 0.27 to 0.89 | 0.94 | 0.09 to 0.99 | 0.93 | 0.16 to 0.98 | 0.75 | 0.22 to 0.92 |
| LDL_Rao_ | 0.92 | 0 to 0.98 | 0.92 | 0.01 to 0.98 | -0.02 | -0.25 to 0.2 | 0.93 | 0.01 to 0.98 | 0.92 | 0.01 to 0.98 | 0.15 | -0.12 to 0.39 | 0.92 | 0 to 0.98 | 0.90 | -0.01 to 0.97 | -0.09 | -0.53 to 0.39 |
| LDL_Hattori_ | 0.84 | -0.03 to 0.96 | 0.79 | -0.03 to 0.94 | 0.47 | -0.08 to 0.75 | 0.85 | -0.03 to 0.96 | 0.80 | -0.03 to 0.95 | 0.48 | -0.09 to 0.78 | 0.84 | -0.03 to 0.96 | 0.76 | -0.03 to 0.94 | 0.44 | -0.08 to 0.76 |
| LDL_Anadaraja_ | 0.93 | 0.93 to 0.94 | 0.88 | 0.08 to 0.96 | 0.51 | -0.06 to 0.78 | 0.94 | 0.92 to 0.95 | 0.88 | 0.02 to 0.97 | 0.51 | -0.08 to 0.79 | 0.93 | 0.91 to 0.94 | 0.88 | 0.22 to 0.96 | 0.50 | -0.06 to 0.8 |
| LDL_Ahmadi_ | 0.80 | 0.78 to 0.82 | 0.22 | -0.07 to 0.54 | -0.03 | -0.13 to 0.1 | 0.80 | 0.76 to 0.84 | 0.23 | -0.06 to 0.56 | -0.02 | -0.1 to 0.09 | 0.80 | 0.77 to 0.82 | 0.20 | -0.06 to 0.52 | -0.04 | -0.3 to 0.31 |
| LDL_Puavilai_ | 0.94 | 0.08 to 0.99 | 0.93 | 0.07 to 0.98 | 0.71 | 0.18 to 0.88 | 0.95 | 0.11 to 0.99 | 0.93 | 0.07 to 0.98 | 0.71 | 0.16 to 0.88 | 0.94 | 0.06 to 0.98 | 0.92 | 0.06 to 0.98 | 0.69 | 0.14 to 0.89 |
| LDL_Vujovic_ | 0.95 | 0.2 to 0.99 | 0.95 | 0.38 to 0.99 | 0.81 | 0.47 to 0.91 | 0.96 | 0.27 to 0.99 | 0.96 | 0.37 to 0.99 | 0.80 | 0.45 to 0.91 | 0.95 | 0.15 to 0.99 | 0.95 | 0.38 to 0.98 | 0.83 | 0.37 to 0.94 |
| LDL_Chen and Zhang_ | 0.89 | -0.01 to 0.97 | 0.93 | 0.25 to 9798 | 0.89 | 0.83 to 9272 | 0.90 | 0 to 0.97 | 0.94 | 0.28 to 0.98 | 0.90 | 0.83 to 0.94 | 0.88 | -0.02 to 0.97 | 0.92 | 0.19 to 0.97 | 0.82 | 0.58 to 0.93 |
| LDL_de Cordova_ | 0.76 | -0.06 to 0.93 | 0.90 | 0.74 to 0.95 | 0.21 | -0.01 to 0.41 | 0.79 | -0.05 to 0.94 | 0.91 | 0.8 to 0.95 | 0.37 | 0 to 0.63 | 0.74 | -0.06 to 0.92 | 0.87 | 0.6 to 0.94 | -0.11 | -0.46 to 0.32 |
| LDL_Martin_ | 0.92 | 0.01 to 0.98 | 0.95 | 0.63 to 0.98 | 0.85 | 0.77 to 0.9 | 0.93 | 0.03 to 0.98 | 0.96 | 0.67 to 0.99 | 0.83 | 0.73 to 0.9 | 0.91 | 0 to 0.98 | 0.94 | 0.54 to 0.98 | 0.87 | 0.69 to 0.95 |
| LDL_Choi_ | 0.99 | 0.99 to 0.99 | 0.99 | 0.98 to 0.99 | 0.91 | 0.87 to 0.94 | 0.99 | 0.99 to 0.99 | 0.99 | 0.98 to 0.99 | 0.90 | 0.83 to 0.94 | 0.99 | 0.99 to 0.99 | 0.98 | 0.97 to 0.99 | 0.94 | 0.85 to 0.98 |
| **Systemic differences** | **Mean (SD)** | **95%CI** | **Mean (SD)** | **95%CI** | **Mean (SD)** | **95%CI** | **Mean (SD)** | **95%CI** | **Mean (SD)** | **95%CI** | **Mean (SD)** | **95%CI** | **Mean (SD)** | **95%CI** | **Mean (SD)** | **95%CI** | **Mean (SD)** | **95%CI** |
| LDL_Friedwald_ | -15.2 (5.8) | -15.4 to -14.8 | -22.1 (8.1) | -22.8 to -21.4 | -49.7 (45.2) | -60.1 to -39.3 | -14.7 (5.7) | -15.1 to -14.2 | -22.7 (8.3) | -23.6 to -21.8 | -47.4 (38.2) | -57.6 to -37.2 | -15.5 (5.9) | -15.9 to -15.1 | -21.2 (7.7) | -22.2 to -20.1 | -57.1 (63.3) | -88.5 to -25.5 |
| LDL_DeLong_ | -10.9 (5.8) | -11.2 to -10.6 | -12.4 (7.5) | -13.1 to -11.8 | -23.5 (26.7) | -29.6 to -17.3 | -10.2 (5.6) | -10.6 to -9.8 | -12.8 (7.5) | -13.6 to -12 | -22.7 (26) | -29.6 to -15.8 | -11.5 (5.9) | -11.9 to -11 | -11.8 (7.4) | -12.8 to -10.8 | -25.8 (29.6) | -40.6 to -11 |
| LDL_Rao_ | -14.1 (5.7) | -14.4 to -13.8 | -16.1 (7.4) | -16.7 to -15.4 | 27.4 (214.5) | -22 to 76.8 | -13.5 (5.6) | -13.9 to -13 | -16.3 (7.4) | -17.1 to -15.5 | 7 (87.1) | -16.1 to 30.1 | -14.5 (5.8) | -14.9 to -14.1 | -15.6 (7.4) | -16.6 to -14.6 | 92.1 (411.7) | -112.7 to 296.7 |
| LDL_Hattori_ | -21.1 (6.8) | -21.4 to -20.7 | -28.4 (8.3) | -29.1 to -27.6 | -54.2 (43) | -64.1 to -44.3 | -20.5 (6.6) | -21 to -20 | -28.8 (8.5) | -29.7 to -27.9 | -51.6 (36.2) | -61.2 to -42 | -21.6 (6.9) | -22 to -21.1 | -27.6 (8) | -28.7 to -26.4 | -62.6 (60.3) | -92.5 to -32.5 |
| LDL_Anadaraja_ | 0.6 (13.7) | -0.1 to 1.3 | -17.2 (11.6) | -18.2 to -16.2 | -49.5 (40.4) | -58.8 to -40.1 | -3.1 (12.5) | -4 to -2.2 | -18.8 (11.1) | -20 to -17.6 | -47.8 (36) | -57.3 to -38.1 | 3.4 (14) | 2.4 to 4.3 | -14.6 (11.8) | -16.2 to -12.9 | -55 (52.8) | -81.3 to -28.7 |
| LDL_Ahmadi_ | 0.9 (25.3) | -0.4 to 2.1 | 89.8 (36.9) | 86.7 to 92.9 | 356.5 (343.7) | 277.4 to 435.5 | 6.2 (24.6) | 4.3 to 8.1 | 93.6 (38.3) | 89.5 to 97.7 | 331.7 (229) | 271 to 392.4 | -3.2 (25.2) | -4.9 to -1.5 | 83.6 (33.8) | 78.9 to 88.2 | 434.9 (576.9) | 148.1 to 721.8 |
| LDL_Puavilai_ | -11.5 (5.8) | -11.8 to -11.2 | -14 (7.5) | -14.6 to -13.3 | -27.8 (29.6) | -34.6 to -20.9 | -10.9 (5.6) | -11.3 to -10.4 | -14.4 (7.6) | -15.2 to -13.5 | -26.8 (27.9) | -34.2 to -19.3 | -12.1 (5.8) | -12.5 to -11.6 | -13.3 (7.4) | -14.3 to -12.3 | -31 (35) | -48.4 to -13.5 |
| LDL_Vujovic_ | -10.1 (5.8) | -10.4 to -9.8 | -10.5 (7.4) | -11.1 to -9.9 | -18.3 (23.6) | -23.7 to -12.8 | -9.3 (5.6) | -9.7 to -8.9 | -10.8 (7.4) | -11.6 to -10 | -17.9 (23.8) | -24.2 to -11.5 | -10.7 (5.9) | -11 to -10.2 | -10 (7.4) | -11 to -8.9 | -19.5 (23.5) | -31.2 to -7.8 |
| LDL_Chen and Zhang_ | -16.2 (7.8) | -16.6 to -15.8 | -12.2 (8.5) | -13 to -11.5 | -2.6 (19.1) | -7 to 1.8 | -15 (7.4) | -15.6 to -14.4 | -12.2 (8.5) | -13.1 to -11.2 | -3.1 (17.5) | -7.8 to 1.5 | -17.2 (7.9) | -17.7 to -16.6 | -12.3 (8.6) | -13.5 to -11.1 | -0.9 (23.8) | -12.8 to 10.9 |
| LDL_de Cordova_ | -23.1 (12.5) | -23.7 to -22.4 | -9.4 (14) | -10.6 to -8.2 | 35.5 (56.8) | 22.4 to 48.6 | -21.1 (12.1) | -22 to -20.1 | -8.6 (14.3) | -10.1 to -7.1 | 32.7 (38) | 22.6 to 42.8 | -24.6 (12.6) | -25.4 to -23.7 | -10.6 (13.5) | -12.5 to -8.8 | 44.4 (95.7) | -3.1 to 92 |
| LDL_Martin_ | -14.2 (6.4) | -14.5 to -13.8 | -9.2 (8.5) | -9.9 to -8.5 | -3 (24.1) | -8.5 to 2.6 | -13.2 (6.2) | -13.6 to -12.7 | -9.1 (8.5) | -10 to -8.1 | -1.5 (24.4) | -8 to 5 | -14.9 (6.6) | -15.4 to -14.5 | -9.4 (8.5) | -10.6 to -8.2 | -7.7 (23.2) | -19.2 to 3.9 |
| LDL_Choi_ | 0.0 (5.1) | -0.3 to 0.2 | 1.0 (7) | 0.4 to 1.5 | 1.0 (18.2) | -3.2 to 5.2 | 0.2 (5) | -0.2 to 0.6 | 0.6 (6.9) | -0.2 to 1.3 | 0.3 (19.3) | -4.8 to 5.4 | -0.3 (5.1) | -0.6 to 0.1 | 1.6 (7) | 0.6 to 2.5 | 3 (14.8) | -4.4 to 10.4 |

(*continue*)

|  | **Population 3** | | | | | | | | | | | | | | | | | |
| --- | --- | --- | --- | --- | --- | --- | --- | --- | --- | --- | --- | --- | --- | --- | --- | --- | --- | --- |
|  | **Total** | | | | | | **Men** | | | | | | **Women** | | | | | |
|  | **TG group 1 (TG < 175 mg/dL, Test n=0)** | | **TG group 2 (TG 175-400 mg/dL, Test n=758)** | | **TG group 3 (TG > 400 mg/dL, Test n=131)** | | **TG group 1 (TG < 175 mg/dL, Test n=0)** | | **TG group 2 (TG 175-400 mg/dL, Test n=336)** | | **TG group 3 (TG > 400 mg/dL, Test n=99)** | | **TG group 1 (TG < 175 mg/dL, Test n=0)** | | **TG group 2 (TG 175-400 mg/dL, Test n=206)** | | **TG group 3 (TG > 400 mg/dL, Test n=32)** | |
| **Intraclass correlation** | **Coefficient** | **95%CI** | **Coefficient** | **95%CI** | **Coefficient** | **95%CI** | **Coefficient** | **95%CI** | **Coefficient** | **95%CI** | **Coefficient** | **95%CI** | **Coefficient** | **95%CI** | **Coefficient** | **95%CI** | **Coefficient** | **95%CI** |
| LDL_Friedwald_ | N/A | N/A | 0.92 | 0.71 to 0.97 | 0.60 | 0.09 to 0.80 | N/A | N/A | 0.91 | 0.57 to 0.96 | 0.51 | -0.03 to 0.76 | N/A | N/A | 0.94 | 0.87 to 0.97 | 0.77 | 0.51 to 0.89 |
| LDL_DeLong_ | N/A | N/A | 0.95 | 0.95 to 0.96 | 0.78 | 0.70 to 0.84 | N/A | N/A | 0.95 | 0.95 to 0.96 | 0.75 | 0.6 to 0.84 | N/A | N/A | 0.95 | 0.93 to 0.96 | 0.80 | 0.64 to 0.9 |
| LDL_Rao_ | N/A | N/A | 0.95 | 0.94 to 0.96 | 0.66 | 0.53 to 0.76 | N/A | N/A | 0.95 | 0.93 to 0.96 | 0.63 | 0.48 to 0.74 | N/A | N/A | 0.96 | 0.94 to 0.96 | 0.69 | 0.44 to 0.83 |
| LDL_Hattori_ | N/A | N/A | 0.86 | 0.05 to 0.96 | 0.56 | -0.03 to 0.80 | N/A | N/A | 0.84 | -0.01 to 0.95 | 0.47 | -0.08 to 0.75 | N/A | N/A | 0.89 | 0.23 to 0.96 | 0.74 | 0.29 to 0.89 |
| LDL_Anadaraja_ | N/A | N/A | 0.90 | 0.66 to 0.96 | 0.60 | 0.04 to 0.82 | N/A | N/A | 0.88 | 0.47 to 0.95 | 0.50 | -0.05 to 0.76 | N/A | N/A | 0.94 | 0.86 to 0.96 | 0.80 | 0.43 to 0.92 |
| LDL_Ahmadi_ | N/A | N/A | 0.13 | -0.04 to 0.41 | 0.01 | -0.02 to 0.04 | N/A | N/A | 0.13 | -0.04 to 0.4 | 0.00 | -0.03 to 0.05 | N/A | N/A | 0.14 | -0.04 to 0.43 | 0.01 | -0.03 to 0.09 |
| LDL_Puavilai_ | N/A | N/A | 0.95 | 0.95 to 0.96 | 0.76 | 0.64 to 0.84 | N/A | N/A | 0.95 | 0.94 to 0.96 | 0.71 | 0.48 to 0.83 | N/A | N/A | 0.95 | 0.94 to 0.96 | 0.81 | 0.64 to 0.9 |
| LDL_Vujovic_ | N/A | N/A | 0.95 | 0.94 to 0.96 | 0.80 | 0.72 to 0.85 | N/A | N/A | 0.95 | 0.94 to 0.96 | 0.78 | 0.69 to 0.85 | N/A | N/A | 0.95 | 0.9 to 0.97 | 0.79 | 0.62 to 0.89 |
| LDL_Chen and Zhang_ | N/A | N/A | 0.95 | 0.95 to 0.96 | 0.80 | 0.68 to 0.87 | N/A | N/A | 0.95 | 0.95 to 0.96 | 0.82 | 0.7 to 0.89 | N/A | N/A | 0.95 | 0.94 to 0.96 | 0.75 | 0.48 to 0.88 |
| LDL_de Cordova_ | N/A | N/A | 0.92 | 0.9 to 0.93 | 0.42 | -0.10 to 0.72 | N/A | N/A | 0.92 | 0.91 to 0.93 | 0.37 | -0.1 to 0.69 | N/A | N/A | 0.91 | 0.88 to 0.93 | 0.48 | -0.07 to 0.78 |
| LDL_Martin_ | N/A | N/A | 0.95 | 0.94 to 0.96 | 0.77 | 0.60 to 0.86 | N/A | N/A | 0.95 | 0.94 to 0.96 | 0.76 | 0.62 to 0.85 | N/A | N/A | 0.94 | 0.91 to 0.96 | 0.74 | 0.37 to 0.88 |
| LDL_Choi_ | N/A | N/A | 0.89 | 0.2 to 0.96 | 0.76 | 0.54 to 0.87 | N/A | N/A | 0.90 | 0.28 to 0.96 | 0.78 | 0.59 to 0.87 | N/A | N/A | 0.87 | 0.08 to 0.96 | 0.71 | 0.29 to 0.87 |
| **Systemic differences** | **Mean (SD)** | **95%CI** | **Mean (SD)** | **95%CI** | **Mean (SD)** | **95%CI** | **Mean (SD)** | **95%CI** | **Mean (SD)** | **95%CI** | **Mean (SD)** | **95%CI** | **Mean (SD)** | **95%CI** | **Mean (SD)** | **95%CI** | **Mean (SD)** | **95%CI** |
| LDL_Friedwald_ | N/A | N/A | -9.4 (11.7) | -10.2 to -8.6 | -31 (33) | -36.7 to -25.3 | N/A | N/A | -10.8 (11.4) | -11.8 to -9.8 | -35.3 (32.3) | -41.7 to -28.8 | N/A | N/A | -6.8 (11.8) | -8.2 to -5.4 | -17.8 (31.9) | -29.3 to -6.3 |
| LDL_DeLong_ | N/A | N/A | 1.1 (11.3) | 0.3 to 1.9 | -7.3 (27.9) | -12.1 to -2.5 | N/A | N/A | -0.2 (11) | -1.2 to 0.8 | -10.9 (25.4) | -15.9 to -5.8 | N/A | N/A | 3.4 (11.6) | 2 to 0.8 | 3.8 (32.6) | -8.0 to 15.5 |
| LDL_Rao_ | N/A | N/A | -2.3 (11.3) | -3.1 to 1.5 | 11.6 (34.8) | 5.6 to 17.6 | N/A | N/A | -3.5 (10.9) | -4.5 to -2.6 | 10.5 (32.9) | 3.9 to 17 | N/A | N/A | -0.1 (11.6) | -1.5 to 1.3 | 15.2 (40.3) | 0.7 to 29.8 |
| LDL_Hattori_ | N/A | N/A | -16.8 (11.2) | -17.6 to -16 | -35.9 (31) | -41.2 to -30.5 | N/A | N/A | -18.2 (10.8) | -19.2 to -17.2 | -39.6 (30.7) | -45.8 to -33.5 | N/A | N/A | -14.4 (11.4) | -15.7 to -12.9 | -24.2 (29.6) | -34.9 to -13.5 |
| LDL_Anadaraja_ | N/A | N/A | -10.3 (12.8) | -11.2 to -9.4 | -31.8 (30.6) | -37.1 to -26.5 | N/A | N/A | -12.2 (12.8) | -13.4 to -11.1 | -35.6 (30.9) | -41.8 to -29.4 | N/A | N/A | -6.7 (12) | -8.2 to -5.3 | -20.1 (27) | -29.9 to -10.3 |
| LDL_Ahmadi_ | N/A | N/A | 113.3 (35) | 110.8 to 115.7 | 329.5 (146) | 304.2 to 354.7 | N/A | N/A | 114.8 (35.4) | 111.6 to 117.9 | 337.8 (155.8) | 306.7 to 368.8 | N/A | N/A | 110.6 (34.1) | 106.5 to 114.6 | 303.8 (108.7) | 264.6 to 342.9 |
| LDL_Puavilai_ | N/A | N/A | -0.6 (11.4) | -1.4 to 0.2 | -11.2 (28.6) | -16.2 to -6.3 | N/A | N/A | -1.9 (11) | -2.9 to -0.9 | -14.9 (26.4) | -20.2 to -9.7 | N/A | N/A | 1.8 (11.6) | 0.4 to 3.2 | 0.3 (32.5) | -11.4 to 12.0 |
| LDL_Vujovic_ | N/A | N/A | 3.2 (11.3) | 2.3 to 4 | -2.6 (27.2) | -7.3 to 2.1 | N/A | N/A | 1.9 (11) | 0.9 to 2.9 | -6.1 (24.3) | -10.9 to -1.2 | N/A | N/A | 5.4 (11.6) | 4 to 0.8 | 8.1 (32.8) | -3.7 to 19.9 |
| LDL_Chen and Zhang_ | N/A | N/A | 0.1 (10.8) | -0.7 to 0.9 | 10.3 (22.8) | 6.3 to 14.2 | N/A | N/A | -0.9 (10.3) | -1.8 to 0 | 8.2 (19) | 4.4 to 11.9 | N/A | N/A | 1.9 (11.4) | 0.6 to 3.3 | 16.8 (31.3) | 5.5 to 28.1 |
| LDL_de Cordova_ | N/A | N/A | 1.4 (13.3) | 0.5 to 2.4 | 42.7 (28.9) | 37.7 to 47.7 | N/A | N/A | 0.9 (12.7) | -0.2 to 2 | 43.0 (26.7) | 37.7 to 48.3 | N/A | N/A | 2.4 (14.5) | 0.6 to 4.1 | 41.9 (35.3) | 29.2 to 54.6 |
| LDL_Martin_ | N/A | N/A | 3.0 (10.8) | 2.2 to 3.8 | 12.3 (24.5) | 8.1 to 16.6 | N/A | N/A | 2 (10.4) | 1.1 to 2.9 | 9.6 (22) | 5.2 to 14 | N/A | N/A | 4.8 (11.4) | 3.4 to 6.2 | 20.9 (29.9) | 10.1 to 31.6 |
| LDL_Choi_ | N/A | N/A | 14.5 (11.2) | 13.7 to 15.3 | 15.2 (25.7) | 10.8 to 19.7 | N/A | N/A | 13.2 (10.9) | 12.3 to 14.2 | 12.1 (21.9) | 7.7 to 16.4 | N/A | N/A | 16.8 (11.4) | 15.4 to 18.2 | 25.1 (33.5) | 13 to 37.2 |

*N/A* Not available because of limited numbers of test results, *SD* standard deviation

The top three equations are presented in colors: red represents the highest, orange represents the second, and yellow represents the third highest intraclass correlation coefficient, or the equations with the lowest, second lowest, and third lowest mean systemic differences.

**Supplementary Table S6.** Limit of agreement and absolute percentage errors among the 12 equations in comparison with directly measured LDL according to subgroups by triglyceride (TG) concentration

|  | **Total subjects** | | | | | | | | | | | |
| --- | --- | --- | --- | --- | --- | --- | --- | --- | --- | --- | --- | --- |
|  | **TG group 1 (TG < 175 mg/dL)** | | | | | | | | | | | |
|  | **Population 1 (Test n=3566)** | | | | **Population 2 (Test n=1546)** | | | | **Population 3 (Test n=0)** | | | |
| Limits of agreement | Lower | 95%CI | Upper | 95%CI | Lower | 95%CI | Upper | 95%CI | Lower | 95%CI | Upper | 95%CI |
| LDL_Friedwald_ | -26.7 | -27 to -26.3 | -2.5 | -2.8 to -2.2 | -26.6 | -27 to -26.1 | -3.7 | -4.2 to -3.3 | N/A | N/A | N/A | N/A |
| LDL_DeLong_ | -22.0 | -22.4 to -21.7 | 1.3 | 1 to 1.7 | -22.3 | -22.8 to -21.8 | 0.4 | -0.1 to 0.9 | N/A | N/A | N/A | N/A |
| LDL_Rao_ | -25.3 | -25.6 to -25 | -1.7 | -2.1 to -1.4 | -25.3 | -25.8 to -24.9 | -2.8 | -3.3 to -2.3 | N/A | N/A | N/A | N/A |
| LDL_Hattori_ | -34.0 | -34.4 to -33.6 | -7.3 | -7.7 to -7 | -34.4 | -34.9 to -33.8 | -7.9 | -8.4 to -7.3 | N/A | N/A | N/A | N/A |
| LDL_Anadaraja_ | -27.5 | -28.3 to -26.7 | 28.2 | 27.4 to 29 | -26.3 | -27.5 to -25.2 | 27.5 | 26.3 to 28.6 | N/A | N/A | N/A | N/A |
| LDL_Ahmadi_ | -46.0 | -47.3 to -44.6 | 48.9 | 47.5 to 50.2 | -48.8 | -51.0 to -46.6 | 50.5 | 48.4 to 52.7 | N/A | N/A | N/A | N/A |
| LDL_Puavilai_ | -22.7 | -23 to -22.3 | 0.7 | 0.4 to 1.1 | -22.8 | -23.3 to -22.3 | -0.3 | -0.8 to 0.2 | N/A | N/A | N/A | N/A |
| LDL_Vujovic_ | -21.2 | -21.5 to -20.8 | 2.2 | 1.8 to 2.5 | -21.5 | -22 to -21 | 1.3 | 0.8 to 1.8 | N/A | N/A | N/A | N/A |
| LDL_Chen and Zhang_ | -30.1 | -30.5 to -29.7 | -1.5 | -1.9 to -1.1 | -31.5 | -32.1 to -30.8 | -1.0 | -1.7 to -0.3 | N/A | N/A | N/A | N/A |
| LDL_de Cordova_ | -45.4 | -46 to -44.7 | -0.6 | -1.2 to 0.1 | -47.7 | -48.8 to -46.6 | 1.5 | 0.4 to 2.6 | N/A | N/A | N/A | N/A |
| LDL_Martin_ | -26 | -26.3 to -25.6 | -1.3 | -1.6 to -0.9 | -26.8 | -27.4 to -26.3 | -1.5 | -2.1 to -1 | N/A | N/A | N/A | N/A |
| LDL_Choi_ | -10.2 | -10.5 to -9.9 | 11.1 | 10.8 to 11.4 | -10 | -10.4 to -9.5 | 9.9 | 9.4 to 10.3 | N/A | N/A | N/A | N/A |
| Absolute percentage error | Med | 95% CI | 95th P | 95% CI | Med | 95% CI | 95th P | 95% CI | Med | 95% CI | 95th P | 95% CI |
| LDL_Friedwald_ | 13.3 | 13.1 to 13.6 | 25.0 | 24.4 to 25.6 | 14.0 | 13.6 to 14.3 | 26.1 | 25.3 to 27.6 | N/A | N/A | N/A | N/A |
| LDL_DeLong_ | 9.5 | 9.3 to 9.7 | 19.3 | 18.8 to 19.8 | 10.0 | 9.7 to 10.2 | 20.5 | 19.6 to 21.2 | N/A | N/A | N/A | N/A |
| LDL_Rao_ | 12.5 | 12.2 to 12.8 | 23.5 | 23.1 to 23.9 | 12.9 | 12.7 to 13.3 | 24.8 | 23.9 to 25.7 | N/A | N/A | N/A | N/A |
| LDL_Hattori_ | 18.8 | 18.5 to 19 | 29.7 | 29.1 to 30.4 | 19.3 | 19 to 19.6 | 30.8 | 30 to 32 | N/A | N/A | N/A | N/A |
| LDL_Anadaraja_ | 8.7 | 8.3 to 8.9 | 28.6 | 27.4 to 30.5 | 8.4 | 8.1 to 8.8 | 30.3 | 28.1 to 35 | N/A | N/A | N/A | N/A |
| LDL_Ahmadi_ | 16.7 | 16 to 17.2 | 52.1 | 50 to 54.4 | 17.4 | 16.4 to 18.4 | 55.4 | 50 to 61.2 | N/A | N/A | N/A | N/A |
| LDL_Puavilai_ | 10.1 | 9.8 to 10.3 | 20.0 | 19.5 to 20.7 | 10.5 | 10.1 to 10.9 | 21.4 | 20.6 to 22.3 | N/A | N/A | N/A | N/A |
| LDL_Vujovic_ | 8.8 | 8.5 to 9 | 18.3 | 17.8 to 18.8 | 9.2 | 8.9 to 9.4 | 19.3 | 18.5 to 20.5 | N/A | N/A | N/A | N/A |
| LDL_Chen and Zhang_ | 14.1 | 14 to 14.3 | 22.4 | 22 to 22.7 | 14.7 | 14.5 to 15 | 23.0 | 22.4 to 23.8 | N/A | N/A | N/A | N/A |
| LDL_de Cordova_ | 20.3 | 20 to 20.5 | 29.2 | 28.8 to 29.5 | 20.7 | 20.3 to 21 | 30.1 | 29.7 to 30.6 | N/A | N/A | N/A | N/A |
| LDL_Martin_ | 12.4 | 12.1 to 12.6 | 22.8 | 22 to 23.4 | 12.9 | 12.5 to 13.2 | 24.1 | 23.4 to 24.7 | N/A | N/A | N/A | N/A |
| LDL_Choi_ | 3.1 | 3 to 3.2 | 10.5 | 10.2 to 11.3 | 3.1 | 2.9 to 3.3 | 10.4 | 9.8 to 11.1 | N/A | N/A | N/A | N/A |

(*Continue*)

**Supplementary Table S6**. (*Continue*)

|  | **Total subjects** | | | | | | | | | | | |
| --- | --- | --- | --- | --- | --- | --- | --- | --- | --- | --- | --- | --- |
|  | **TG group 2 (TG 175-400 mg/dL)** | | | | | | | | | | | |
|  | **Population 1 (Test n=1330)** | | | | **Population 2 (Test n=1546)** | | | | **Population 3 (Test n=758)** | | | |
| Limits of agreement | Lower | 95%CI | Upper | 95%CI | Lower | 95%CI | Upper | 95%CI | Lower | 95% CI | Upper | 95% CI |
| LDL_Friedwald_ | -40.1 | -40.9 to -39.3 | -7.5 | -8.3 to -6.7 | -38 | -39.2 to -36.8 | -6.3 | -7.4 to -5.1 | -32.3 | -33.7 to -30.8 | 13.5 | 12.1 to 14.9 |
| LDL_DeLong_ | -28.4 | -29 to -27.7 | 0.2 | -0.5 to 0.9 | -27.1 | -28.1 to -26 | 2.2 | 1.1 to 0.3 | -21.1 | -22.5 to -19.7 | 23.3 | 21.9 to 24.6 |
| LDL_Rao_ | -31.4 | -32.1 to -30.8 | -3.9 | -4.5 to -3.2 | -30.6 | -31.7 to -29.5 | -1.6 | -2.6 to -0.5 | -24.4 | -25.8 to -23 | 19.8 | 18.4 to 21.1 |
| LDL_Hattori_ | -46.7 | -47.5 to -45.9 | -13.3 | -14.1 to -12.5 | -44.6 | -45.8 to -43.4 | -12.1 | -13.3 to -10.8 | -38.7 | -40.1 to -37.3 | 5.0 | 3.7 to 6.4 |
| LDL_Anadaraja_ | -43.7 | -44.8 to -42.6 | 4.8 | 3.6 to 0.9 | -39.9 | -41.6 to -38.3 | 5.5 | 3.8 to 0.1 | -35.4 | -37 to -33.8 | 14.8 | 13.3 to 16.4 |
| LDL_Ahmadi_ | 14.6 | 11.1 to 8.1 | 162.9 | 159.4 to 166.3 | 17.4 | 12.1 to 2.7 | 162.2 | 156.8 to 167.5 | 44.7 | 40.4 to 48.9 | 181.9 | 177.6 to 186.1 |
| LDL_Puavilai_ | -30.1 | -30.8 to -29.4 | -1.2 | -1.8 to -0.5 | -28.8 | -29.9 to -27.7 | 0.8 | -0.3 to 1.9 | -22.9 | -24.3 to -21.4 | 21.7 | 20.3 to 23.1 |
| LDL_Vujovic_ | -26.1 | -26.8 to -25.5 | 1.8 | 1.2 to 0.5 | -25.1 | -26.1 to -24 | 4.1 | 3.0 to 0.2 | -19 | -20.4 to -17.6 | 25.3 | 23.9 to 26.7 |
| LDL_Chen and Zhang_ | -29.2 | -29.9 to -28.5 | 1.7 | 0.9 to 0.4 | -28.9 | -30.2 to -27.7 | 4.5 | 3.2 to 0.7 | -21 | -22.3 to -19.6 | 21.2 | 19.9 to 22.5 |
| LDL_de Cordova_ | -36.1 | -37.2 to -34.9 | 14.5 | 13.3 to 15.6 | -36.8 | -38.8 to -34.8 | 18.1 | 16 to 20.1 | -24.7 | -26.3 to -23 | 27.6 | 26 to 29.2 |
| LDL_Martin_ | -26.2 | -26.9 to -25.5 | 4.2 | 3.5 to 0.9 | -25.8 | -27 to -24.6 | 7.4 | 6.2 to 0.6 | -18.3 | -19.6 to -16.9 | 24.3 | 22.9 to 25.6 |
| LDL_Choi_ | -13.8 | -14.4 to -13.2 | 12.3 | 11.7 to 12.9 | -12.7 | -13.7 to -11.7 | 14.6 | 13.6 to 15.6 | -7.4 | -8.8 to -6.1 | 36.5 | 35.1 to 37.8 |
| Absolute percentage error | Med | 95% CI | 95th P | 95% CI | Med | 95% CI | 95th P | 95% CI | Med | 95% CI | 95th P | 95% CI |
| LDL_Friedwald_ | 20.6 | 20.1 to 21.2 | 41.9 | 39.8 to 44.7 | 18.9 | 18.1 to 19.7 | 42.4 | 37.7 to 48.4 | 8.9 | 8.2 to 9.7 | 28.2 | 26.2 to 32.8 |
| LDL_DeLong_ | 12.3 | 11.8 to 12.7 | 26.7 | 25.6 to 28.6 | 11.1 | 10.5 to 11.5 | 25.6 | 23.9 to 28.9 | 5.4 | 5.0 to 5.9 | 20.6 | 18.7 to 25.3 |
| LDL_Rao_ | 15.5 | 15.1 to 15.9 | 30.7 | 29.3 to 32.5 | 14.2 | 13.6 to 14.8 | 31.3 | 29.5 to 34.3 | 5.9 | 5.4 to 6.4 | 20.8 | 19.4 to 24.2 |
| LDL_Hattori_ | 25.8 | 25.3 to 26.3 | 46.5 | 43.9 to 48.9 | 24.2 | 23.5 to 24.8 | 46.5 | 42.1 to 53.9 | 14.2 | 13.5 to 15 | 33.3 | 30.6 to 36.8 |
| LDL_Anadaraja_ | 17.0 | 16.5 to 17.5 | 37.7 | 36.1 to 39.3 | 15.3 | 14.5 to 16.2 | 34.3 | 31.7 to 36.3 | 9.5 | 9 to 10.3 | 28.4 | 27.2 to 31.8 |
| LDL_Ahmadi_ | 72.9 | 69.6 to 77 | 188.4 | 180.9 to 203.5 | 73.6 | 68.8 to 77.9 | 206.0 | 189.8 to 247.5 | 89.6 | 86 to 93 | 208.2 | 198.1 to 223.5 |
| LDL_Puavilai_ | 13.7 | 13.1 to 14.1 | 29.2 | 27.7 to 31.4 | 12.3 | 11.8 to 12.8 | 28.4 | 25.9 to 31.3 | 5.6 | 5.2 to 6.0 | 21.4 | 19.1 to 25.3 |
| LDL_Vujovic_ | 10.6 | 10.2 to 11.2 | 24.1 | 22.9 to 25.3 | 9.5 | 9 to 10 | 22.5 | 21.3 to 25.5 | 5.3 | 4.9 to 5.9 | 21.6 | 18.3 to 26.1 |
| LDL_Chen and Zhang_ | 12.1 | 11.7 to 12.4 | 20.0 | 19.5 to 20.7 | 10.6 | 9.9 to 11.3 | 19.3 | 18.5 to 20.2 | 5.1 | 4.7 to 5.5 | 19.1 | 17.4 to 24.1 |
| LDL_de Cordova_ | 10.3 | 9.9 to 10.8 | 20.4 | 20 to 21.3 | 10.2 | 9.2 to 11 | 22.0 | 19.9 to 24.6 | 6.6 | 6.0 to 7.1 | 29.0 | 25.3 to 37.1 |
| LDL_Martin_ | 9.9 | 9.5 to 10.2 | 18.0 | 17.3 to 18.6 | 8.5 | 7.9 to 9 | 17.1 | 16.1 to 18.5 | 5.1 | 4.6 to 5.5 | 21.7 | 18.8 to 26.9 |
| LDL_Choi_ | 3.5 | 3.3 to 3.7 | 12.5 | 11.8 to 14.1 | 3.9 | 3.7 to 4.3 | 14.4 | 12.1 to 17.3 | 11.6 | 10.7 to 12.2 | 30.7 | 27.6 to 37.8 |

(*Continue*)

**Supplementary Table S6**. (*Continue*)

|  | **Total subjects** | | | | | | | | | | | | | |
| --- | --- | --- | --- | --- | --- | --- | --- | --- | --- | --- | --- | --- | --- | --- |
|  | **TG group 3 (TG > 400 mg/dL)** | | | | | | | | | | | | | |
|  | **Population 1 (Test n=1330)** | | | | **Population 2 (Test n=75)** | | | | | **Population 3 (Test n=131)** | | | | |
| Limits of agreement | Lower | 95% CI | Upper | 95% CI | | Lower | 95% CI | Upper | 95% CI | | Lower | 95% CI | Upper | 95% CI |
| LDL_Friedwald_ | -93.3 | -97.6 to -89.1 | -6.6 | -10.8 to -2.3 | | -138.3 | -156.1 to -120.0 | 38.8 | 21.0 to 56.6 | | -95.6 | -105.3 to -85.8 | 33.6 | 23.8 to 43.3 |
| LDL_DeLong_ | -60.9 | -64.4 to -57.4 | 9.2 | 5.7 to 12.6 | | -75.8 | -86.4 to -65.3 | 28.9 | 18.4 to 39.5 | | -62.0 | -70.2 to -53.7 | 47.4 | 39.1 to 55.6 |
| LDL_Rao_ | -251.4 | -276.3 to -226.3 | 254.4 | 229.4 to 279.3 | | -393 | -477.7 to -308.3 | 447.8 | 363.1 to 532.5 | | -56.5 | -66.8 to -46.2 | 79.8 | 69.5 to 90.0 |
| LDL_Hattori_ | -96.3 | -100.4 to -92.2 | -13.5 | -17.6 to -9.4 | | -138.5 | -155.5 to -121 | 30.1 | 13.1 to 47 | | -96.7 | -105.8 to -87.5 | 24.9 | 15.7 to 34.1 |
| LDL_Anadaraja_ | -92.3 | -96.5 to -88.1 | -7.5 | -11.7 to -3.3 | | -128.7 | -144.6 to -112 | 29.7 | 13.7 to 45.6 | | -91.9 | -100.9 to -82.8 | 28.2 | 19.1 to 37.3 |
| LDL_Ahmadi_ | -141.7 | -187.1 to -96.3 | 776.7 | 731.3 to 822.1 | | -317.1 | -452.8 to -181 | 1030.1 | 894.3 to 1165 | | 43.3 | 0.0 to 86.5 | 615.7 | 572.4 to 658.9 |
| LDL_Puavilai_ | -64.9 | -68.4 to -61.5 | 5.3 | 1.8 to 8.8 | | -85.8 | -97.5 to -74.1 | 30.2 | 18.5 to 41.9 | | -67.3 | -75.8 to -58.9 | 44.9 | 36.4 to 53.4 |
| LDL_Vujovic_ | -56.8 | -60.3 to -53.3 | 14.7 | 11.2 to 18.2 | | -64.5 | -73.8 to -55.2 | 28 | 18.7 to 37.3 | | -55.9 | -63.9 to -47.8 | 50.7 | 42.6 to 58.7 |
| LDL_Chen and Zhang_ | -55.4 | -60.1 to -50.7 | 39.4 | 34.7 to 44.1 | | -40 | -47.5 to -32.5 | 34.8 | 27.2 to 42.3 | | -34.4 | -41.2 to -27.7 | 55.0 | 48.2 to 61.7 |
| LDL_de Cordova_ | -70.6 | -80.0 to -61.1 | 120.8 | 111.4 to 130.2 | | -75.8 | -98.2 to -53.3 | 146.8 | 124.4 to 169.1 | | -13.8 | -22.4 to -5.3 | 99.3 | 90.7 to 107.8 |
| LDL_Martin_ | -43.2 | -46.8 to -39.6 | 30.2 | 26.6 to 33.8 | | -50.2 | -59.7 to -40.7 | 44.3 | 34.7 to 53.8 | | -35.7 | -42.9 to -28.4 | 60.4 | 53.1 to 67.6 |
| LDL_Choi_ | -43.8 | -47.9 to -39.8 | 38.2 | 34.1 to 42.2 | | -34.8 | -42 to -27.6 | 36.7 | 29.5 to 43.9 | | -35.2 | -42.8 to -27.5 | 65.7 | 58.0 to 73.3 |
| Absolute percentage error | Med | 95% CI | 95th P | 95% CI | | Med | 95% CI | 95th P | 95% CI | | Med | 95% CI | 95th P | 95% CI |
| LDL_Friedwald_ | 40.6 | 38.6 to 43.0 | 131.9 | 118 to 158.6 | | 38.9 | 31.1 to 48.6 | 356.9 | 89 to 698.1 | | 33.3 | 27.0 to 40.2 | 131.2 | 106.1 to 201.1 |
| LDL_DeLong_ | 22.4 | 20.9 to 23.8 | 73.7 | 66.9 to 83 | | 17.0 | 13.8 to 25.7 | 132.1 | 53.7 to 331.2 | | 16.1 | 12.9 to 21.6 | 72.0 | 62.0 to 121.3 |
| LDL_Rao_ | 17.9 | 16.5 to 19.6 | 80.5 | 58.2 to 256 | | 13.4 | 11.3 to 16.6 | 117.1 | 41.5 to 4028 | | 14.1 | 11.5 to 18.5 | 105.5 | 60.7 to 186.5 |
| LDL_Hattori_ | 45.3 | 43.0 to 47.2 | 132.6 | 119.6 to 159.5 | | 43.5 | 36.5 to 52.3 | 355.7 | 92.2 to 683.2 | | 38.7 | 31.7 to 44.9 | 131.2 | 108.5 to 200.6 |
| LDL_Anadaraja_ | 40.5 | 38.4 to 43.3 | 133.6 | 115.7 to 153.7 | | 38.7 | 34.0 to 44.6 | 373.5 | 93.9 to 592.8 | | 31.1 | 28.2 to 38.7 | 118.3 | 99.1 to 209.3 |
| LDL_Ahmadi_ | 233.7 | 214.9 to 256.8 | 992.6 | 803.3 to 1450.0 | | 272.6 | 219.4 to 319.5 | 2266.7 | 870.2 to 6458.5 | | 339.1 | 317.8 to 403.2 | 1096 | 850.2 to 1360.0 |
| LDL_Puavilai_ | 25.1 | 24.0 to 27.2 | 83.3 | 75.3 to 94.3 | | 23.4 | 16.9 to 28.7 | 152.7 | 59.5 to 376.7 | | 17.4 | 14.6 to 22.7 | 84.8 | 70.2 to 134.1 |
| LDL_Vujovic_ | 19.0 | 17.7 to 20.3 | 63.2 | 54.7 to 72.7 | | 17.4 | 12.3 to 22.5 | 114.6 | 46.6 to 282.3 | | 14 | 11.2 to 19.4 | 63.1 | 55.5 to 106.8 |
| LDL_Chen and Zhang_ | 12.7 | 11.6 to 13.9 | 36.8 | 32.1 to 50.3 | | 10.8 | 8.5 to 13.8 | 92.8 | 36.2 to 245.8 | | 14.1 | 11.0 to 19.1 | 68.4 | 56.3 to 107.9 |
| LDL_de Cordova_ | 13.6 | 11.2 to 18.0 | 149.5 | 102.4 to 205.4 | | 27 | 16.2 to 37.7 | 289.7 | 97.6 to 1011 | | 44.3 | 36.6 to 51.6 | 179.7 | 124.5 to 234.9 |
| LDL_Martin_ | 9.2 | 8.2 to 10.4 | 40.8 | 31.6 to 53.3 | | 11.6 | 8.8 to 15.3 | 100.0 | 46 to 262.3 | | 16.7 | 12.8 to 22.7 | 78.9 | 59.7 to 122.0 |
| LDL_Choi_ | 7.8 | 7.0 to 9.0 | 37.1 | 29.4 to 54.6 | | 8.6 | 5.7 to 11.6 | 97.8 | 43.8 to 154.6 | | 19.5 | 16.0 to 22.4 | 78.3 | 54.0 to 117.3 |

(*Continue*)

**Supplementary Table S6**. (*Continue*)

|  | **Men** | | | | | | | | | | | |
| --- | --- | --- | --- | --- | --- | --- | --- | --- | --- | --- | --- | --- |
|  | **TG group 1 (TG < 175 mg/dL)** | | | | | | | | | | | |
|  | **Population 1 (Test n=1517)** | | | | **Population 2 (Test n=669)** | | | | **Population 3 (Test n=0)** | | | |
| Limits of agreement | Lower | 95%CI | Upper | 95%CI | Lower | 95% CI | Upper | 95% CI | Lower | 95%CI | Upper | 95%CI |
| LDL_Friedwald_ | -27.1 | -27.6 to -26.5 | -1.8 | -2.4 to -1.3 | -25.8 | -26.6 to -25.1 | -3.5 | -4.3 to -2.8 | N/A | N/A | N/A | N/A |
| LDL_DeLong_ | -22.2 | -22.7 to -21.6 | 2.1 | 1.6 to 2.7 | -21.2 | -21.9 to -20.4 | 0.7 | 0 to 1.5 | N/A | N/A | N/A | N/A |
| LDL_Rao_ | -25.6 | -26.1 to -25 | -1.0 | -1.5 to -0.5 | -24.4 | -25.2 to -23.7 | -2.6 | -3.3 to -1.8 | N/A | N/A | N/A | N/A |
| LDL_Hattori_ | -34.2 | -34.8 to -33.6 | -6.5 | -7.1 to -5.9 | -33.4 | -34.2 to -32.5 | -7.7 | -8.5 to -6.8 | N/A | N/A | N/A | N/A |
| LDL_Anadaraja_ | -28.9 | -30 to -27.8 | 21.8 | 20.7 to 23 | -27.6 | -29.2 to -25.9 | 21.4 | 19.7 to 23 | N/A | N/A | N/A | N/A |
| LDL_Ahmadi_ | -40.5 | -42.6 to -38.5 | 52.1 | 50.1 to 54.2 | -41.9 | -45.1 to -38.7 | 54.3 | 51.1 to 57.5 | N/A | N/A | N/A | N/A |
| LDL_Puavilai_ | -22.8 | -23.4 to -22.3 | 1.5 | 1 to 2 | -21.8 | -22.5 to -21.0 | 0.0 | -0.7 to 0.8 | N/A | N/A | N/A | N/A |
| LDL_Vujovic_ | -21.2 | -21.8 to -20.7 | 3.0 | 2.4 to 3.5 | -20.3 | -21.1 to -19.6 | 1.7 | 1.0 to 0.4 | N/A | N/A | N/A | N/A |
| LDL_Chen and Zhang_ | -29.5 | -30.2 to -28.9 | -0.4 | -1 to 0.2 | -29.6 | -30.6 to -28.6 | -0.4 | -1.4 to 0.5 | N/A | N/A | N/A | N/A |
| LDL_de Cordova_ | -43.6 | -44.6 to -42.6 | 1.1 | 0.1 to 2.1 | -44.9 | -46.5 to -43.3 | 2.7 | 1.1 to 0.3 | N/A | N/A | N/A | N/A |
| LDL_Martin_ | -25.8 | -26.3 to -25.2 | -0.3 | -0.8 to 0.3 | -25.3 | -26.1 to -24.4 | -1.1 | -1.9 to -0.3 | N/A | N/A | N/A | N/A |
| LDL_Choi_ | -10.8 | -11.3 to -10.3 | 11.4 | 10.9 to 11.9 | -9.5 | -10.1 to -8.9 | 9.9 | 9.3 to 10.6 | N/A | N/A | N/A | N/A |
| Absolute percentage error | Med | 95% CI | 95th P | 95% CI | Med | 95% CI | 95th P | 95% CI | Med | 95% CI | 95th P | 95% CI |
| LDL_Friedwald_ | 13.6 | 13.2 to 14 | 26.0 | 25 to 27.3 | 14.0 | 13.5 to 14.5 | 27.0 | 25.5 to 28.7 | N/A | N/A | N/A | N/A |
| LDL_DeLong_ | 9.6 | 9.2 to 9.9 | 19.4 | 18.8 to 20.1 | 9.6 | 9.2 to 10.0 | 20.6 | 19.6 to 22.0 | N/A | N/A | N/A | N/A |
| LDL_Rao_ | 12.7 | 12.3 to 13 | 24.0 | 23.3 to 25.4 | 12.9 | 12.4 to 13.2 | 25.3 | 24.1 to 27.0 | N/A | N/A | N/A | N/A |
| LDL_Hattori_ | 19.0 | 18.6 to 19.3 | 30.9 | 29.8 to 32.0 | 19.3 | 18.8 to 19.7 | 31.6 | 30.3 to 33.3 | N/A | N/A | N/A | N/A |
| LDL_Anadaraja_ | 8.6 | 8.2 to 9.0 | 26.4 | 25 to 29.0 | 8.6 | 8.2 to 9.4 | 27.7 | 24.3 to 31.9 | N/A | N/A | N/A | N/A |
| LDL_Ahmadi_ | 16.7 | 15.8 to 17.5 | 56.2 | 52.4 to 60.3 | 17.0 | 15.8 to 18.7 | 62.8 | 56.1 to 69.9 | N/A | N/A | N/A | N/A |
| LDL_Puavilai_ | 10.2 | 9.8 to 10.5 | 20.3 | 19.7 to 21.4 | 10.1 | 9.7 to 10.6 | 21.8 | 20.6 to 23.0 | N/A | N/A | N/A | N/A |
| LDL_Vujovic_ | 8.7 | 8.3 to 9.0 | 18.4 | 17.7 to 19.0 | 8.7 | 8.3 to 9.2 | 19.8 | 18.1 to 21.4 | N/A | N/A | N/A | N/A |
| LDL_Chen and Zhang_ | 13.9 | 13.6 to 14.2 | 22.2 | 21.8 to 22.9 | 14.1 | 13.6 to 14.6 | 22.6 | 21.4 to 23.7 | N/A | N/A | N/A | N/A |
| LDL_de Cordova_ | 19.6 | 19.2 to 20 | 28.4 | 27.7 to 29.1 | 19.4 | 18.9 to 19.9 | 28.7 | 27.6 to 29.9 | N/A | N/A | N/A | N/A |
| LDL_Martin_ | 12.3 | 11.9 to 12.6 | 22.8 | 21.9 to 24.1 | 12.3 | 12 to 12.7 | 24.0 | 23.2 to 25.2 | N/A | N/A | N/A | N/A |
| LDL_Choi_ | 3.1 | 3.0 to 3.3 | 11.4 | 10.5 to 12.9 | 3.0 | 2.7 to 3.3 | 10.5 | 9.9 to 11.8 | N/A | N/A | N/A | N/A |

(*Continue*)

**Supplementary Table S6**. (*Continue*)

|  | **Men** | | | | | | | | | | | |
| --- | --- | --- | --- | --- | --- | --- | --- | --- | --- | --- | --- | --- |
|  | **TG group 2 (TG 175-400 mg/dL)** | | | | | | | | | | | |
|  | **Population 1 (Test n=1517)** | | | | **Population 2 (Test n=669)** | | | | **Population 3 (Test n=489)** | | | |
| Limits of agreement | Lower | 95%CI | Upper | 95%CI | Lower | 95% CI | Upper | 95% CI | Lower | 95% CI | Upper | 95% CI |
| LDL_Friedwald_ | -41.2 | -42.2 to -40.2 | -7.7 | -8.7 to -6.7 | -39.0 | -40.5 to -37.4 | -6.5 | -8.0 to -5.0 | -33.1 | -34.9 to -31.4 | 11.5 | 9.8 to 13.2 |
| LDL_DeLong_ | -29.1 | -30 to -28.2 | 0.1 | -0.8 to 1.0 | -27.5 | -28.9 to -26.1 | 1.9 | 0.5 to 3.3 | -21.7 | -23.4 to -20 | 21.3 | 19.7 to 23 |
| LDL_Rao_ | -32 | -32.9 to -31.2 | -4.0 | -4.8 to -3.2 | -30.9 | -32.2 to -29.5 | -1.8 | -3.2 to -0.4 | -24.9 | -26.6 to -23.2 | 17.9 | 16.2 to 19.5 |
| LDL_Hattori_ | -47.6 | -48.7 to -46.6 | -13.2 | -14.3 to -12.2 | -45.4 | -47 to -43.8 | -12.3 | -13.8 to -10.7 | -39.4 | -41 to -37.7 | 3.0 | 1.3 to 4.6 |
| LDL_Anadaraja_ | -44.5 | -45.9 to -43.1 | 2.9 | 1.5 to 4.3 | -40.7 | -42.7 to -38.6 | 3 | 1.0 to 5.1 | -37.4 | -39.3 to -35.4 | 12.9 | 10.9 to 14.8 |
| LDL_Ahmadi_ | 17.2 | 12.7 to 21.7 | 167.3 | 162.8 to 171.8 | 18.5 | 11.5 to 25.6 | 168.6 | 161.6 to 175.6 | 45.3 | 39.9 to 50.7 | 184.3 | 178.9 to 189.6 |
| LDL_Puavilai_ | -30.9 | -31.8 to -30.0 | -1.3 | -2.2 to -0.4 | -29.3 | -30.7 to -27.9 | 0.5 | -0.9 to 1.9 | -23.5 | -25.2 to -21.8 | 19.7 | 18 to 21.4 |
| LDL_Vujovic_ | -26.8 | -27.6 to -25.9 | 1.8 | 0.9 to 2.6 | -25.4 | -26.8 to -24 | 3.8 | 2.4 to 5.2 | -19.6 | -21.2 to -17.9 | 23.4 | 21.7 to 25 |
| LDL_Chen and Zhang_ | -29.2 | -30.2 to -28.3 | 1.9 | 1 to 2.8 | -28.8 | -30.4 to -27.2 | 4.5 | 2.9 to 6.0 | -21 | -22.6 to -19.4 | 19.3 | 17.7 to 20.9 |
| LDL_de Cordova_ | -34.9 | -36.4 to -33.4 | 15.2 | 13.7 to 16.7 | -36.6 | -39.2 to -33.9 | 19.4 | 16.7 to 22.0 | -23.9 | -25.9 to -22 | 25.8 | 23.8 to 7.7 |
| LDL_Martin_ | -26.3 | -27.2 to -25.4 | 4.5 | 3.6 to 5.4 | -25.6 | -27.2 to -24.0 | 7.5 | 6 to 9.1 | -18.4 | -20 to -16.8 | 22.4 | 20.8 to 24 |
| LDL_Choi_ | -14.4 | -15.2 to -13.7 | 12.1 | 11.3 to 12.9 | -12.9 | -14.2 to -11.6 | 14.1 | 12.8 to 15.3 | -8.1 | -9.8 to -6.4 | 34.6 | 32.9 to 36.3 |
| Absolute percentage error | Med | 95% CI | 95th P | 95% CI | Med | 95% CI | 95th P | 95% CI | Med | 95% CI | 95th P | 95% CI |
| LDL_Friedwald_ | 21.9 | 21 to 22.6 | 44.6 | 41.9 to 48 | 19.7 | 18.5 to 20.8 | 47.0 | 41.1 to 54.7 | 9.7 | 8.9 to 10.5 | 29.5 | 26.5 to 35.2 |
| LDL_DeLong_ | 12.9 | 12.4 to 13.6 | 29.0 | 27.3 to 31.1 | 11.3 | 10.7 to 12.3 | 28.3 | 25.0 to 33.4 | 5.3 | 4.6 to 5.9 | 20.0 | 17.4 to 25.5 |
| LDL_Rao_ | 16.2 | 15.6 to 16.9 | 32.5 | 30.1 to 36.6 | 14.4 | 13.7 to 15.3 | 33.3 | 30.4 to 39.7 | 6.0 | 5.4 to 6.6 | 20.8 | 19.2 to 24.7 |
| LDL_Hattori_ | 26.9 | 26.1 to 27.6 | 48.9 | 46.4 to 52.1 | 25.0 | 23.8 to 25.9 | 51.0 | 45.4 to 58.3 | 15.1 | 14.3 to 15.9 | 33.9 | 31.0 to 39.5 |
| LDL_Anadaraja_ | 18.3 | 17.4 to 19.3 | 38.3 | 36.7 to 41.3 | 16.8 | 15.4 to 17.8 | 35.9 | 33.5 to 39.7 | 10.6 | 9.7 to 11.8 | 30.0 | 27.3 to 35.3 |
| LDL_Ahmadi_ | 80.8 | 76.3 to 84.8 | 191.2 | 179.6 to 210.3 | 78.6 | 72.1 to 86.1 | 242.7 | 202.1 to 285.8 | 90.9 | 86.1 to 96.2 | 202.0 | 191.5 to 224 |
| LDL_Puavilai_ | 14.3 | 13.8 to 14.9 | 31.7 | 29.3 to 33.3 | 12.7 | 11.9 to 13.6 | 30.9 | 26.8 to 36.2 | 5.6 | 5.1 to 6.1 | 21.3 | 18.9 to 25.0 |
| LDL_Vujovic_ | 11.4 | 10.8 to 11.9 | 25.5 | 24.1 to 27.3 | 9.8 | 9.2 to 10.7 | 24.5 | 22.1 to 28.4 | 4.9 | 4.4 to 5.6 | 19.7 | 17.3 to 26.4 |
| LDL_Chen and Zhang_ | 12.3 | 11.7 to 12.7 | 20.4 | 19.6 to 21.0 | 10.8 | 10.0 to 11.4 | 19.1 | 18.5 to 21.9 | 5.0 | 4.6 to 5.6 | 18.2 | 15.8 to 24.4 |
| LDL_de Cordova_ | 9.8 | 9.1 to 10.3 | 20.5 | 20 to 22.3 | 10.3 | 9.1 to 11.5 | 22.4 | 19.9 to 32.7 | 6.3 | 5.5 to 6.8 | 28.2 | 23.6 to 40.5 |
| LDL_Martin_ | 10.0 | 9.6 to 10.5 | 18.1 | 17.4 to 18.8 | 8.6 | 7.9 to 9.2 | 17.1 | 15.9 to 18.7 | 4.8 | 4.3 to 5.3 | 21.3 | 16.7 to 26.9 |
| LDL_Choi_ | 3.8 | 3.5 to 4.1 | 13.5 | 12.1 to 14.8 | 3.8 | 3.5 to 4.2 | 15.4 | 12.1 to 18.3 | 10.0 | 9.3 to 11.5 | 28.6 | 25.2 to 38.5 |

(*Continue*)

**Supplementary Table S6**. (*Continue*)

|  | **Men** | | | | | | | | | | | |
| --- | --- | --- | --- | --- | --- | --- | --- | --- | --- | --- | --- | --- |
|  | **TG group 3 (TG > 400 mg/dL)** | | | | | | | | | | | |
|  | **Population 1 (Test n=234)** | | | | **Population 2 (Test n=57)** | | | | **Population 3 (Test n=99)** | | | |
| Limits of agreement | Lower | 95% CI | Upper | 95% CI | Lower | 95% CI | Upper | 95% CI | Lower | 95% CI | Upper | 95% CI |
| LDL_Friedwald_ | -93.3 | -97.6 to -89.1 | -6.6 | -10.8 to -2.3 | -122.3 | -139.6 to -104.8 | 27.4 | 10 to 44.8 | -98.6 | -109.6 to -87.5 | 28.1 | 17 to 39.1 |
| LDL_DeLong_ | -60.9 | -64.4 to -57.4 | 9.2 | 5.7 to 12.6 | -73.6 | -85.5 to 61.8 | 28.2 | 16.4 to 40.1 | -60.6 | -69.3 to -51.9 | 38.8 | 30.2 to 47.5 |
| LDL_Rao_ | -251.4 | -276.3 to -226.3 | 254.4 | 229.4 to 279.3 | -163.7 | -203.4 to -123.9 | 177.7 | 137.9 to 217.3 | -54.0 | -65.3 to -42.8 | 75.0 | 63.7 to 86.2 |
| LDL_Hattori_ | -96.3 | -100.4 to -92.2 | -13.5 | -17.6 to -9.4 | -122.6 | -139.1 to -106 | 19.4 | 2.9 to 35.9 | -99.7 | -110.2 to -89.2 | 20.4 | 9.9 to 30.9 |
| LDL_Anadaraja_ | -92.3 | -96.5 to -88.1 | -7.5 | -11.7 to -3.3 | -118.3 | -134.7 to -101.9 | 22.8 | 6.4 to 39.2 | -96.2 | -106.7 to -85.5 | 24.9 | 14.4 to 35.5 |
| LDL_Ahmadi_ | -141.7 | -187.1 to -96.3 | 776.7 | 731.3 to 822.1 | -117.0 | -221.4 to -12.6 | 780.5 | 676 to 884.8 | 32.5 | -20.7 to 85.8 | 643.1 | 589.8 to 696.3 |
| LDL_Puavilai_ | -64.9 | -68.4 to -61.5 | 5.3 | 1.8 to 0.8 | -81.5 | -94.3 to -68.7 | 27.9 | 15.2 to 40.7 | -66.7 | -75.7 to -57.7 | 36.8 | 27.8 to 45.9 |
| LDL_Vujovic_ | -56.8 | -60.3 to -53.3 | 14.7 | 11.2 to 18.2 | -64.6 | -75.4 to -53.7 | 28.8 | 18 to 39.7 | -53.7 | -62 to -45.4 | 41.6 | 33.3 to 49.9 |
| LDL_Chen and Zhang_ | -55.4 | -60.1 to -50.7 | 39.4 | 34.7 to 44.1 | -37.5 | -45.5 to -29.5 | 31.2 | 23.2 to 39.2 | -29.1 | -35.5 to -22.6 | 45.4 | 38.9 to 51.9 |
| LDL_de Cordova_ | -70.6 | -80 to -61.1 | 120.8 | 111.4 to 130.2 | -41.8 | -59.2 to -24.4 | 107.2 | 89.9 to 124.5 | -9.3 | -18.4 to -0.1 | 95.2 | 86.1 to 104.3 |
| LDL_Martin_ | -43.2 | -46.8 to -39.6 | 30.2 | 26.6 to 33.8 | -49.3 | -60.4 to -38.1 | 46.3 | 35.2 to 57.4 | -33.5 | -41 to -26 | 52.6 | 45.1 to 60.2 |
| LDL_Choi_ | -43.8 | -47.9 to -39.8 | 38.2 | 34.1 to 42.2 | -37.5 | -46.2 to -28.6 | 38.1 | 29.3 to 46.9 | -30.9 | -38.4 to -23.4 | 55.0 | 47.5 to 62.5 |
| Absolute percentage error | Med | 95% CI | 95th P | 95% CI | Med | 95% CI | 95th P | 95% CI | Med | 95% CI | 95th P | 95% CI |
| LDL_Friedwald_ | 40.6 | 38.6 to 43 | 131.9 | 118 to 158.6 | 40.3 | 34.1 to 49.7 | 337.4 | N/A | 36.5 | 31.0 to 48.0 | 149.6 | 121.9 to 204.9 |
| LDL_DeLong_ | 22.4 | 20.9 to 23.8 | 73.7 | 66.9 to 83 | 22.2 | 14.2 to 26.3 | 127.1 | N/A | 16.9 | 13.3 to 24.0 | 79.4 | 65.1 to 121.1 |
| LDL_Rao_ | 17.9 | 16.5 to 19.6 | 80.5 | 58.2 to 256 | 14.5 | 11.9 to 20.4 | 114.6 | N/A | 14.1 | 11.3 to 19.1 | 125.7 | 62.3 to 186.5 |
| LDL_Hattori_ | 45.3 | 43 to 47.2 | 132.6 | 119.6 to 159.5 | 45.0 | 39 to 53.6 | 335.8 | N/A | 42.2 | 35.4 to 51.9 | 151.5 | 122.1 to 203.2 |
| LDL_Anadaraja_ | 40.5 | 38.4 to 43.3 | 133.6 | 115.7 to 153.7 | 39.4 | 34.1 to 47.1 | 349.5 | N/A | 36.4 | 30.1 to 47.8 | 131.6 | 101.5 to 216.6 |
| LDL_Ahmadi_ | 233.7 | 214.9 to 256.8 | 992.6 | 803.3 to 1450 | 309.5 | 221.1 to 375.2 | 2159.0 | N/A | 373.0 | 326.2 to 426.5 | 1110.3 | 866 to 1534 |
| LDL_Puavilai_ | 25.1 | 24 to 27.2 | 83.3 | 75.3 to 94.3 | 25.9 | 17.3 to 29.8 | 151.6 | N/A | 19.7 | 15.2 to 26 | 91.9 | 72.3 to 135.0 |
| LDL_Vujovic_ | 19.0 | 17.7 to 20.3 | 63.2 | 54.7 to 72.7 | 20.0 | 12.9 to 22.7 | 108.1 | N/A | 17.4 | 10.9 to 21.4 | 64.0 | 57.2 to 106.7 |
| LDL_Chen and Zhang_ | 12.7 | 11.6 to 13.9 | 36.8 | 32.1 to 50.3 | 11.2 | 8.4 to 14.7 | 90.7 | N/A | 13.7 | 10.6 to 18.8 | 69.5 | 57.5 to 107.8 |
| LDL_de Cordova_ | 13.6 | 11.2 to 18 | 149.5 | 102.4 to 205.4 | 28.3 | 14.2 to 51.4 | 287.1 | N/A | 45.2 | 39.8 to 55.4 | 177.6 | 120.6 to 243.5 |
| LDL_Martin_ | 9.2 | 8.2 to 10.4 | 40.8 | 31.6 to 53.3 | 12.2 | 10.1 to 16.5 | 100.0 | N/A | 18.2 | 12.9 to 23.6 | 79.7 | 58.9 to 128.9 |
| LDL_Choi_ | 7.8 | 7 to 9 | 37.1 | 29.4 to 54.6 | 10.3 | 5.5 to 14.5 | 126.9 | N/A | 19.4 | 14.2 to 22.3 | 78.5 | 54.1 to 116.8 |

(*Continue*)

**Supplementary Table S6**. (*Continue*)

|  | **Women** | | | | | | | | | | | |
| --- | --- | --- | --- | --- | --- | --- | --- | --- | --- | --- | --- | --- |
|  | **TG group 1 (TG < 175 mg/dL)** | | | | | | | | | | | |
|  | **Population 1 (Test n=2049)** | | | | **Population 2 (Test n=1546)** | | | | **Population 3 (Test n=0)** | | | |
| Limits of agreement | Lower | 95%CI | Upper | 95%CI | Lower | 95% CI | Upper | 95% CI | Lower | 95% CI | Upper | 95% CI |
| LDL_Friedwald_ | -26.4 | -26.8 to -25.9 | -3 | -3.5 to -2.6 | -27.1 | -27.7 to -26.3 | -3.9 | -4.6 to -3.3 | N/A | N/A | N/A | N/A |
| LDL_DeLong_ | -21.9 | -22.3 to -21.5 | 0.7 | 0.3 to 1.1 | -23.0 | -23.7 to -22.3 | 0.0 | -0.6 to 0.7 | N/A | N/A | N/A | N/A |
| LDL_Rao_ | -25.1 | -25.5 to -24.7 | -2.3 | -2.8 to -1.9 | -26 | -26.6 to -25.3 | -3.1 | -3.8 to -2.5 | N/A | N/A | N/A | N/A |
| LDL_Hattori_ | -33.8 | -34.3 to -33.3 | -8 | -8.5 to -7.5 | -35.1 | -35.8 to -34.2 | -8.1 | -8.9 to -7.3 | N/A | N/A | N/A | N/A |
| LDL_Anadaraja_ | -25.1 | -26.1 to -24 | 31.5 | 30.4 to 32.6 | -24 | -25.6 to -22.4 | 30.8 | 29.2 to 32.3 | N/A | N/A | N/A | N/A |
| LDL_Ahmadi_ | -49.0 | -50.8 to -47.2 | 45.5 | 43.7 to 47.3 | -52.6 | -55.4 to -49.7 | 46.2 | 43.3 to 49.1 | N/A | N/A | N/A | N/A |
| LDL_Puavilai_ | -22.5 | -22.9 to -22.1 | 0.1 | -0.3 to 0.6 | -23.5 | -24.2 to -22.8 | -0.6 | -1.3 to 0.1 | N/A | N/A | N/A | N/A |
| LDL_Vujovic_ | -21.1 | -21.5 to -20.6 | 1.5 | 1.1 to 1.9 | -22.2 | -22.9 to -21.5 | 0.9 | 0.2 to 0.6 | N/A | N/A | N/A | N/A |
| LDL_Chen and Zhang_ | -30.4 | -31.0 to -29.9 | -2.5 | -3 to -2 | -32.7 | -33.5 to -31.7 | -1.7 | -2.6 to -0.8 | N/A | N/A | N/A | N/A |
| LDL_de Cordova_ | -46.4 | -47.2 to -45.5 | -2.1 | -2.9 to -1.2 | -49.4 | -50.8 to -47.9 | 0.2 | -1.3 to 1.6 | N/A | N/A | N/A | N/A |
| LDL_Martin_ | -26.1 | -26.5 to -25.6 | -2.1 | -2.5 to -1.6 | -27.8 | -28.5 to -27.0 | -2.1 | -2.8 to -1.4 | N/A | N/A | N/A | N/A |
| LDL_Choi_ | -9.7 | -10.1 to -9.3 | 10.9 | 10.5 to 11.3 | -10.3 | -10.9 to -9.7 | 9.8 | 9.2 to 10.4 | N/A | N/A | N/A | N/A |
| Absolute percentage error | Med | 95% CI | 95th P | 95% CI | Med | 95% CI | 95th P | 95% CI | Med | 95% CI | 95th P | 95% CI |
| LDL_Friedwald_ | 13.2 | 13.0 to 13.5 | 24.2 | 23.6 to 25 | 14.0 | 13.5 to 14.4 | 25.5 | 24.1 to 26.9 | N/A | N/A | N/A | N/A |
| LDL_DeLong_ | 9.5 | 9.2 to 9.8 | 19.2 | 18.4 to 19.8 | 10.3 | 9.9 to 10.8 | 20.5 | 19.3 to 21.3 | N/A | N/A | N/A | N/A |
| LDL_Rao_ | 12.3 | 12 to 12.7 | 23.1 | 22.2 to 23.8 | 13.1 | 12.6 to 13.6 | 24.3 | 23.0 to 25.7 | N/A | N/A | N/A | N/A |
| LDL_Hattori_ | 18.6 | 18.3 to 18.8 | 29.1 | 28.6 to 29.8 | 19.3 | 18.9 to 19.8 | 30.2 | 29.1 to 31.8 | N/A | N/A | N/A | N/A |
| LDL_Anadaraja_ | 8.7 | 8.3 to 9.1 | 30.5 | 28.4 to 33.3 | 8.2 | 7.5 to 8.7 | 33.1 | 29.3 to 38 | N/A | N/A | N/A | N/A |
| LDL_Ahmadi_ | 16.5 | 15.8 to 17.4 | 48.6 | 45.6 to 51.9 | 17.7 | 16.5 to 18.8 | 47.6 | 44.9 to 54.4 | N/A | N/A | N/A | N/A |
| LDL_Puavilai_ | 10.0 | 9.7 to 10.3 | 19.6 | 19.0 to 20.7 | 10.8 | 10.4 to 11.2 | 21.2 | 19.8 to 22.2 | N/A | N/A | N/A | N/A |
| LDL_Vujovic_ | 8.8 | 8.5 to 9.1 | 18.2 | 17.5 to 18.9 | 9.5 | 9.2 to 9.8 | 19.3 | 18.3 to 20.6 | N/A | N/A | N/A | N/A |
| LDL_Chen and Zhang_ | 14.3 | 14.1 to 14.6 | 22.5 | 22.0 to 22.9 | 15.3 | 14.8 to 15.6 | 23.2 | 22.7 to 24.3 | N/A | N/A | N/A | N/A |
| LDL_de Cordova_ | 20.7 | 20.5 to 21.1 | 29.5 | 29.1 to 30 | 21.7 | 21.1 to 22.1 | 30.7 | 30.0 to 31.6 | N/A | N/A | N/A | N/A |
| LDL_Martin_ | 12.4 | 12.1 to 12.7 | 22.8 | 21.7 to 23.6 | 13.4 | 12.9 to 13.8 | 24.1 | 23.2 to 25.5 | N/A | N/A | N/A | N/A |
| LDL_Choi_ | 3.1 | 2.9 to 3.3 | 10.2 | 9.7 to 10.8 | 3.1 | 2.8 to 3.4 | 10.4 | 9.0 to 11.1 | N/A | N/A | N/A | N/A |

(*Continue*)

**Supplementary Table S6**. (*Continue*)

|  | **Women** | | | | | | | | | | | |
| --- | --- | --- | --- | --- | --- | --- | --- | --- | --- | --- | --- | --- |
|  | **TG group 2 (TG 175-400 mg/dL)** | | | | | | | | | | | |
|  | **Population 1 (Test n=511)** | | | | **Population 2 (Test n=206)** | | | | **Population 3 (Test n=269)** | | | |
| Limits of agreement | Lower | 95%CI | Upper | 95%CI | Lower | 95% CI | Upper | 95% CI | Lower | 95%CI | Upper | 95%CI |
| LDL_Friedwald_ | -38.1 | -39.2 to -36.9 | -7.5 | -8.7 to -6.3 | -36.3 | -38.1 to -34.4 | -6.1 | -7.9 to -4.3 | -29.9 | -32.3 to -27.4 | 16.3 | 13.9 to 18.8 |
| LDL_DeLong_ | -27.0 | -28.1 to -26.0 | 0.3 | -0.8 to 1.3 | -26.3 | -28.0 to -24.5 | 2.6 | 0.9 to 4.3 | -19.3 | -21.6 to -16.8 | 26.1 | 23.7 to 28.4 |
| LDL_Rao_ | -30.4 | -31.4 to -29.4 | -3.8 | -4.8 to -2.8 | -30.1 | -31.8 to -28.3 | -1.1 | -2.9 to 0.6 | -22.8 | -25.2 to -20.4 | 22.6 | 20.2 to 24.9 |
| LDL_Hattori_ | -45.0 | -46.2 to -43.8 | -13.5 | -14.7 to -12.3 | -43.2 | -45.1 to -41.3 | -11.9 | -13.8 to -10.0 | -36.7 | -39 to -34.3 | 7.9 | 5.6 to 2814 |
| LDL_Anadaraja_ | -41.8 | -43.7 to -40.0 | 7.2 | 5.4 to 0.1 | -37.8 | -40.6 to -35.0 | 8.6 | 5.8 to 11.4 | -30.3 | -32.8 to -27.8 | 16.9 | 14.4 to 19.4 |
| LDL_Ahmadi_ | 11.8 | 6.4 to 17.2 | 154.4 | 149 to 159.8 | 17.3 | 9.4 to 5.3 | 149.8 | 141.9 to 157.7 | 43.7 | 36.7 to 50.7 | 177.4 | 170.4 to 184.4 |
| LDL_Puavilai_ | -28.7 | -29.7 to -27.6 | -1.1 | -2.1 to 0.0 | -27.8 | -29.5 to -26.0 | 1.2 | -0.6 to 2.9 | -20.9 | -23.3 to -18.5 | 24.5 | 22.1 to 26.9 |
| LDL_Vujovic_ | -25.0 | -26.0 to -24.0 | 1.9 | 0.9 to 2.9 | -24.5 | -26.2 to -22.7 | 4.5 | 2.8 to 6.3 | -17.3 | -19.7 to -14.9 | 28.1 | 25.8 to 30.5 |
| LDL_Chen and Zhang_ | -29.2 | -30.3 to -28 | 1.3 | 0.1 to 2.4 | -29.1 | -31.1 to -27.1 | 4.4 | 2.4 to 6.5 | -20.4 | -22.8 to -18.0 | 24.3 | 21.9 to 26.6 |
| LDL_de Cordova_ | -37.7 | -39.6 to -35.8 | 13 | 11.1 to 15.0 | -37.0 | -40.2 to -33.8 | 15.8 | 12.6 to 19.0 | -26.0 | -28.9 to -22.9 | 30.7 | 27.7 to 33.7 |
| LDL_Martin_ | -26.1 | -27.3 to -25 | 3.7 | 2.6 to 4.9 | -26.0 | -28.0 to -24.0 | 7.3 | 5.3 to 9.3 | -17.5 | -19.9 to -15.1 | 27.1 | 24.8 to 29.5 |
| LDL_Choi_ | -12.7 | -13.7 to -11.7 | 12.6 | 11.7 to 13.6 | -12.2 | -13.9 to -10.5 | 15.4 | 13.7 to 17.0 | -5.5 | -7.9 to -3.2 | 39.2 | 36.8 to 41.5 |
| Absolute percentage error | Med | 95% CI | 95th P | 95% CI | Med | 95% CI | 95th P | 95% CI | Med | 95% CI | 95th P | 95% CI |
| LDL_Friedwald_ | 18.8 | 17.8 to 19.7 | 37.9 | 35.8 to 40.5 | 17.7 | 16.8 to 19.1 | 37.8 | 34.0 to 41.0 | 7.3 | 6.7 to 8.7 | 26.7 | 23.6 to 30.2 |
| LDL_DeLong_ | 11.2 | 10.6 to 11.7 | 24.4 | 22.5 to 25.8 | 10.5 | 9.7 to 11.2 | 23.5 | 21.0 to 25.2 | 5.7 | 5.0 to 6.4 | 22.0 | 19.8 to 31.1 |
| LDL_Rao_ | 14.3 | 13.6 to 15.2 | 28.7 | 26.8 to 30.1 | 13.6 | 12.9 to 14.6 | 28.0 | 25.2 to 31.1 | 5.7 | 5.1 to 6.6 | 20.9 | 18.3 to 28.7 |
| LDL_Hattori_ | 23.9 | 23.3 to 24.8 | 42.3 | 40.6 to 44.5 | 23.0 | 22.3 to 24.5 | 41.2 | 38.8 to 45.7 | 12.3 | 11 to 13.5 | 31.2 | 27.9 to 34.2 |
| LDL_Anadaraja_ | 15.0 | 13.9 to 15.9 | 34.5 | 32.7 to 38.7 | 12.7 | 11.6 to 14.7 | 31.0 | 27.0 to 34.0 | 8.0 | 6.8 to 8.8 | 26.4 | 22.7 to 31.1 |
| LDL_Ahmadi_ | 63.9 | 60.7 to 68.1 | 183.3 | 172.5 to 209.3 | 66.5 | 61.6 to 71.6 | 178.8 | 164.9 to 210.0 | 87.1 | 80.8 to 92.9 | 212.4 | 199.6 to 263.8 |
| LDL_Puavilai_ | 12.3 | 11.6 to 12.9 | 26.0 | 24.6 to 28.0 | 11.8 | 10.6 to 12.3 | 26.0 | 22.9 to 27.8 | 5.6 | 5.0 to 6.6 | 22.0 | 18.0 to 29.5 |
| LDL_Vujovic_ | 9.8 | 9.3 to 10.3 | 21.8 | 20.2 to 23.0 | 9.0 | 8.1 to 9.8 | 21.1 | 18.4 to 22.7 | 6.0 | 5.3 to 7.0 | 23.2 | 19.0 to 33.5 |
| LDL_Chen and Zhang_ | 11.9 | 11.5 to 12.4 | 19.3 | 18.6 to 20.3 | 10.3 | 9.2 to 11.5 | 19.3 | 17.7 to 20.9 | 5.3 | 4.6 to 6.0 | 20.7 | 17.2 to 30.4 |
| LDL_de Cordova_ | 11.6 | 10.7 to 12.3 | 20.0 | 19.7 to 21.6 | 9.9 | 8.9 to 11.1 | 20.4 | 19.1 to 23.4 | 7.3 | 6.3 to 8.4 | 29.1 | 24.5 to 38.1 |
| LDL_Martin_ | 9.6 | 9.2 to 10.1 | 17.4 | 16.8 to 18.9 | 8.3 | 7.1 to 9.3 | 17.1 | 15.4 to 19.7 | 5.2 | 4.6 to 6.2 | 22.3 | 18.9 to 33.2 |
| LDL_Choi_ | 3.1 | 2.7 to 3.4 | 11.2 | 9.5 to 13.7 | 4.3 | 3.7 to 4.6 | 13.1 | 11.3 to 17.7 | 13.6 | 12.3 to 14.6 | 34.0 | 28.0 to 45.3 |

(*Continue*)

**Supplementary Table S6**. (*Continue*)

|  | Women | | | | | | | | | | | |
| --- | --- | --- | --- | --- | --- | --- | --- | --- | --- | --- | --- | --- |
|  | TG group 3 (TG > 400 mg/dL) | | | | | | | | | | | |
|  | Population 1 (Test n=68) | | | | Population 2 (Test n=18) | | | | Population 3 (Test n=32) | | | |
| Limits of agreement | Lower | 95% CI | Upper | 95% CI | Lower | 95% CI | Upper | 95% CI | Lower | 95% CI | Upper | 95% CI |
| LDL_Friedwald_ | -81.3 | -88.7 to -73.9 | -11.4 | -18.8 to -4.0 | -181.1 | -235.9 to -126.2 | 67 | 12.2 to 121.7 | -80.3 | -100.1 to -60.4 | 44.6 | 24.8 to 64.5 |
| LDL_DeLong_ | -55.5 | -62.0 to -49.1 | 5.5 | -0.9 to 12.0 | -83.9 | -109.5 to -58.2 | 32.2 | 6.6 to 57.9 | -60.1 | -80.3 to -39.8 | 67.6 | 47.3 to 87.9 |
| LDL_Rao_ | -49.5 | -56.9 to -42.1 | 20.2 | 12.8 to 27.6 | -714.9 | -1071 to -358 | 899 | 542.4 to 1255.5 | -63.8 | -88.9 to -38.7 | 94.3 | 69.1 to 119.4 |
| LDL_Hattori_ | -85.6 | -92.7 to -78.4 | -18.0 | -25.2 to -10.8 | -180.7 | -232.9 to -128.4 | 55.6 | 3.4 to 107.8 | -82.2 | -100.6 to -63.7 | 33.8 | 15.4 to 52.3 |
| LDL_Anadaraja_ | -84.8 | -92.6 to -76.9 | -10.7 | -18.6 to -2.9 | -158.5 | -204.2 to -112.7 | 48.5 | 2.8 to 94.3 | -73.1 | -89.9 to -56.3 | 32.8 | 16.0 to 49.7 |
| LDL_Ahmadi_ | 94.9 | 57.5 to 32.4 | 448.1 | 410.7 to 485.6 | -695.8 | -1195 to -196 | 1565.7 | 1065.9 to 2065 | 90.7 | 23 to 58.4 | 516.8 | 449.1 to 584.5 |
| LDL_Puavilai_ | -59.5 | -66.1 to -52.9 | 2.6 | -4.0 to 9.1 | -99.7 | -130 to -69.3 | 37.7 | 7.3 to 68.1 | -63.3 | -83.5 to -43.1 | 63.9 | 43.7 to 84.1 |
| LDL_Vujovic_ | -50.8 | -57.2 to -44.5 | 9.3 | 3.0 to 5.7 | -65.5 | -85.8 to -45.2 | 26.5 | 6.2 to 46.8 | -56.2 | -76.6 to -35.7 | 72.4 | 51.9 to 92.8 |
| LDL_Chen and Zhang_ | -41.0 | -47.4 to -34.6 | 19.1 | 12.7 to 25.5 | -47.6 | -68.2 to -27 | 45.7 | 25.1 to 66.3 | -44.7 | -64.2 to -25.1 | 78.2 | 58.7 to 97.7 |
| LDL_de Cordova_ | -29.6 | -39.2 to -20.0 | 60.9 | 51.3 to 70.5 | -143.1 | -225.9 to -60.2 | 232 | 149.1 to 314.9 | -27.2 | -49.1 to -5.2 | 111.0 | 89.1 to 33 |
| LDL_Martin_ | -37.5 | -43.9 to -31.0 | 23.3 | 16.9 to 29.8 | -53.2 | -73.3 to -33.1 | 37.9 | 17.7 to 58.0 | -37.6 | -56.2 to -19.1 | 79.4 | 60.8 to 98 |
| LDL_Choi_ | -33.4 | -39.6 to -27.1 | 25.2 | 19.0 to 31.4 | -26.0 | -38.8 to -13.2 | 32 | 19.2 to 44.8 | -40.6 | -61.5 to -19.7 | 90.9 | 70.0 to 111.7 |
| Absolute percentage error | Med | 95% CI | 95th P | 95% CI | Med | 95% CI | 95th P | 95% CI | Med | 95% CI | 95th P | 95% CI |
| LDL_Friedwald_ | 39.9 | 32.8 to 44.8 | 88.2 | N/A | 31.0 | 17.9 to 49.1 | 460.2 | N/A | 21.7 | 10.9 to 33.9 | 98.8 | N/A |
| LDL_DeLong_ | 20.6 | 17.3 to 24.8 | 60.9 | N/A | 13.6 | 7.2 to 28.8 | 195.1 | N/A | 14.1 | 6.5 to 21.8 | 50.3 | N/A |
| LDL_Rao_ | 15.4 | 12.5 to 18.0 | 32.0 | N/A | 10.4 | 5.7 to 15.9 | 2557.7 | N/A | 13.5 | 9.2 to 22.2 | 47.7 | N/A |
| LDL_Hattori_ | 44.2 | 37.7 to 48.9 | 91.5 | N/A | 36.3 | 23.5 to 52.9 | 452.7 | N/A | 25.5 | 16.4 to 39 | 91.7 | N/A |
| LDL_Anadaraja_ | 37.0 | 32.9 to 43.3 | 100.9 | N/A | 36.1 | 16.7 to 49.8 | 394.7 | N/A | 23.4 | 15.4 to 31.1 | 98.4 | N/A |
| LDL_Ahmadi_ | 216.4 | 188.1 to 243.5 | 626.2 | N/A | 237.9 | 177.7 to 284.6 | 4240.3 | N/A | 290.5 | 258 to 344.5 | 883.9 | N/A |
| LDL_Puavilai_ | 24.5 | 20.8 to 28.5 | 59.0 | N/A | 16.5 | 8.8 to 32.2 | 239.8 | N/A | 14.5 | 5.3 to 22.6 | 54.4 | N/A |
| LDL_Vujovic_ | 18.2 | 14.2 to 20.7 | 56.1 | N/A | 10.8 | 7.2 to 24.4 | 141.3 | N/A | 12.6 | 9.3 to 18.0 | 49.9 | N/A |
| LDL_Chen and Zhang_ | 12.2 | 10.4 to 14.0 | 21.9 | N/A | 8.7 | 5.0 to 13.7 | 127.2 | N/A | 14.9 | 8.1 to 23.5 | 56.7 | N/A |
| LDL_de Cordova_ | 11.8 | 9.0 to 16.9 | 80.3 | N/A | 20.5 | 4.2 to 30.9 | 654.0 | N/A | 39.9 | 24.4 to 60.7 | 188.3 | N/A |
| LDL_Martin_ | 9.3 | 6.5 to 11.2 | 31.0 | N/A | 8.1 | 5.0 to 19.1 | 114.7 | N/A | 13.0 | 10.1 to 30.8 | 67.7 | N/A |
| LDL_Choi_ | 7.6 | 5.6 to 9.2 | 24.3 | N/A | 8.3 | 5.0 to 15.1 | 39.0 | N/A | 21.0 | 15.1 to 29.6 | 63.5 | N/A |

*95^th^ P* 95th percentile, *Med* Median, *N/A* Not available because of limited numbers of test results

Top three equations are presented in color: red represents the lowest, orange represents the second, and yellow represents the third lowest absolute percentage error.

**Supplementary Figure Legends**

**Supplementary Fig. S1** Bland–Altman plots for the 12 equations with directly measured LDL concentration

**Supplementary Fig. S2** Overall agreement of categorization according to the NCEP ATP III between calculated LDL and directly measured LDL by LDL subgroup

**Supplementary Fig. S3** Overall agreement of categorization according to the NCEP ATP III between calculated LDL and directly measured LDL by TG subgroup
