# Supplementary figures and images for: Validation of multiple equations for estimating low-density lipoprotein cholesterol levels in Korean adults
Source: Lipids Health Dis. 2021 Sep 20;20:111. doi: 10.1186/s12944-021-01525-6 (PMC8453999; doi:10.1186/s12944-021-01525-6)

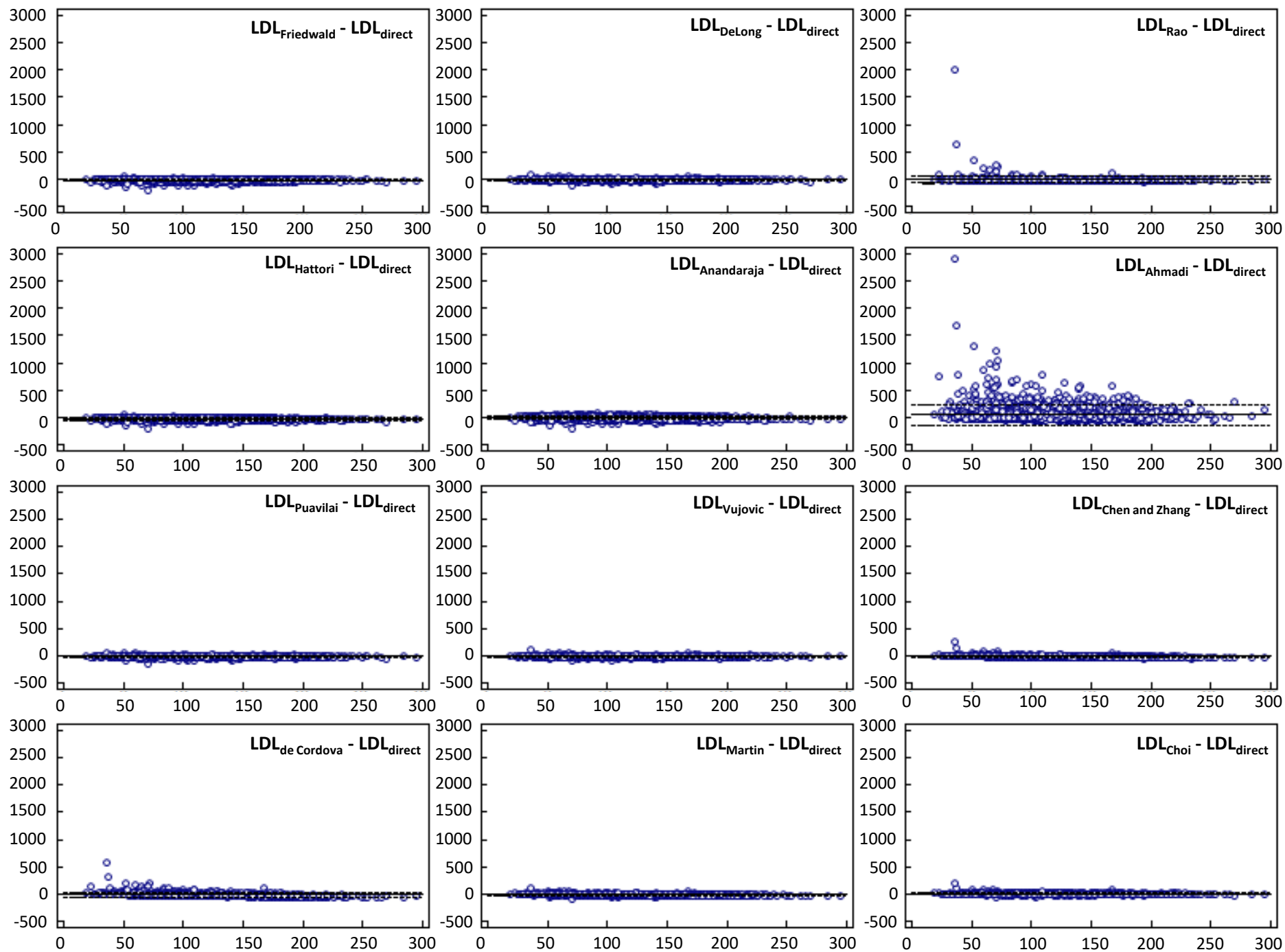

Supplement: Supplementary file 2 — Additional file 2: Supplementary Table S1. Intraclass correlation coefficient and systemic differences among the 12 equations in comparison with directly measured LDL. Supplementary Table S2. Limits of agreement and absolute error among the 12 equations in comparison with directly measured LDL. Supplementary Table S3. Intraclass correlation coefficient and systemic differences among the 12 equations in comparison with directly measured LDL (LDLdirect) by subgroup of LDL concentration. Supplementary Table S4. Limits of agreement and absolute error among the 12 equations in comparison with directly measured LDL by subgroup of LDL concentration. Supplementary Table S5. Intraclass correlation coefficient and systemic differences among the 12 equations in comparison with directly measured LDL according to subgroup by triglyceride (TG) concentration. Supplementary Table S6. Limit of agreement and absolute percentage errors among the 12 equations in comparison with directly measured LDL according to subgroups by triglyceride (TG) concentration. Supplementary Fig. S1. Bland–Altman plots for the 12 equations with directly measured LDL concentration. Supplementary Fig. S2. Overall agreement of categorization according to the NCEP ATP III between calculated LDL and directly measured LDL by LDL subgroup. Supplementary Fig. S3. Overall agreement of categorization according to the NCEP ATP III between calculated LDL and directly measured LDL by TG subgroup. [file 12944_2021_1525_MOESM2_ESM.zip › Supple Figure S1 R1.pdf]

## Population 1

## Population 2

## Population 3

### Total

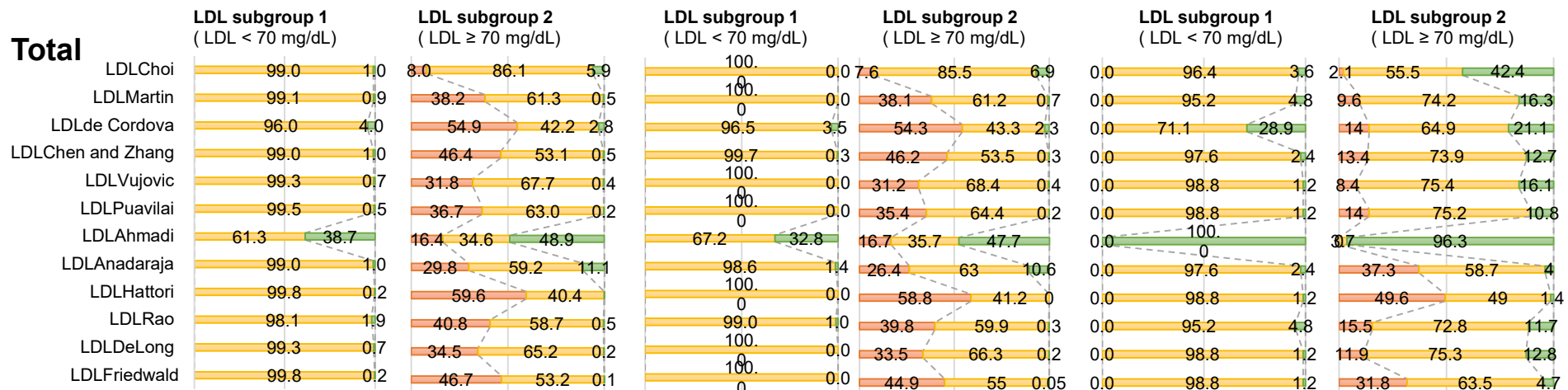

### Men

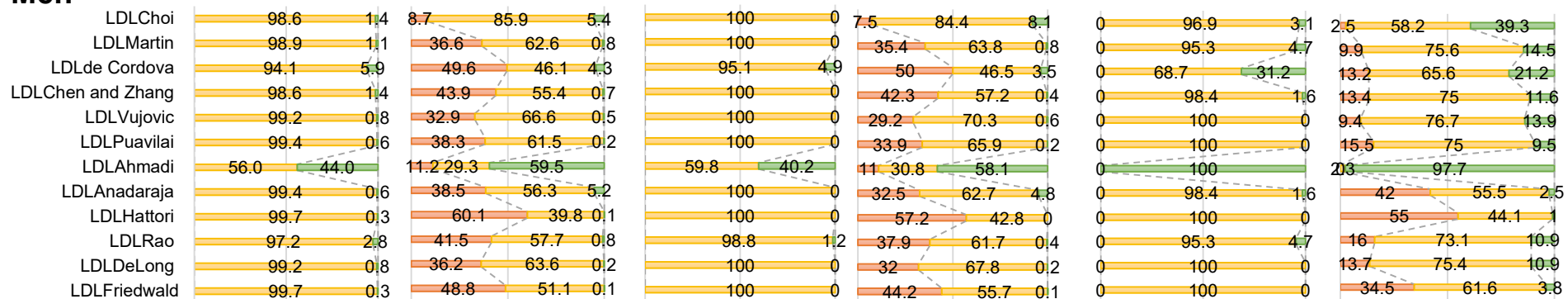

### Women

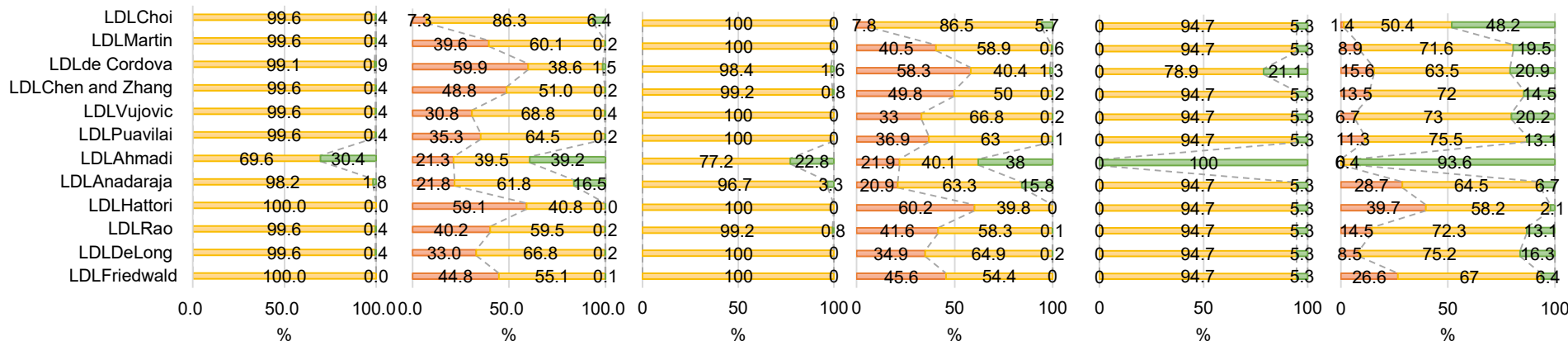

Supplement: Supplementary file 2 — Additional file 2: Supplementary Table S1. Intraclass correlation coefficient and systemic differences among the 12 equations in comparison with directly measured LDL. Supplementary Table S2. Limits of agreement and absolute error among the 12 equations in comparison with directly measured LDL. Supplementary Table S3. Intraclass correlation coefficient and systemic differences among the 12 equations in comparison with directly measured LDL (LDLdirect) by subgroup of LDL concentration. Supplementary Table S4. Limits of agreement and absolute error among the 12 equations in comparison with directly measured LDL by subgroup of LDL concentration. Supplementary Table S5. Intraclass correlation coefficient and systemic differences among the 12 equations in comparison with directly measured LDL according to subgroup by triglyceride (TG) concentration. Supplementary Table S6. Limit of agreement and absolute percentage errors among the 12 equations in comparison with directly measured LDL according to subgroups by triglyceride (TG) concentration. Supplementary Fig. S1. Bland–Altman plots for the 12 equations with directly measured LDL concentration. Supplementary Fig. S2. Overall agreement of categorization according to the NCEP ATP III between calculated LDL and directly measured LDL by LDL subgroup. Supplementary Fig. S3. Overall agreement of categorization according to the NCEP ATP III between calculated LDL and directly measured LDL by TG subgroup. [file 12944_2021_1525_MOESM2_ESM.zip › Supple Figure S2_R1.pdf]

## Population 1

## Population 2

## Population 3

### Total

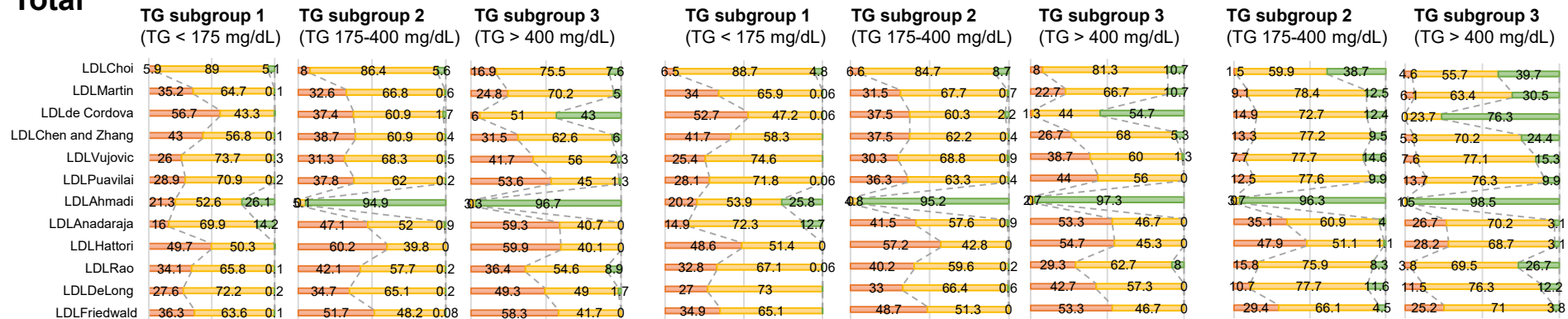

### Men

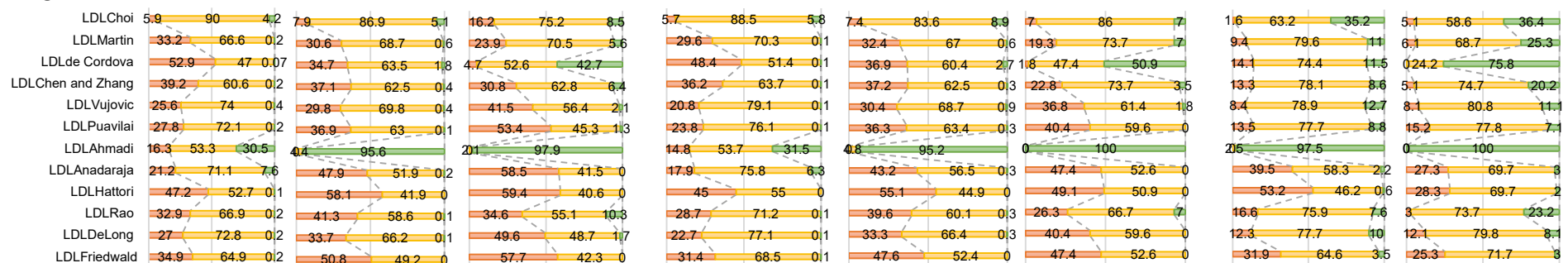

### Women

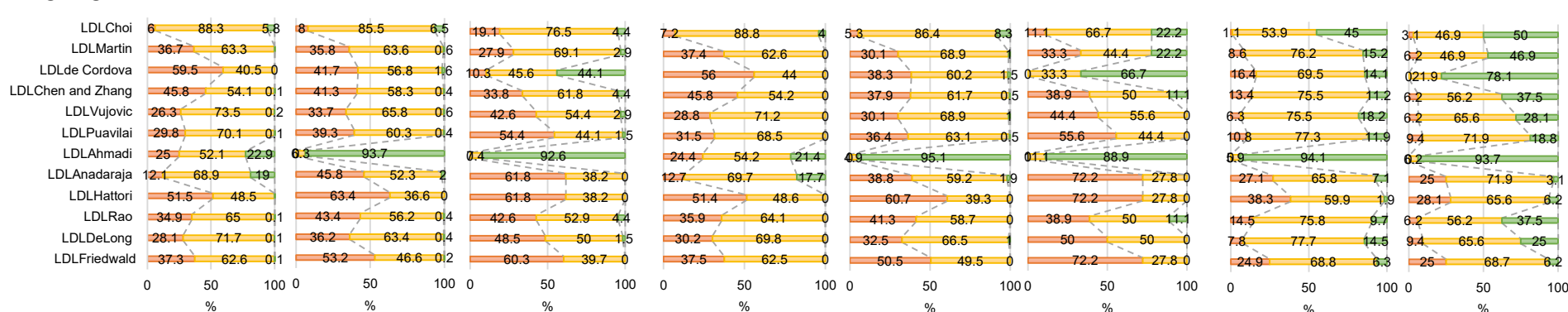

Supplement: Supplementary file 2 — Additional file 2: Supplementary Table S1. Intraclass correlation coefficient and systemic differences among the 12 equations in comparison with directly measured LDL. Supplementary Table S2. Limits of agreement and absolute error among the 12 equations in comparison with directly measured LDL. Supplementary Table S3. Intraclass correlation coefficient and systemic differences among the 12 equations in comparison with directly measured LDL (LDLdirect) by subgroup of LDL concentration. Supplementary Table S4. Limits of agreement and absolute error among the 12 equations in comparison with directly measured LDL by subgroup of LDL concentration. Supplementary Table S5. Intraclass correlation coefficient and systemic differences among the 12 equations in comparison with directly measured LDL according to subgroup by triglyceride (TG) concentration. Supplementary Table S6. Limit of agreement and absolute percentage errors among the 12 equations in comparison with directly measured LDL according to subgroups by triglyceride (TG) concentration. Supplementary Fig. S1. Bland–Altman plots for the 12 equations with directly measured LDL concentration. Supplementary Fig. S2. Overall agreement of categorization according to the NCEP ATP III between calculated LDL and directly measured LDL by LDL subgroup. Supplementary Fig. S3. Overall agreement of categorization according to the NCEP ATP III between calculated LDL and directly measured LDL by TG subgroup. [file 12944_2021_1525_MOESM2_ESM.zip › Supple Figure S3_R1.pdf]
